# A dual-responsive cationic acridinium nanohoop: redox activity and acid/base-controlled reversible guest capture and release

**DOI:** 10.1039/d6sc01132b

**Published:** 2026-04-13

**Authors:** Jiao Ming, Jin Zhang, Yan Hu, Kai Lan, Dongmei Zhang, Ping Li, Jiarong Miao, Jiyong Jiang, Xiaobo Zhang, Hanchi Zhong, Peiyuan Yu, Chuyang Cheng

**Affiliations:** a Key Laboratory of Green Chemistry & Technology, Sichuan University Chengdu 610064 China cycheng@scu.edu.cn; b Department of Chemistry, Guangming Advanced Research Institute, Southern University of Science and Technology Shenzhen 518055 China yupy@sustech.edu.cn

## Abstract

In this work, we report a fully conjugated, radially cationic nitrogen-doped cycloparaphenylene derivative, Ad[10]CPP^+^, constructed using the acridinium moiety as a functional building block. This compound exhibits remarkable reversibility in structural transformations under chemical stimuli and tunable redox behavior under electrochemical stimuli. Cyclic voltammetry reveals that Ad[10]CPP^+^ possesses multiple oxidation states and demonstrates significant redox versatility. Furthermore, Ad[10]CPP^+^ can reversibly transform from a near-circular configuration to a water-droplet shape upon hydroxide attack on the acridinium moiety when the acid concentration is lowered. The host–guest interaction between fullerene (C_60_) and Ad[10]CPP^+^ was confirmed by NMR spectroscopy and single-crystal X-ray diffraction. Significantly, the host–guest system exhibits excellent fatigue resistance, enabling at least five consecutive cycles of chemically triggered fullerene capture and release through reversible structural transformations.

## Abstract 2

A full conjugated, strained macrocycle with a cationic nitrogen at the phenylene linkage reversibly switches between its cationic and neutral state upon acid concentration change, enabling reversible capture and release of C_60_.

## Graphical abstract

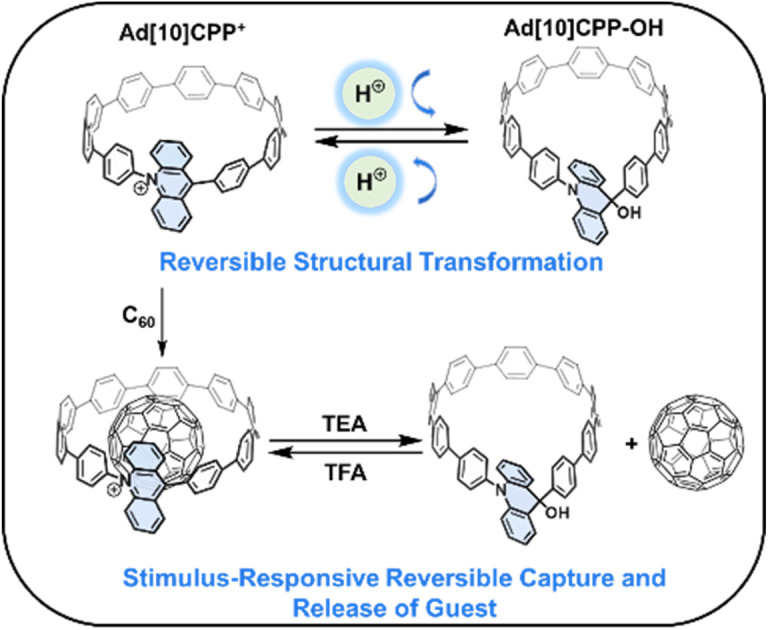

## Introduction

**Scheme 1:**
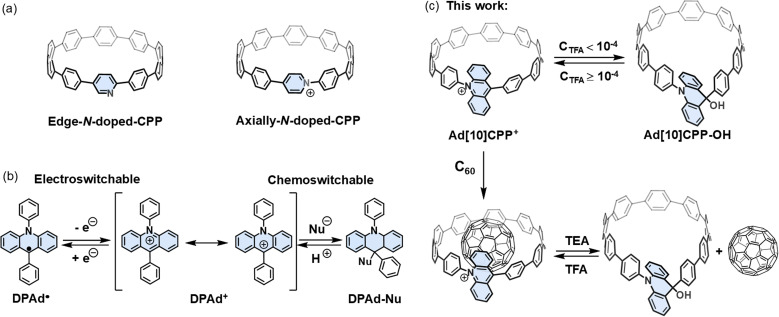
Design concept of an acridinium functionalized CPP. (a) Edge-*N*-doped CPP and axially-*N*-CPP. (b) Reversible structural transformation of DPAd^+^ under electrochemical and chemical stimuli. (c) Reversible structural transformation and guest capture/release of Ad[10]CPP^+^ under chemical stimuli in this work.

Cycloparaphenylenes (CPPs), aromatic macrocycles composed of *para*-phenylene units, have garnered considerable attention since their first synthesis.^1–8^ The interest is primarily attributed to their inherent ring strain^9–15^ and unique radial cyclic π-conjugated architectures.^16–21^ Over the past decade, the field has witnessed remarkable progress, as evidenced by the successful synthesis of CPPs and their derivatives of varying sizes.^22–28^ Despite these advancements, the incorporation of heteroatoms, particularly nitrogen, remains underexplored.^8^ Replacing phenylene units with nitrogen-containing aromatic rings can endow CPPs with acid–base responsiveness,^29^ metal coordination capabilities,^30^ and synthetic handles for constructing mechanically interlocked molecules.^31–33^ Subsequent alkylation of these nitrogen atoms can convert the neutral macrocycles into cationic ones, introducing redox activity.^34,35^ Both the amount and position of nitrogen doping can effectively modulate the photophysical^36^ and supramolecular properties of CPPs.^37^ Notably, in most reported cases, nitrogen atoms are incorporated at lateral positions of the CPP skeleton (Scheme 1a, left), as this approach aligns with conventional synthetic routes. In contrast, the direct fusion of nitrogen at the phenylene linkage sites, namely, axial doping, is far less common.^38–42^ Such a configuration is predicted to markedly influence the nanoring's photophysical profile, introduce multiple accessible redox states,^38^ and create a cavity with tunable size.^39^ However, the limited examples of axially *N*-doped carbon nanorings reported to date are, strictly speaking, not fully conjugated CPPs. Recently, we reported the first fully conjugated cationic CPPs bearing pyridinium units at the phenylene linkages^43^ (Scheme 1a, right), a long-standing synthetic challenge due to ring strain and cation instability under basic conditions. This synthetic challenge was effectively overcome by introducing an *N*-phenylpiperidine derivative strain-release unit followed by aromatization to construct a pyridinium moiety. In the present work, we extend this design by employing a π-expanded motif, in the form of an acridine fragment, as a strain-release unit. This approach not only enhances synthetic accessibility but also unlocks distinct functional advances. Specifically, we demonstrate that installing cationic nitrogen at the phenylene linkage sites in the form of acridinium enables reversible, stimuli-responsive switching of the macrocyclic framework between configurations in response to electrochemical and acid–base stimuli. This structural dynamism, in turn, facilitates controlled capture and release of guest molecules, advancing CPPs toward adaptive host–guest systems.

The rigid and curved conjugated structures of CPPs serve as ideal host molecules for fullerenes.^44–50^ Their complementary shape and size facilitate strong π–π interactions with fullerenes, achieving binding constants of 10^5^–10^6^ M^−1^. The rigid and strained macrocyclic constitution, however, also poses a challenge for incorporating reversible guest capture and release functionality. Introducing stimuli-responsive motifs that can reversibly modulate the strained framework offers an effective strategy for achieving controlled guest capture and release. While several studies have incorporated stimuli-responsive motifs into CPPs to modulate the geometry of the strained macrocycle,^16,51–54^ to the best of our knowledge, no examples of stimuli-responsive guest capture and release for CPPs have been reported thus far.

The acridinium unit is known for its reversible structural transformation in response to electrochemical and chemical stimuli.^55–63^ Specifically, 9,10-diphenylacridinium (DPAd^+^) is highly susceptible to nucleophilic attack and can reversibly transition between its radical state and cationic form under electrochemical control (Scheme 1b). In this study, we incorporate a DPAd^+^ fragment into the CPP skeleton to construct a functionalized strain macrocycle similar in size to [10]CPP, namely, Ad[10]CPP^+^ (Scheme 1c). We carried out a comprehensive investigation into the synthesis and structural, optical, electronic, and computational properties of Ad[10]CPP^+^. Our findings revealed that Ad[10]CPP^+^ undergoes reversible structural transformations under electrochemical and acid/base stimulation. Furthermore, we performed extensive studies on the host–guest chemistry of Ad[10]CPP^+^ and successfully obtained the solid-state suprastructure of C_60_ captured by Ad[10]CPP^+^. Most notably, Ad[10]CPP^+^ demonstrated the ability to bind and release C_60_ in response to acid/base stimuli, exhibiting excellent fatigue resistance over multiple cycles. This approach offers significant potential for expanding the applications of CPPs in supramolecular chemistry and materials science.

## Results and discussion

### Synthesis and structure characterization

**Fig. 1:**
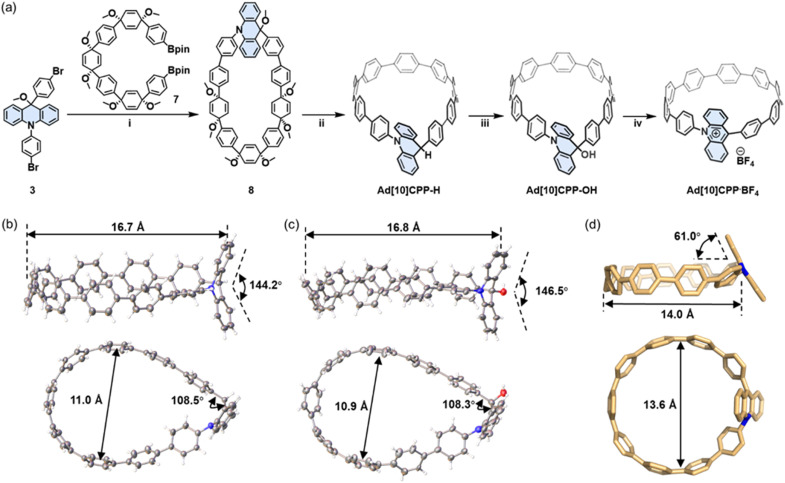
(a) The synthetic route of Ad[10]CPP·BF_4_. Reaction conditions: (i) Pd(PPh_3_)_4_, NaHCO_3_, *n*-Bu_4_NBr, toluene/methanol/H_2_O, 90 °C, 24 h. (ii) SnCl_2_·2H_2_O, concentrated HCl, THF, rt, 12 h, 13% over two steps. (iii) DDQ, CH_2_Cl_2_, rt, 12 h, 54%. (iv) HBF_4_ (40% aq), CH_2_Cl_2_, rt, 3 h, quantitative by NMR. The ORTEP drawing of X-ray solid-state structures of (b) Ad[10]CPP-H and (c) Ad[10]CPP-OH. Thermal ellipsoids are set at 30% probability. Grey C, white H, blue N, and red O. Solvent molecules are omitted for clarity. For crystallographic details and CCDC numbers see the SI. (d) The DFT optimized geometry of the Ad[10]CPP^+^ cation (at the B3LYP-D3(BJ)/def2-SVP level). Color code: C yellow, N blue, O red and H white. Other hydrogen atoms, solvent molecules and counter ions are omitted for the sake of clarity.

**Fig. 2:**
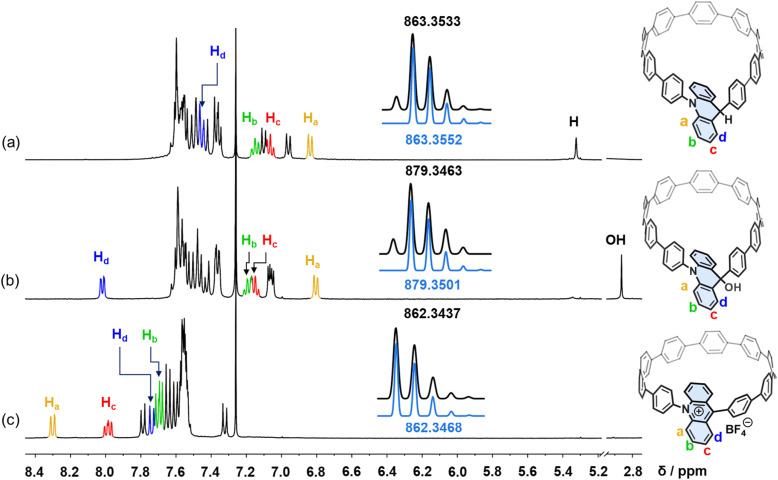
Partial ^1^H NMR spectra (400 MHz, CDCl_3_, 298 K) of (a) Ad[10]CPP-H, (b) Ad[10]CPP-OH, and (c) Ad[10]CPP-OH upon addition of excess aqueous HBF_4_ (40%). Insets show the experimental (black) and simulated (blue) MALDI-TOF-HRMS data for Ad[10]CPP-H, Ad[10]CPP-OH, and Ad[10]CPP^+^.

The synthesis of the target molecule commenced with a macrocyclization step through a double-site Suzuki–Miyaura cross-coupling reaction between the bent dibromoacridine derivative 3 and the C-shaped diboronate^15,64^7 (Fig. 1a). Our initial strategy anticipated that the SnCl_2_-mediated reductive aromatization^65^ would yield the desired acridinium product. However, the compound obtained did not align with our expectations; the ^1^H NMR spectrum exhibited an unanticipated signal at 5.32 ppm, devoid of the characteristic deshielded aromatic signals above 8 ppm (Fig. 2a and S8). Moreover, the mass spectrometry data deviated by approximately one unit (Fig. S21). Single-crystal X-ray diffraction analysis ultimately confirmed that the product was a macrocycle with the acridinium unit hydrogenated (Ad[10]CPP-H), featuring an sp^3^-hybridized carbon within the acridine moiety, which imparted a teardrop shape to the macrocycle with dimensions of 16.7 Å along the long axis and 11.0 Å along the short axis (Fig. 1b). The over-reduction, while not the desired outcome, was not detrimental, as the product remained a macrocycle with a structure closely resembling that of our target. Subsequently, we endeavored to oxidize Ad[10]CPP-H using 2,3-dichloro-5,6-dicyano-1,4-benzoquinone (DDQ) to achieve the acridinium macrocycle. Despite our efforts, the product again closely resembled the expected macrocycle but exhibited an additional signal at 2.86 ppm in the ^1^H NMR spectrum, with the mass data offset by around 17 units (Fig. 2b, S11 and S22). Single-crystal X-ray diffraction revealed a macrocycle of similar teardrop shape (Ad[10]CPP-OH), with the only difference being the oxidation of a C–H bond to a C–OH group compared with Ad[10]CPP-H (Fig. 1c).

With the macrocycle in the anticipated oxidative state, we proceeded to acidify the sample using HBF_4_ to protonate the hydroxy group and induce dehydration, aiming to convert the macrocycle into the cationic acridinium-functionalized final product (Ad[10]CPP·BF_4_). The ^1^H NMR spectrum demonstrated a complete transformation from Ad[10]CPP-OH to a new species, with the characteristic deshielded aromatic signal for *H*_a_ at 8.30 ppm and *H*_c_ at 7.99 ppm (Fig. 2c and S15). The high resolution MALDI-TOF MS spectrum confirmed the expected molecular weight of the acridinium macrocyclic cation (Fig. S23), thereby validating the successful synthesis of the desired macrocycle. Ad[10]CPP·TFA is also obtained by treating Ad[10]CPP-OH with excess trifluoracetic acid (TFA). The isolation of a solid Ad[10]CPP·BF_4_ or Ad[10]CPP·TFA sample proved to be elusive despite extensive efforts, attributed to the intrinsic instability of the cationic macrocycle under neutral conditions. Specifically, the acridinium moiety is highly vulnerable to nucleophilic attack, readily reverting to Ad[10]CPP-OH upon exposure to moisture. A TFA titration experiment revealed that the interconversion between Ad[10]CPP^+^ and Ad[10]CPP-OH occurs within the TFA range between 1.0 × 10^−4^ and 1.0 × 10^−5^ M in CH_2_Cl_2_ (Fig. S26). This highlights the dependency of the thermodynamic stability of the cationic macrocycle on acidic conditions. Strain energy calculations revealed a significant increase from 38.7 kcal mol^−1^ for Ad[10]CPP-OH to 56.2 kcal mol^−1^ for Ad[10]CPP^+^. This substantial increase in ring strain imposes a considerable thermodynamic penalty, thereby disfavoring the formation of the cationic species (Fig. S73 and Table S5). Furthermore, the instability also hinders the crystal growth of the cationic macrocycle. Specifically, the gradual evaporation of the volatile acid (*e.g.*, TFA), which is essential for maintaining the cationic state, prevented the growth of diffraction-quality crystals over time. Despite months of dedicated efforts, including crystallization attempts in a nitrogen-filled glovebox, we were unable to obtain a single crystal suitable for X-ray diffraction analysis. Consequently, we employed density functional theory (DFT) calculations at the B3LYP-D3(BJ)/def2-SVP level to elucidate the structural characteristics of the acridinium macrocycle. The computational results depicted an elliptical nanoring with a long axis of 14.0 Å and a short axis of 13.6 Å. The acridinium aromatic plane is oriented at an angle of 61.0° relative to the macrocyclic plane (Fig. 1d). The torsion angles between the acridinium unit and its adjacent phenylene moieties were measured to be 51.5° and 67.3°, respectively. These values are notably larger than those between other phenylene rings (22.1° to 36.5°), a difference attributed to the steric hindrance between the close-contacted hydrogen atoms on acridinium and adjacent phenylene units (Fig. S76).

### Photophysical properties

**Fig. 3:**
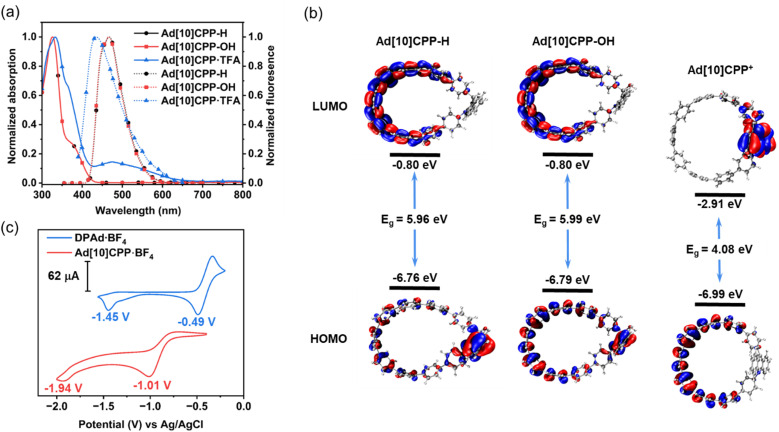
(a) UV-vis absorption (solid line) and fluorescence emission (dotted line) spectra of Ad[10]CPP-H (*λ*_ex_ = 325 nm) (black circle), Ad[10]CPP-OH (*λ*_ex_ = 325 nm) (red square), and Ad[10]CPP·TFA (blue triangle) measured in CH_2_Cl_2_ solutions (1 × 10^−5^ M) at room temperature. The Ad[10]CPP·TFA sample is prepared by adding excess trifluoroacetic acid into Ad[10]CPP-OH, maintaining acid concentration at 1.0 × 10^−3^ M. (b) The DFT calculated frontier molecular orbitals and energy levels of Ad[10]CPP-H, Ad[10]CPP-OH and Ad[10]CPP^+^. (c) CV of 1.0 mM DPAd·BF_4_ in CH_2_Cl_2_ solution and Ad[10]CPP·BF_4_ in CH_3_CN solution (Ag/AgCl, 0.1 M *n*-Bu_4_NBF_4_, 100 mV s^−1^).

With the acridinium macrocycle in hand, we proceeded to investigate the photophysical properties of this compound together with its two precursor macrocycles (Fig. 3a and Table S1). The absorption spectra of Ad[10]CPP-H and Ad[10]CPP-OH were nearly identical, both exhibiting overlapping absorption bands between 300 and 440 nm and lacking a broad charge-transfer band. Both compounds showed an absorption maximum (*λ*_max_) at 326 nm, accompanied by a shoulder at approximately 378 nm. Time-dependent density functional theory (TD-DFT) calculations indicate that the major absorption at 326 nm arises from a combination of transitions: for Ad[10]CPP-H, these are HOMO−2 → LUMO, HOMO−1 → LUMO+1 and HOMO → LUMO+1 (Table S6); for Ad[10]CPP-OH, they are HOMO−2 → LUMO and HOMO → LUMO+1 (Table S7). In both Ad[10]CPP-H and Ad[10]CPP-OH, the HOMO is primarily confined to the oligoparaphenylene loop, indicating that the acridine segment acts as an electron donor in these neutral systems. A more pronounced spectroscopic change was observed upon protonating Ad[10]CPP-OH to form Ad[10]CPP^+^. The absorption maximum *λ*_max_ of Ad[10]CPP^+^ was observed at 332 nm (Fig. 3a), which is slightly blue-shifted compared to [10]CPP (*λ*_max_ = 341 nm)^66^ but red-shifted relative to Ad[10]CPP-OH (*λ*_max_ = 326 nm). The main absorption band of Ad[10]CPP^+^ is attributed to HOMO−1 → LUMO+1 and HOMO → LUMO+2 transitions (Table S8). Additionally, a shoulder peak appeared at around 365 nm, along with a broad characteristic charge-transfer band spanning from 420 nm to nearly 700 nm. TD-DFT calculations attribute this broad band to several transitions, including HOMO−4 → LUMO, HOMO−2 → LUMO, HOMO−1 → LUMO, and HOMO → LUMO. The frontier molecular orbital calculations further reveal that the HOMO of Ad[10]CPP^+^ is localized on the oligoparaphenylene loop, while the LUMO is confined to the acridinium unit and its adjacent aromatic rings (Fig. 3b). This distinct spatial separation of orbitals is characteristic of a donor–acceptor system. Compared to Ad[10]CPP-OH, the HOMO and LUMO energy levels of Ad[10]CPP^+^ are lowered by approximately 2.11 eV and 0.2 eV (Fig. 3b). This decrease in frontier molecular orbital energy is attributed to the formation of a rigid cationic framework and electron delocalization within the acridinium fragment, which significantly stabilizes the entire π-system, particularly the HOMO.

The fluorescence properties of Ad[10]CPP-H and Ad[10]CPP-OH were found to be similar. Both exhibited emission bands at nearly identical wavelengths (*λ*_em_ = 467 nm for Ad[10]CPP-H and 466 nm for Ad[10]CPP-OH; Fig. 3a) and high absolute fluorescence quantum yields (*Φ*_F_ = 70.9% for Ad[10]CPP-H and 67.3% for Ad[10]CPP-OH; Fig. S32 and S33). Their singlet excited-state lifetimes (*τ*) were measured *via* time-resolved fluorescence spectroscopy to be 2.48 ns for Ad[10]CPP-H and 2.40 ns for Ad[10]CPP-OH, respectively (Fig. S35 and S36). A pronounced change was observed upon protonation of Ad[10]CPP-OH. The gradual addition of TFA to Ad[10]CPP-OH led to significant fluorescence quenching, with emission nearly completely suppressed upon full conversion to Ad[10]CPP^+^ (Fig. S27). The cationic species Ad[10]CPP^+^ itself displayed very weak fluorescence with an emission maximum blue-shifted to 435 nm (Fig. 3a). This weak emission further decreased in intensity and underwent an additional blue shift as the concentration of TFA was further elevated (Fig. S28). The stability of Ad[10]CPP^+^ is contingent on excess acid, a condition that inevitably modifies its photophysical behavior, resulting in a pronounced acid concentration-dependent blue shift emission. This phenomenon likely arises because the acidic environment alters the excited-state decay pathway, potentially favoring radiative transition from a higher-energy localized excited (LE) state on the acridinium moiety (Fig. S30). The absolute fluorescence quantum yield of Ad[10]CPP^+^ was determined to be only 0.95% (Fig. S34) and the singlet excited-state lifetime was measured to be 1.88 ns (Fig. S37). Based on these values, the nonradiative decay rate constant (*k*_nr_ = 5.3 × 10^8^ s^−1^) is significantly higher than the radiative decay rate constant (*k*_r_ = 5.1 × 10^6^ s^−1^), indicating that the excited-state energy of Ad[10]CPP^+^ is primarily dissipated through non-radiative pathways. To gain further insight into the nature of the emissive state, we examined the emission spectra of Ad[10]CPP^+^ in solvents of varying polarity (CCl_4_, CHCl_3_, C_2_H_2_Cl_4_, CH_2_Cl_2_, and C_2_H_4_Cl_2_). A small negative solvatochromic effect of approximately 20 nm was observed (Fig. S29), suggesting that the emissive state is likely a LE state with only minor ICT character.^67,68^ Several donor–acceptor cycloparaphenylene derivatives have been reported to exhibit low quantum yields due to significant spatial HOMO–LUMO separation that promotes non-radiative deactivation of the excited state.^34,35,69–77^

### Electrochemical properties

The electrochemical behavior of Ad[10]CPP^+^ was elucidated through cyclic voltammetry (CV) and differential pulse voltammetry (DPV), with DPAd^+^ serving as a comparative control (Fig. 3c, S41 and S42). The reference compound DPAd·BF_4_ revealed two distinct reduction waves at −0.49 V and −1.45 V (*vs.* Ag/AgCl), respectively.^78^ These waves correspond to two single-electron reduction processes, where DPAd·BF_4_ first undergoes a reversible one-electron reduction to a radical species, followed by an irreversible one-electron reduction to form an anionic intermediate, which then captures a proton to afford DPAd-H (Fig. S38a).^79–81^Ad[10]CPP·BF_4_ displays similar two single-electron reduction processes with two notable distinctions. Firstly, the redox waves were shifted to a more negative potential by approximately 400 mV, appearing at −1.01 V and −1.94 V, respectively. Secondly, the transition to the radical state exhibited a semi-reversible nature (Fig. S38b). The calculated radical state Ad[10]CPP˙ shows a geometry between the teardrop shape of Ad[10]CPP-H/Ad[10]CPP-OH and the close-to-circle shape of Ad[10]CPP^+^, namely, the ratio of the long axis and short axis of the radical state falls between them (Fig. S74). It is postulated that a geometrical change occurs upon the formation of the radical species, which, when attempting to revert the acridinium form, requires the molecular framework to overcome significant ring strain. In our subsequent efforts, we attempted to generate and characterize Ad[10]CPP˙, the radical state of the macrocycle. As previously noted, the acridinium form Ad[10]CPP^+^ is only stable under moderately acidic conditions, which posed a challenge for generating radicals through reduction with active metals or cobaltocene, as these reagents tend to react with the acid first.

To circumvent this issue, we tried an alternative strategy involving the oxidation of Ad[10]CPP-H to achieve the radical state by treating Ad[10]CPP-H with nitrosonium tetrafluoroborate (NOBF_4_), a well-known one-electron oxidizing agent. To our surprise, Ad[10]CPP-dimers were generated after quenching the solution with air, as indicated in the HR-MS spectra (Fig. S24). This result suggests that the generated radical species is highly prone to dimerization. We were able to isolate the dimers. The ^1^H NMR and ^1^H–^1^H COSY spectra (Fig. S52 and S53) indicated a mixture of two compounds in a ∼7 : 3 ratio with very similar structures, as they were not differentiated by diffusion constants in the DOSY spectrum (Fig. S55) or the analytical HPLC trace (Fig. S56). This also explains the lack of hyperfine splitting patterns in the EPR spectrum (Fig. S47a). Further oxidizing the isolated dimers produces a broad near-infrared absorption band in the UV-vis-NIR spectrum ranging from 1000 nm to 2000 nm, with *λ*_max_ at 1574 nm (Fig. S58), possibly coming from diradical dications of the dimers.^82^ A plausible mechanism for the formation of Ad[10]CPP-dimers is illustrated in Fig. S54. Oxidation of Ad[10]CPP-H generates a prochiral radical cation, which can dimerize by forming a new σ-bond at the *para* positions relative to the nitrogen atom. This process, accompanied by the loss of two protons, yields a *meso* dimer and a pair of racemic dimers. The inherent strain in Ad[10]CPP-H induces an electronic effect that governs a highly selective dimerization pathway, leading to the formation of giant double nanohoops.

### Host and guest interactions

With the structure of Ad[10]CPP^+^ well characterized, we proceeded to evaluate its host capabilities for binding guest molecules, focusing on C_60_ due to the expected similarity in cavity size between Ad[10]CPP^+^ and [10]CPP.^45^ Significant chemical shift changes in the ^1^H NMR spectra of Ad[10]CPP·TFA upon the addition of C_60_ provided clear evidence of strong supramolecular interactions between the two species (Fig. S60). The binding constant of Ad[10]CPP·TFA and C_60_ was determined to be (2.18 ± 0.27) × 10^4^ M^−1^ (Fig. S61) by NMR titration experiments, which is smaller than that of [10]CPP, (2.79 ± 0.03) × 10^6^ M^−1^, for C_60_ characterized by fluorescence-quenching experiments.^44^

**Fig. 4:**
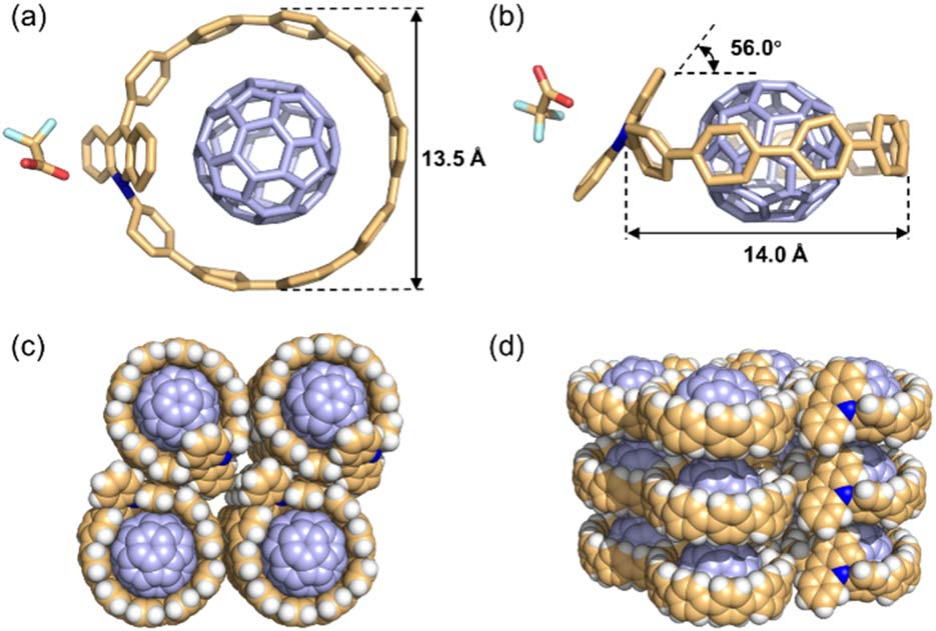
(a) Top view and (b) side view of the X-ray solid-state structure of C_60_⊂Ad[10]CPP·TFA. Hydrogen atoms are omitted for clarity. (c) Top view and (d) side view of crystal packing showing columnar stacks of C_60_⊂Ad[10]CPP·TFA; solvent molecules and counterions are omitted for clarity. Color code: C, tan/lavender; N, blue; O, red; F, pale cyan. For crystallographic details and CCDC numbers see the SI.

After multiple attempts, we successfully obtained single crystals of the host–guest complex C_60_⊂Ad[10]CPP·TFA suitable for X-ray diffraction analysis. The crystal structure unambiguously confirms a 1 : 1 host–guest complex, with the C_60_ guest residing within the cavity of Ad[10]CPP·TFA (Fig. 4). The cycloparaphenylene host exhibits a slightly elliptical shape, with a long and short axis of 14.0 Å and 13.6 Å, respectively (Fig. 4a and b). Further structural analysis reveals more compact supramolecular interactions compared to C_60_⊂[10]CPP, as evidenced by the shorter average distance (3.21 Å) from the equatorial carbon atoms of C_60_ to the nearest phenylene plane of the host, relative to the 3.44 Å observed in the latter^45^ (Fig. S71). The torsion angles between the acridinium and its adjacent phenylene moieties are 53.8° and 75.8°, respectively, markedly larger than those between other phenylene rings (8.3°–29.0°), which can be attributed to steric repulsion from the hydrogen atoms at the acridinium–phenylene junctions (Fig. S72). Additionally, the acridinium fragment forms a dihedral angle of 57.2° with the macrocyclic plane (Fig. 4b). Collectively, we propose that this strained, elliptical structure imposes a certain compression upon C_60_ encapsulation, which diminishes the optimal host–guest complementarity achieved in the nearly circular [10]CPP complex, thereby accounting for the lower binding constant.

In order to assess the stabilizing effect of C_60_ encapsulation on the Ad[10]CPP^+^ host, we conducted systematic titration experiments using TFA. The protonation processes of Ad[10]CPP-OH by TFA were monitored *via* characteristic NMR signals, both in the presence and absence of C_60_ (Fig. S62 and S63). A change in solvent (CH_2_Cl_2_ to C_2_Cl_4_D_2_) lowered the concentration of TFA required for the complete conversion of Ad[10]CPP-OH to Ad[10]CPP^+^. At a TFA concentration of 1.0 × 10^−4^ M, the conversion of free Ad[10]CPP-OH was negligible, whereas partial conversion was achieved when C_60_ was present. This stabilization effect was further confirmed at a TFA concentration of 2.0 × 10^−4^ M, where Ad[10]CPP-OH alone underwent incomplete conversion, but full conversion was achieved in the presence of excess C_60_ (Fig. S64 and S65). These results demonstrate that host–guest complexation stabilizes the vulnerable structure of the host, thereby reducing the acid requirement for stabilizing the cationic host structure. This provides direct evidence that C_60_ substantially reinforces the molecular scaffold *via* π–π interactions. The encapsulation improves its stability against atmospheric moisture compared to the empty host, enabling the successful growth of single crystals suitable for X-ray diffraction, ultimately allowing us to resolve the structure of the C_60_⊂Ad[10]CPP·TFA complex.

### Stimulus responsive system

**Fig. 5:**
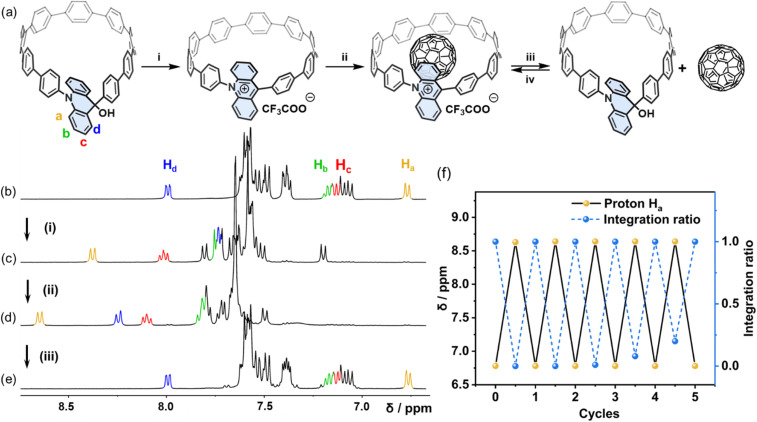
(a) Schematic representation of acid–base responsive capture and release of C_60_ (0–4.09 mM) by macrocycle Ad[10]CPP·TFA (0.53 mM) in CD_2_Cl_2_. Conditions and reagents: (i) TFA, CD_2_Cl_2_, (ii) C_60_, CD_2_Cl_2_, ultrasound, and (iii) TEA, CD_2_Cl_2_, (iv) TFA, CD_2_Cl_2_, ultrasound. ^1^H NMR spectra (400 MHz, CD_2_Cl_2_, 298 K) of (b) Ad[10]CPP-OH and with sequential addition of (c) 5 eq. of TFA, (d) 1.5 eq. of C_60_, and (e) 5 eq. of TEA. (f) Capture and release of C_60_ cycles of Ad[10]CPP·TFA under acid/neutral conditions, monitored using the ^1^H NMR spectra.

With the binding affinity between Ad[10]CPP·TFA and C_60_ measured and the structure of host–guest complex characterized, we then explored the acid–base stimuli responsive properties of the host–guest complex (Fig. 5a). Gradually adding five equivalents of TFA to an NMR tube containing a CD_2_Cl_2_ solution of Ad[10]CPP-OH (Fig. 5b) caused a significant chemical shift change in the proton associated with the acridinium moiety. Specifically, *H*_a_ shifted from 6.78 ppm to 8.37 ppm, indicating complete transformation into Ad[10]CPP·TFA (Fig. 5c). Subsequently, adding 1.5 equivalents of C_60_ to the same NMR tube caused *H*_a_ to shift further downfield to 8.64 ppm, attributed to the formation of the host–guest complex C_60_⊂Ad[10]CPP·TFA (Fig. 5d). Adding five equivalents of triethylamine (TEA) to the solution caused the *H*_a_ signal to revert to 6.78 ppm, indicating that the macrocycle had completely transformed back into Ad[10]CPP-OH (Fig. 5e). Since the cavity of Ad[10]CPP-OH is not suitable for C_60_ (Fig. S59), the guest molecule is then released into the solution. The acid–base switch process is reversible for at least 5 cycles, as monitored by ^1^H NMR spectroscopy (Fig. 5f and S67).

## Conclusions

In summary, we successfully synthesized and characterized an acridinium-functionalized cycloparaphenylene derivative, Ad[10]CPP^+^. Structural characterization indicates that Ad[10]CPP^+^ adopts a nearly circular configuration similar to [10]CPP, with increased torsion angles in the acridinium moiety due to steric hindrance. Electrochemical studies reveal two well-separated reduction peaks for Ad[10]CPP^+^, suggesting its potential to form a radical species, Ad[10]CPP˙. However, attempts to generate Ad[10]CPP˙ through oxidation resulted in its dimerization, yielding giant double nanohoops. Ad[10]CPP^+^ also demonstrates strong host–guest interactions with electron-rich fullerenes, as evidenced by NMR spectroscopy and single-crystal X-ray analysis. Furthermore, Ad[10]CPP^+^ can undergo reversible structural transformations in response to chemical stimuli, enabling controlled capture and release of fullerene guests under alternating acidic and basic conditions for multiple cycles. The acid–base triggered guest capture and release of Ad[10]CPP^+^ represents a valuable addition to the cycloparaphenylene family. Its key advantage lies in the highly confined cavity and switchable cationic character. Our current efforts are focused on leveraging multi-acridinium motifs to develop advanced functional systems for applications in sensing wider guests.

## Author contributions

C. C. conceived and supervised the project. J. M. performed most of the synthesis and characterization experiments. J. Z. and P. Y. conducted the calculations. Y. H., J. J., X. Z. and H. Z. helped with synthesis of starting materials. K. L., D. Z., and P. L. helped with characterization. All authors contributed to manuscript writing.

## Conflicts of interest

There are no conflicts to declare.

## Supplementary Material

SC-017-D6SC01132B-s001

SC-017-D6SC01132B-s002

## Data Availability

CCDC 2449558, 2449559 and 2449561 contain the supplementary crystallographic data for this paper.^83*a*–*c*^ Supplementary information (SI): additional experimental details, materials and methods, ^1^H and ^13^C NMR, (^1^H, ^1^H)-COSY NMR, MS, and UV-vis-IR spectra, EPR spectra, crystallographic data, and computational details (PDF). See DOI: https://doi.org/10.1039/d6sc01132b.
