# Supplementary material for: A dual-responsive cationic acridinium nanohoop: redox activity and acid/base-controlled reversible guest capture and release
Source: Chem Sci. 2026 Apr 13;17(21):10744–54. doi: 10.1039/d6sc01132b (PMC13098699; doi:10.1039/d6sc01132b)
Supplement: SC-017-D6SC01132B-s001 [file SC-017-D6SC01132B-s001.pdf]

Supplementary Information (SI) for Chemical Science.
This journal is © The Royal Society of Chemistry 2026

A Dual-Responsive Cationic Acridinium Nanohoop: Redox Activity and Acid/Base-Controlled Reversible Guest Capture and Release

Supporting information

Jiao Ming, Jin Zhang, Yan Hu, Kai Lan, Dongmei Zhang, Ping Li, Jiarong Miao, Jiyong Jiang, Xiaobo Zhang, Hanchi Zhong, Peiyuan Yu, Chuyang Cheng

1.General Experimental Details: 1
2. Synthesis and Characterization 2
3. NMR and Mass spectra 7
4. Photophysical properties for Ad[10]CPP (UV, FL, PLQY and Fluorescence lifetime) 18
5. Electrochemical Characterization (CV and DPV) 26
6. Chemical Oxidation Experiments (UV-vis-IR, EPR) 30
7. Complexation of Ad[10]CPP·TFA with C_{60} 38
8. pH-stimulate responsive properties of Ad[10]CPP·TFA 43
9. X-ray crystallographic data 44
10. Theoretical Calculations 52
11. References 85

1.General Experimental Details:

All reagents were purchased from commercial source and used without further purification. Compound 2[1], 4[2], and 5[3] were prepared according to literature method. Anhydrous THF and dichloromethane (DCM) was used by treatment with a solvent purification system. Column chromatography was performed with silica gel (200-300 mesh). Proton (^{1}H) NMR and carbon (^{13}C) NMR spectra were recorded on AVANCE NEO 400NMR spectrometer with tetramethylsilane (TMS) as the internal standard. Chemical shifts were assigned in ppm relative to residue protons ($CHCl_3$: 7.26 for ^{1}H, 77.16 for ^{13}C; DMSO:2.50 for ^{1}H, 39.52 for ^{13}C). The following abbreviations were used for multiplicities: s = singlet, d = doublet, t = triplet, m = multiplet. Matrix Assisted Laser Desorption Ionization (MALDI) mass spectra were performed on AXIMA Performance. UV-Vis absorption spectra were recorded on SHIMADZU UV-2600i spectrophotometer in DCM. Photoluminescence (PL) and excitation spectra was obtained by FL970 Fluorescence Spectrometer. The absolute singlet quantum yield, the lifetimes of the singlet excited states of liquid samples were measured using an Fluoromax-3, HORIBA TEMPRO-01 or Fluoroolg-3 spectrometer. UV-Vis-NIR spectra were recorded on HITACHI U-4100 spectrophotometer in DCM. The HPLC chromatogram was obtained by SHIMADZU LC-20AR spectrophotometer in DCM. The electrochemical measurements were carried out in anhydrous CH_3CN containing n-Bu_4NBF_4 as supporting electrolyte at a scan rate of 100 mV/s at room temperature under argon atmosphere by CHI 660e electrochemical analyzer. A three-electrode system was used, consisting of a glassy carbon working

electrode (3 mm diameter, geometric area: 0.07 cm²), an Ag/AgCl reference electrode, and a Pt wire counter electrode. The working electrode surface was polished routinely with a 0.05 μm alumina-water slurry on a felt surface immediately before use. The EPR spectra were obtained on a Bruker EMX plus X-band EPR spectrometer, where all solvents used were thoroughly deoxygenated via three freeze-pump-thaw cycles prior to analysis. The EPR samples were prepared and sealed in a glovebox prior to measurement.

2. Synthesis and Characterization

Synthesis of Compound 2[1]:

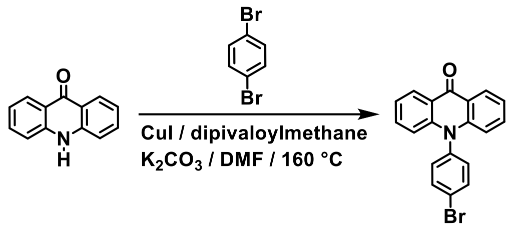

1 **2**

1,4-dibromobenzene (4.79 g, 20.5 mmol), acridin-9(10H)-one (4.0 g, 20.5 mmol), K_2CO_3 (3.11g, 22.5 mmol), CuI (0.39 g, 2.05 mmol), 2,2,6,6-tetramethyl-3,5-heptanedione (0.76 g, 4.12 mmol) were dissolved in anhydrous DMF (6 mL/1 mmol of acridone) into a two-necked round flask. The mixture was degassed and refluxed under nitrogen atmosphere for 24 h. After cooling to room temperature, the reaction mixture was quenched with H_2O and extracted with ethyl acetate (3×10 mL). The combined organic layers were collected, dried over sodium sulfate, and evaporated under vacuum. Then, the crude product was purified by flash chromatography on silica gel (petroleum ether/ethyl acetate = 9/1) to afford **2** in 58% yield as a white solid. ^{1}H NMR (400 MHz, DMSO-*d*$_6$) δ 8.38 (dd, *J* = 8.0, 1.7 Hz, 2H), 7.99 (d, *J* = 8.6 Hz, 2H), 7.66 (ddd, *J* = 8.7, 7.0, 1.7 Hz, 2H), 7.54 (d, *J* = 8.5 Hz, 2H), 7.35 (ddd, *J* = 8.0, 7.0, 1.0 Hz, 2H), 6.77 (d, *J* = 8.6 Hz, 2H).

Synthesis of Compound 3[4]:

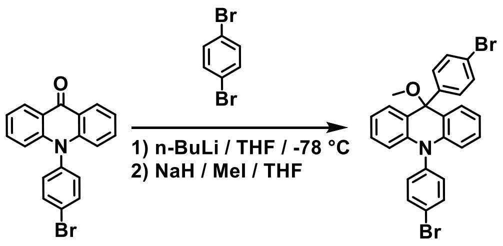

2 **3**

1,4-dibromobenzene (9.35 g, 40 mmol) and dry THF (100 mL) were added to a two-necked 500 mL glass flask filled with N_2. *n*-butyllithium (16 mL, 40.0 mmol) was added to the flask at -78 ºC. After mixing the solution at -78 ºC for 30 min, the mixture was cannulated into a degassed THF solution (100 mL) of **2** (3.5 g, 10 mmol) at -78 ºC. The mixture was stirred at -78 ºC for 3 h and

was subsequently warmed to room temperature. The mixture was quenched with water, extracted with CH_2Cl_2, dried over Na_2SO_4, and concentrated under reduced pressure. NaH (0.35 g, 60% mineral oil, 8.68 mmol) and MeI (0.54 mL, 8.68 mmol) was suspended in 40 mL anhydrous THF. After 16 h stirring, the excess sodium hydride was quenched by the addition of water. After layer separation, the aqueous layer was extracted with CH_2Cl_2 three times. The combined organic layers were washed with brine, dried over Na_2SO_4 and the organic solvents were removed under reduced pressure. The obtained solid was further washed with petroleum ether and acetone to afford **3** in 55% yield as a white solid. ^{1}H NMR (400 MHz, $CDCl_3$) δ 7.80 (d, J = 8.5 Hz, 2H), 7.40 (d, J = 8.7 Hz,2H), 7.35 (d, J = 8.6 Hz, 2H), 7.24 (d, J = 8.5 Hz, 2H), 7.20 (dd, J = 7.8, 1.6 Hz, 2H), 7.05 (ddd, J = 8.5, 7.1, 1.6 Hz, 2H), 6.87 (td, J = 7.5, 1.1 Hz, 2H), 6.32 (dd, J = 8.4, 1.1 Hz, 2H), 3.00 (s, 3H). ^{13}C NMR (101 MHz, $CDCl_3$) δ 149.7, 140.8, 139.7, 134.5, 132.9, 130.8, 130.1, 128.3, 122.6, 122.1, 121.0, 120.3, 114.1, 77.9, 50.8. HRMS(ESI): [M-MeO]$^+$ calcd for $C_{25}H_{16}Br_2N^+$ 487.9645, found 487.9645.

Synthesis of Compound 6:

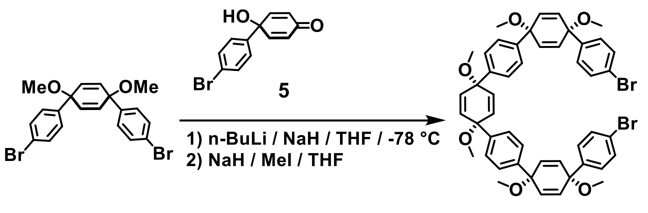

To a 100 mL round-bottom flask containing a magnetic stirring bar were added **4** (11.0 g, 24.6 mmol), and dry THF (100 mL). A solution of n-butyllithium in hexane (19.6 mL, 2.5 M, 49.1mmol) was added at -78 °C. After stirring the mixture at -78 °C for 30 min, the mixture was added to the solution of **5** (6.51 g, 24.6 mmol) and NaH (1.47 g, 60% mineral oil, 36.8 mmol) in THF (100 mL), and the resultant mixture was further stirred at -78 °C for 2 h. After warmed up to room temperature, the mixture was quenched with water, extracted with CH_2Cl_2, dried over Na_2SO_4, and concentrated under reduced pressure. NaH (3.93 g, 60% mineral oil, 98.2 mmol) and MeI (6.12 mL, 98.2 mmol) was suspended in 100 mL anhydrous THF. After 16 h stirring, the excess sodium hydride was quenched by the addition of water. After layer separation, the aqueous layer was extracted with CH_2Cl_2 three times. The combined organic layers were washed with brine, dried over Na_2SO_4 and the organic solvents were removed under reduced pressure. The residue was purified by silica gel column chromatography (EtOAc/petroleum ether = 1/10) to afford **6** in 39% as a white solid. ^{1}H NMR (400 MHz, $CDCl_3$) δ 7.42 (d, J = 8.6 Hz, 4H), 7.36 (d, J = 8.7 Hz, 4H), 7.32 (d, J = 8.7 Hz, 4H), 7.26 (d, J = 8.6 Hz, 4H), 6.11 (d, J = 10.3 Hz, 4H), 6.08 (s, 4H), 6.04 (d, J = 10.3 Hz, 4H), 3.42 (s, 6H), 3.42 (s, 6H), 3.41 (s, 6H). ^{13}C NMR (101 MHz, $CDCl_3$) δ 143.1, 142.7, 142.7, 133.9, 133.5, 133.1, 131.6, 128.0, 126.2, 126.1, 121.7, 74.7, 74.7, 74.6, 52.2, 52.1. HRMS(ESI): [M+Na]$^+$ calcd for $C_{48}H_{46}Br_2O_6Na$ 901.1544, found 901.1529.

Synthesis of Compound 7:

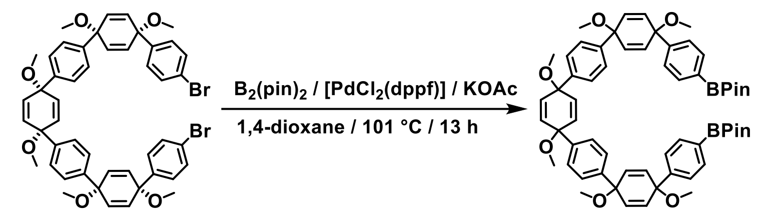

6 **7**

A 100-mL round-bottom glass flask containing a magnetic stirring bar was flame-dried under vacuum and filled with argon after cooling to room temperature. To this flask were added **6** (1.0 g, 1.14 mmol), [$PdCl_2$(dppf)] (50.0 mg, 6% mmol), bis(pinacolate)diboron (2.0 g, 8.90 mmol), KOAc (0.9 g, 8.90 mmol), and dry 1,4-dioxane (20 mL). After stirring at 101 °C for 13 h, saturated NH_4Cl aqueous solution was added to the reaction mixture. The mixture was then extracted with dichloromethane, and the combined organic phase was dried over Na_2SO_4 and concentrated under reduced pressure. The crude product was subjected to silica gel column chromatography (EtOAc/petroleum ether = 1/10) to afford **7** in 55% yield as a white solid. ^{1}H NMR (400 MHz, Chloroform-*d*) δ7.75 (d, J = 8.3 Hz, 4H), 7.40 (d, J = 8.4 Hz, 4H), 7.34 (s, 8H), 6.08 (m, 12H), 3.42 (s, 12H), 3.41 (s, 6H), 1.33 (s, 24H). ^{13}C NMR (101 MHz, $CDCl_3$) δ 146.6, 142.9, 142.8, 135.1, 133.5, 133.4, 133.3, 126.2, 125.4, 83.9, 75.0, 74.8, 74.7, 52.1, 25.2, 25.0. HRMS(ESI): $[M+Na]^+$ calcd for $C_{60}H_{70}B_2O_{10}Na$ 995.5050, found 995.5050.

Synthesis of Compound Ad[10]CPP-H[5]:

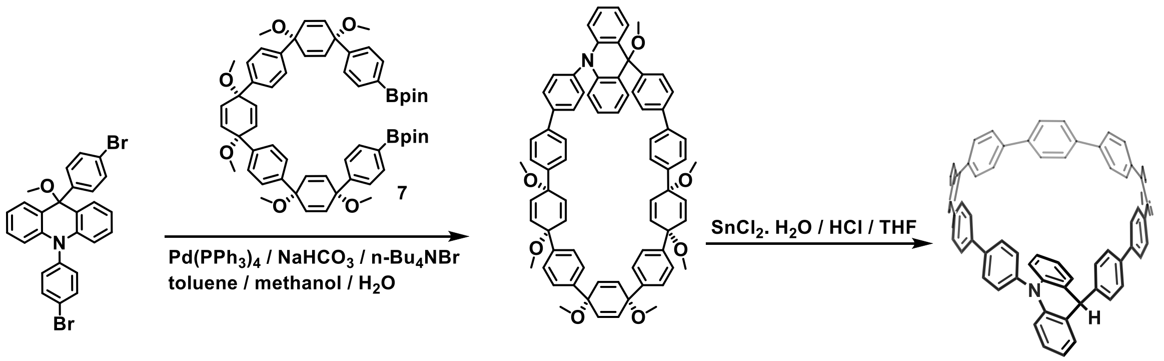

3 **8** **Ad[10]CPP-H**

Dibromide **3** (145 mg, 0.28 mmol), diboronate **7** (270 mg, 0.28 mmol), *n*-Bu_4NBr (36 mg, 0.056 mmol), Pd $(PPh_3)_4$ (64 mg, 0.110 mmol) and $NaHCO_3$ (233 mg, 2.8 mmol) were charged to a dried flask, which was then purged with argon. Degassed toluene (50 mL), methanol (6 mL), and water 3 mL (to make base 1 M) were added. The mixture was heated to 90 °C and stirred for 24 h. The reaction was cooled to room temperature and extracted with CH_2Cl_2. The organic layer was washed with brine and dried over sodium sulfate. The organic layer was concentrated down under reduced pressure to a crude solid **8**.

Concentrated aqueous HCl (0.65 mL, 7.8 mmol) was added to a solution of $SnCl_2 \cdot 2H_2O$ (0.88 g, 3.9 mmol) in THF (30 mL) at room temperature and the resulting solution was stirred at same

temperature for 30 min under nitrogen atmosphere. The crude solid **8** was dissolved in THF (20 mL) and then added to the resulting mixture at room temperature. The mixture was stirred at same temperature for 12 h. To the resulting mixture, 10% aqueous NaOH solution was added, and extracted with CH_2Cl_2. The combined organic layer was washed with brine, dried over Na_2SO_4, filtered and concentrated under reduced pressure. The crude product was subjected to silica gel column chromatography (CH_2Cl_2/petroleum ether =1/6) to afford **Ad[10]CPP-H** as a yellow solid (32 mg, 13% for over two steps). ^{1}H NMR (400MHz, $CDCl_3$) δ 7.61-7.55 (m, 14H), 7.55-7.48 (m, 8H), 7.46 (d, *J* = 7.2 Hz, 4H), 7.43 (d, *J* = 8.6 Hz, 2H), 7.37 (d, *J* = 6.3 Hz, 4H), 7.35 (d, *J* = 6.1 Hz, 2H), 7.15 (td, *J* = 7.7, 1.6 Hz, 2H), 7.10 (d, *J* = 8.4 Hz, 4H), 7.06 (td, *J* = 7.4, 1.2 Hz, 2H), 6.96 (d, *J* = 8.3 Hz, 2H), 6.84 (dd, *J* = 8.1, 1.2 Hz, 2H), 5.32 (s, 1H). ^{13}C NMR (101 MHz, $CDCl_3$) δ 144.1, 142.5, 141.5, 140.9, 139.9, 139.6, 139.0, 138.9, 138.8, 138.4, 138.3, 138.3, 138.2, 138.2, 138.0, 137.8, 137.6, 130.5, 129.3, 128.8, 128.3, 127.9, 127.8, 127.8, 127.6, 127.6, 127.6, 127.6, 127.5, 127.5, 127.4, 127.3, 127.2, 127.1, 126.9, 121.7, 116.5, 47.2. HRMS (MALDI): $[M]^+$ calcd for 863.3552, found 863.3533 (matrix: DCTB, internal standard: PPG, cationization agent: KI, linear mode).

Synthesis of Compound Ad[10]CPP-OH:

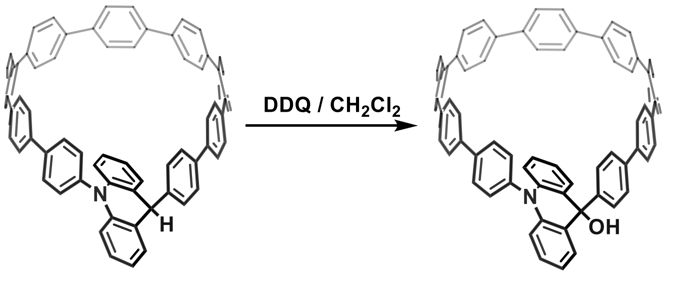

Ad[10]CPP-H **Ad[10]CPP-OH**

A solution of **Ad[10]CPP-H** (20.0 mg, 23.1 μmol) in CH_2Cl_2 (10.0 mL) was treated at room temperature with an excess of 2,3-Dichloro-5,6-Dicyanobenzoquinone **(**DDQ) (20.1mg, 88.5 μmol) and was stirred for 12 h. The mixture was quenched with a saturated solution of $Na_2S_2O_3$. The mixture was extracted by CH_2Cl_2. The organic layer was dried over Na_2SO_4 and concentrated in vacuo. The residue purified by chromatography over silica gel with CH_2Cl_2 to provide **Ad[10]CPP-OH** (11.0 mg, 54 %). ^{1}H NMR (400 MHz, 298 K, $CDCl_3$): δ (ppm) 8.02 (dd, *J* = 7.4, 1.8 Hz, 2H), 7.63-7.47 (m, 23H), 7.47-7.41 (m, 4H), 7.39-7.34 (m, 6H), 7.19 (td, *J* = 7.6, 1.9 Hz, 2H), 7.15 (td, *J* = 7.3, 1.5 Hz, 2H), 7.06 (dd, *J* = 8.4, 4.2 Hz, 4H), 6.80 (dd, *J* = 7.8, 1.5 Hz, 2H), 2.86 (s, 1H). ^{13}C NMR (101 MHz, $CDCl_3$) δ (ppm) 145.5, 141.3, 140.9, 140.7, 139.9, 139.7, 139.2, 138.9, 138.9, 138.8, 138.3, 138.2, 138.1, 138.1, 138.1, 137.9, 137.7, 137.4, 130.8, 129.7, 129.4, 128.2, 127.8, 127.8, 127.7, 127.7, 127.6, 127.5, 127.5, 127.5, 127.5, 127.4, 127.3, 127.2, 127.0, 127.0, 126.8, 124.7, 121.6, 115.7, 73.6. HRMS (MALDI): $[M]^+$ calcd for $C_{67}H_{45}NO$ 879.3501, found 879.3464 (matrix: DCTB, internal standard: PPG, cationization agent: KI, linear mode).

Synthesis of Compound Ad[10]CPP·BF_4

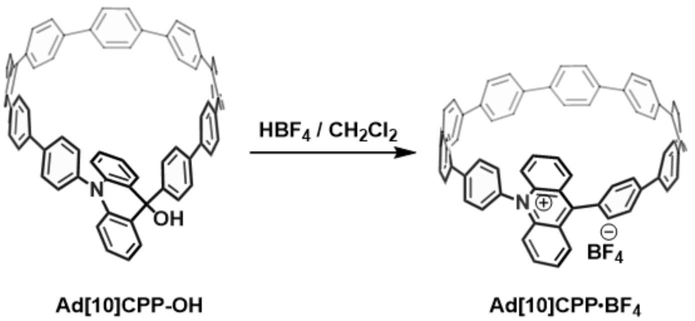

0.3 mL HBF_4 (40%) was added to a solution of **Ad[10]CPP-OH** (5.0 mg) in CH_2Cl_2 (5.0 mL) at room temperature and the resulting solution was stirred at the same temperature for 3 h. The mixture was concentrated under reduced pressure to afford **Ad[10]CPP•BF$_4$** as a dark purple solid.(**Basis for quantitative reaction in Figure S14).** ^{1}H NMR (400MHz, $CDCl_3$) δ (ppm) 8.30 (dd, J = 8.7, 1.3 Hz, 2H), 7.99 (ddd, J = 9.0, 6.9, 1.4 Hz, 2H), 7.79 (d, J = 8.6 Hz, 2H), 7.74 (d, J = 9.0 Hz, 2H), 7.70 (m, 6H), 7.67-7.59 (m, 8H), 7.55 (m, 20H), 7.32 (d, J = 8.7 Hz, 2H). ^{13}C NMR (101 MHz, $CDCl_3$) δ (ppm) 165.0, 145.3, 144.4, 143.3, 139.4, 139.3, 138.7, 138.6, 138.5, 138.3, 138.2, 138.2, 138.0, 137.4, 137.1, 132.6, 132.1, 130.3, 129.7, 128.8, 128.5, 128.3, 128.2, 127.9, 127.6, 127.5, 127.5, 127.3, 127.3, 127.3, 127.2, 127.2, 126.5, 120.1. HRMS (MALDI): $[M]^+$ calcd for $C_{67}H_{44}N^+$ 862.3468, found 862.3437 (matrix: HABA, internal standard: PPG, reflectron mode).

3. NMR and Mass spectra

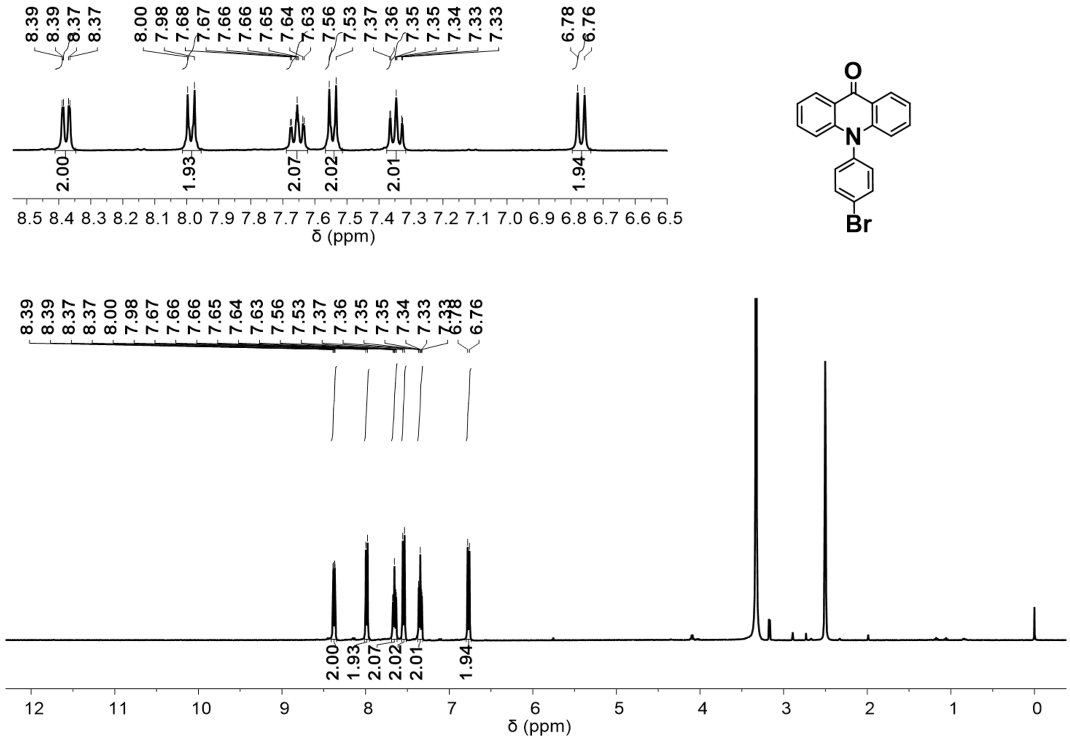

Figure S1. ^{1}H NMR spectra of **2** in DMSO-d_6 (298 K, 400 MHz).

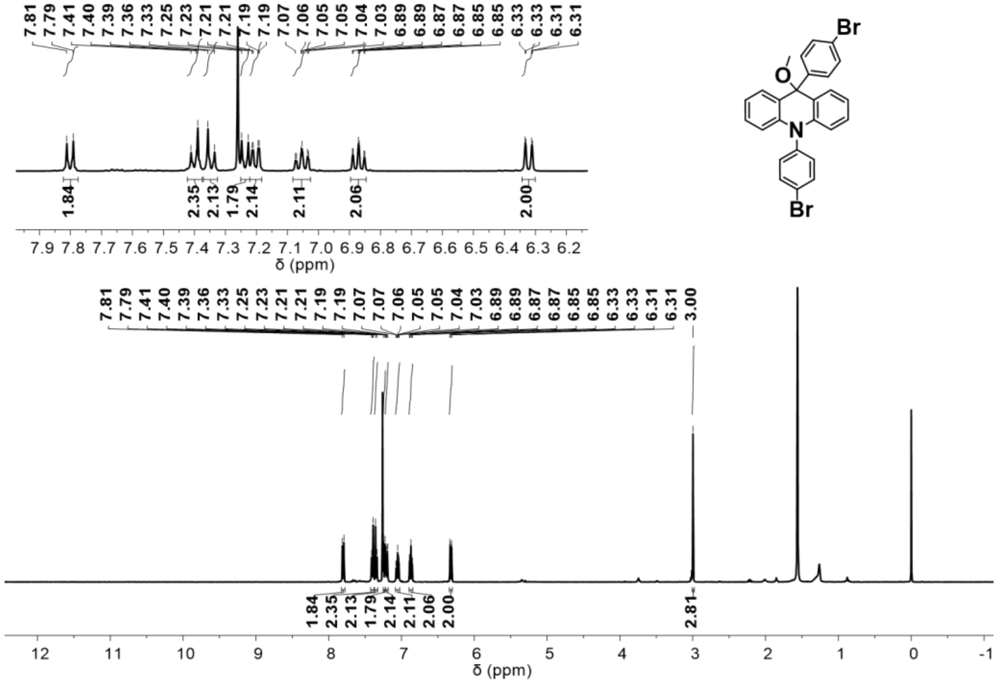

Figure S2. ^{1}H NMR spectra of **3** in $CDCl_3$ (298 K, 400 MHz).

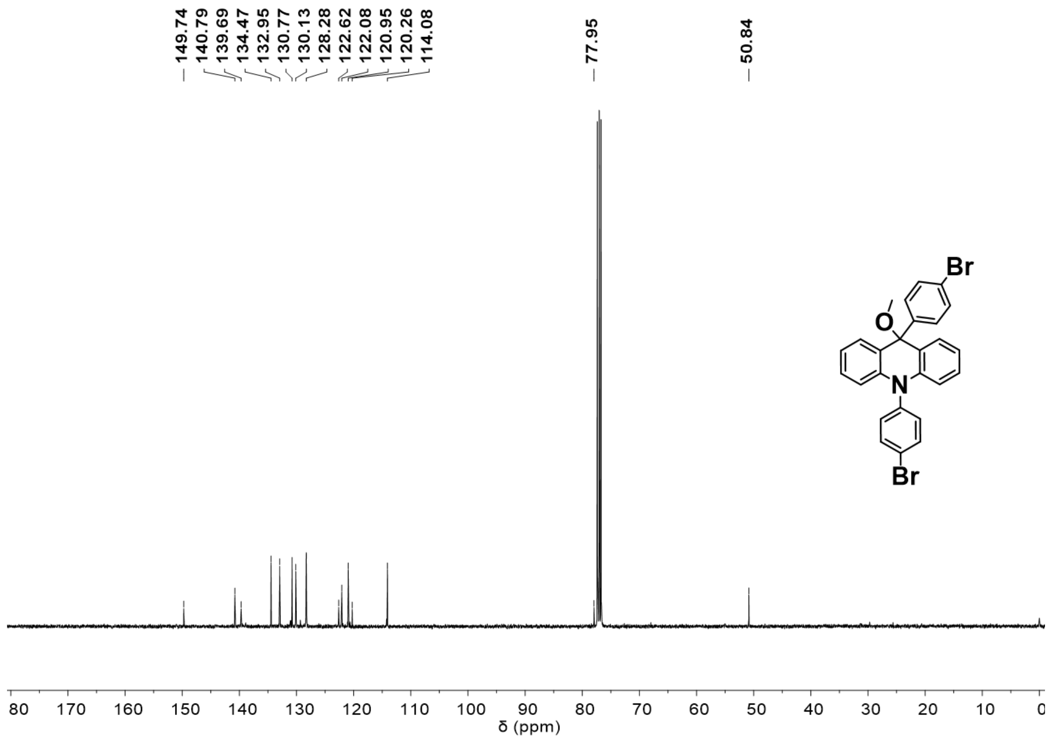

Figure S3. ^{13}C NMR spectra of **3** in $CDCl_3$ (298 K, 400 MHz).

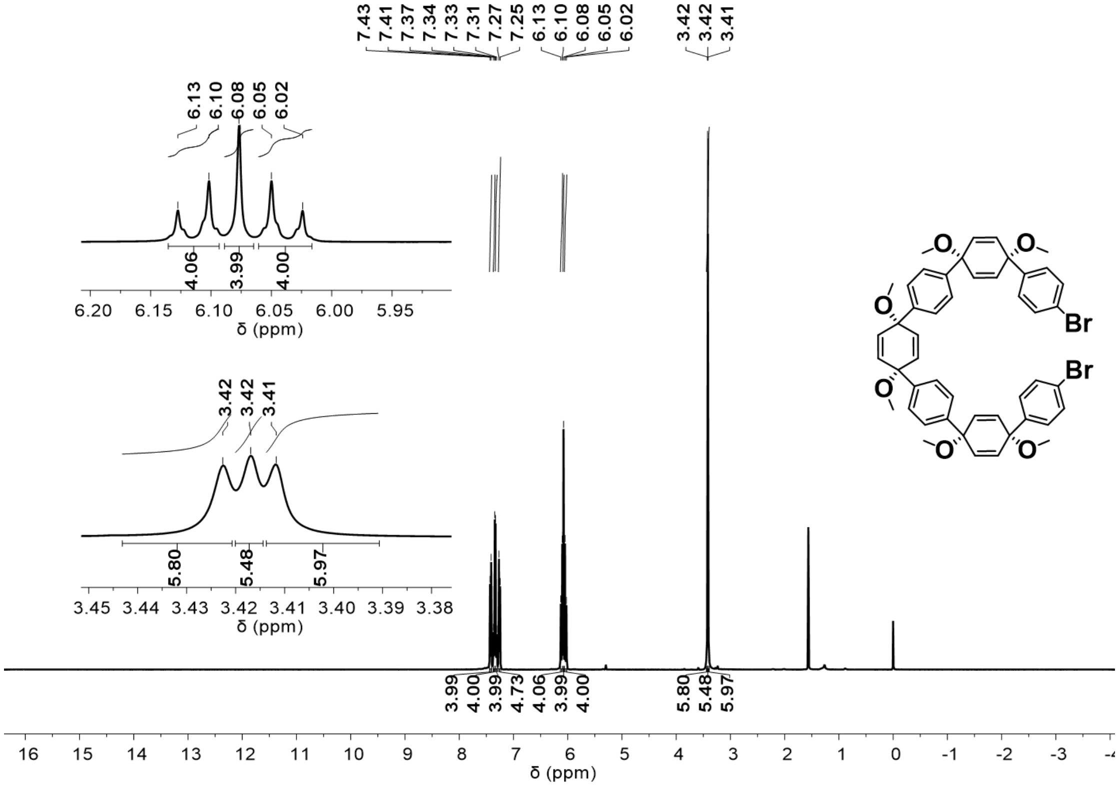

Figure S4. ^{1}H NMR spectra of **6** in $CDCl_3$ (298 K, 400 MHz).

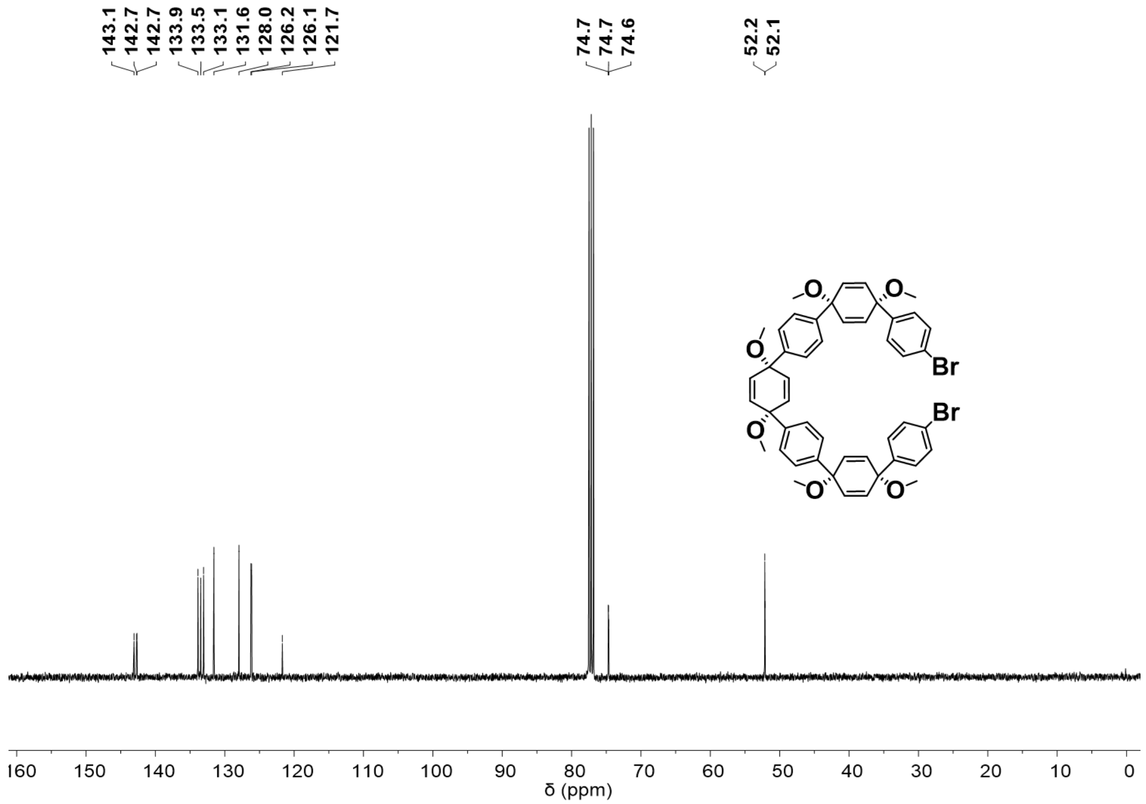

Figure S5. ^{13}C NMR spectra of **6** in $CDCl_3$ (298 K, 400 MHz).

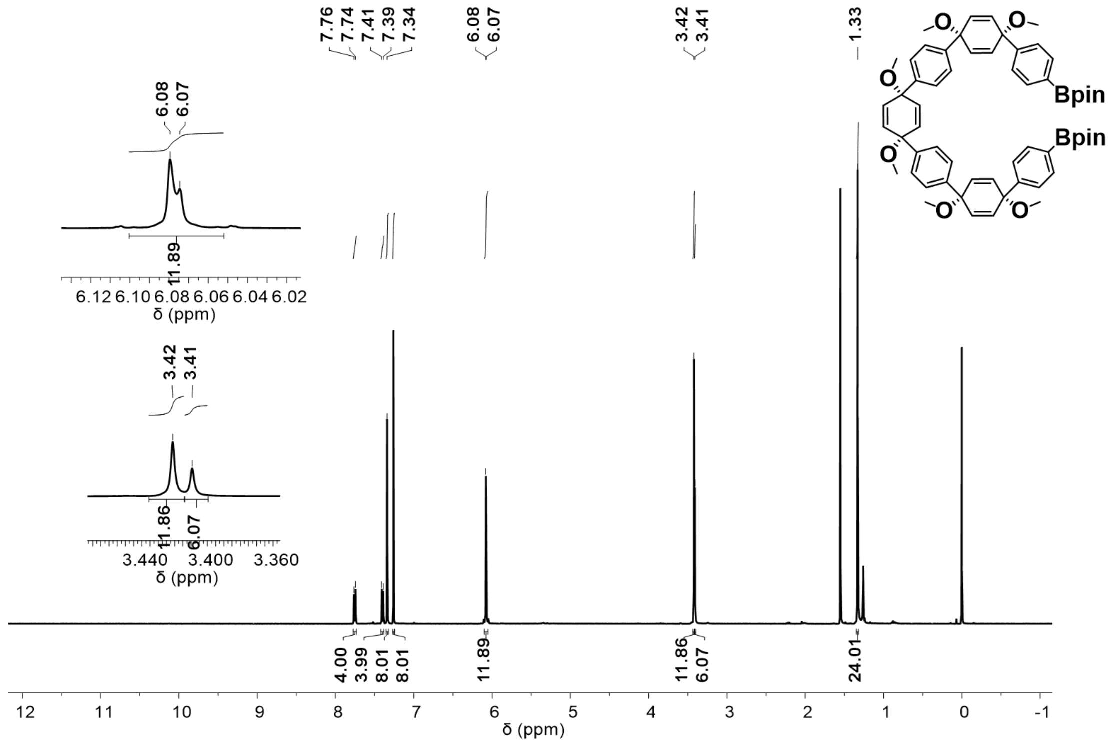

Figure S6. ^{1}H NMR spectra of **7** in $CDCl_3$ (298 K, 400 MHz).

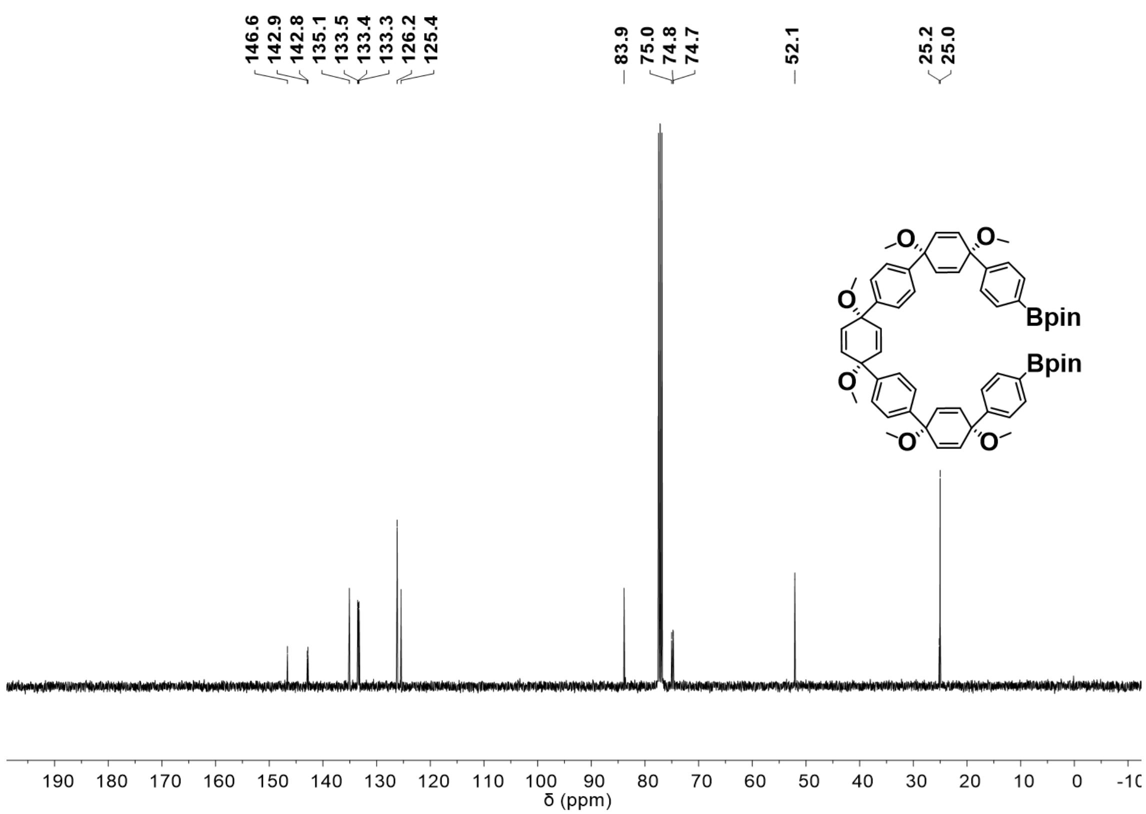

Figure S7. ^{13}C NMR spectra of **7** in $CDCl_3$ (298 K, 400 MHz).

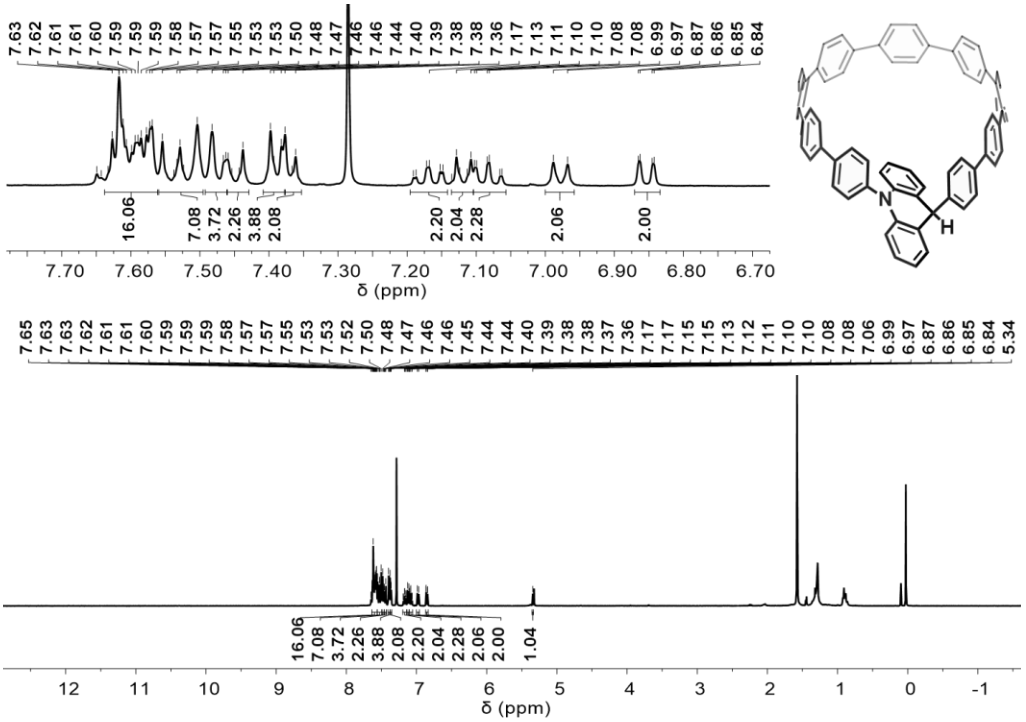

Figure S8. ^{1}H NMR spectra of **Ad[10]CPP-H** in $CDCl_3$ (298 K, 400 MHz).

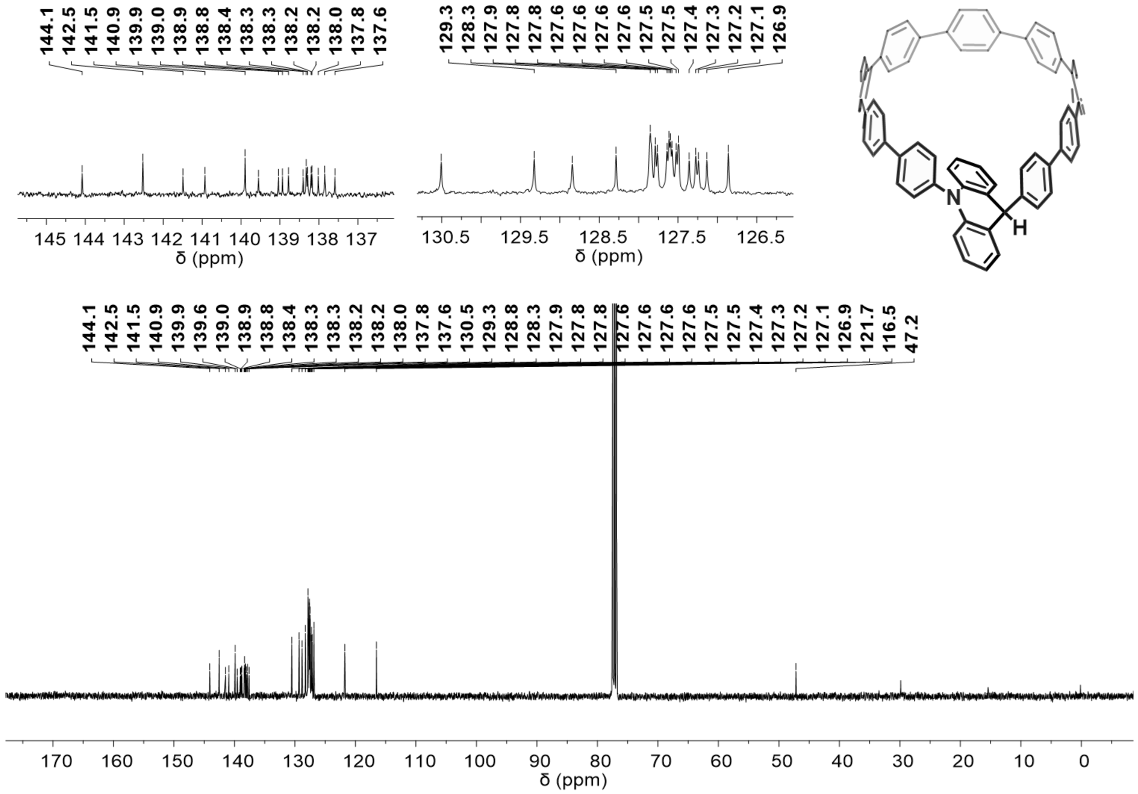

Figure S9. ^{13}C NMR spectra of **Ad[10]CPP-H** in $CDCl_3$ (298 K, 400 MHz).

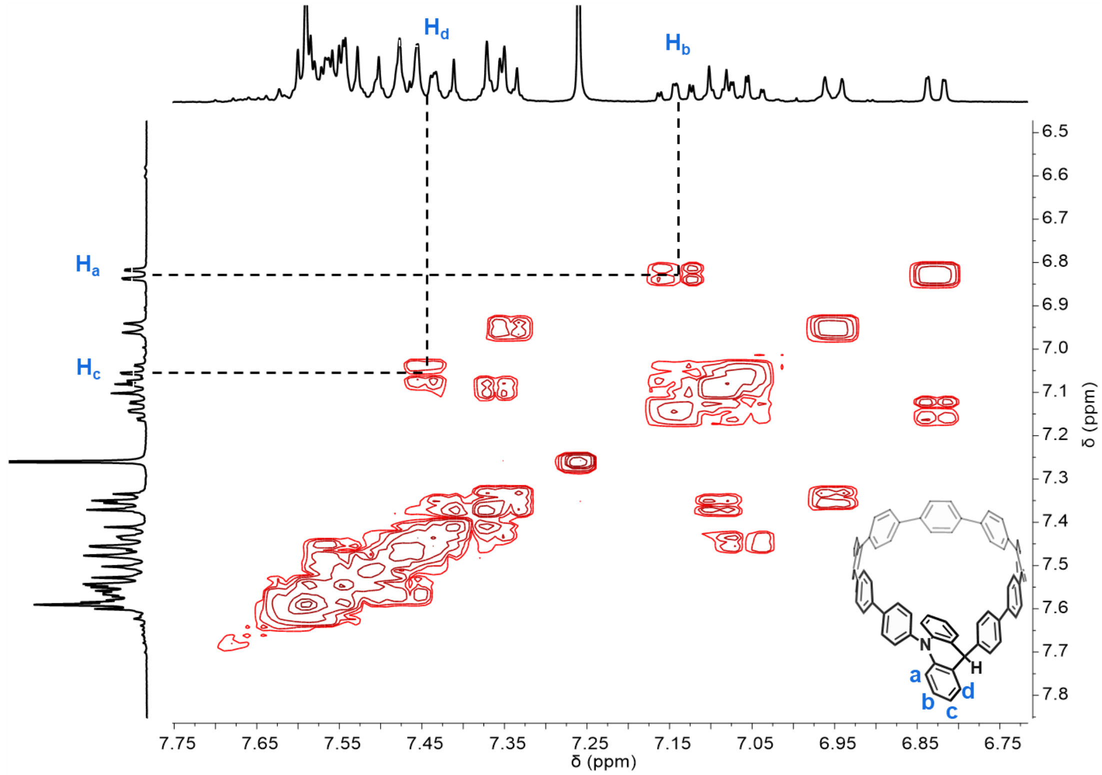

Figure S10. ^1H-^1H COSY spectra of **Ad[10]CPP-H** in $CDCl_3$ (298 K, 400 MHz).

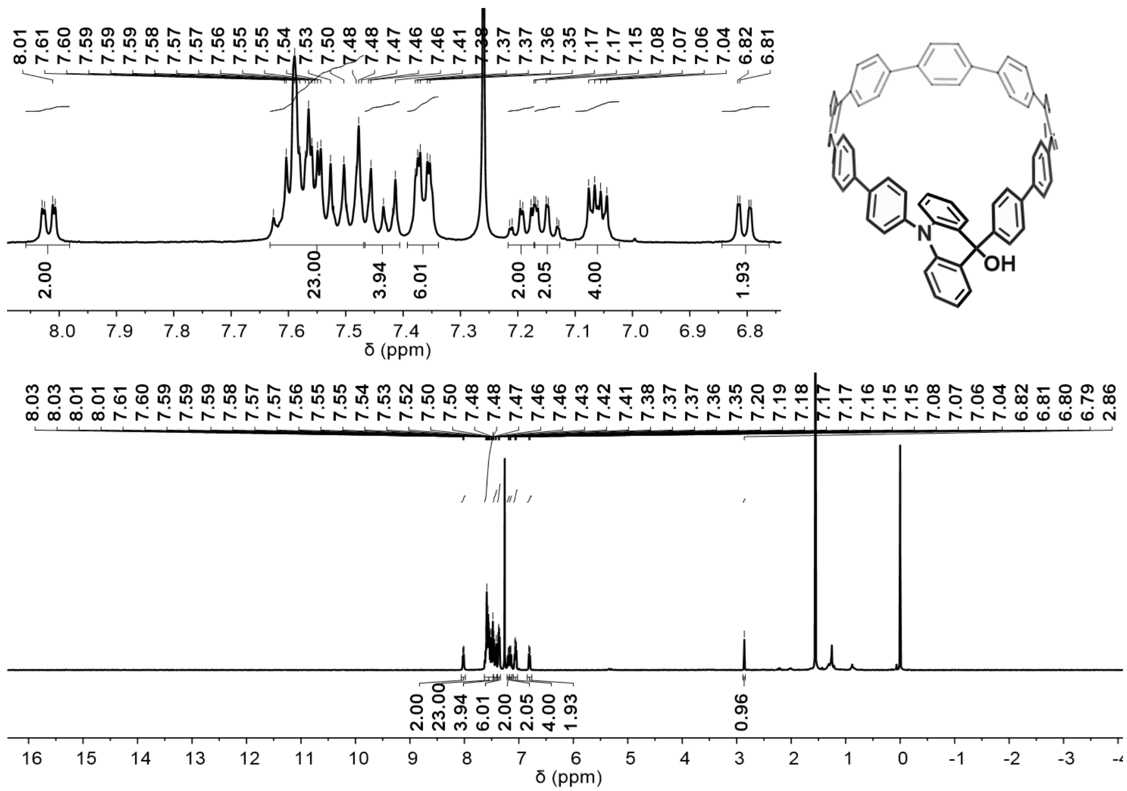

Figure S11. ^{1}H NMR spectra of **Ad[10]CPP-OH** in $CDCl_3$ (298 K, 400 MHz).

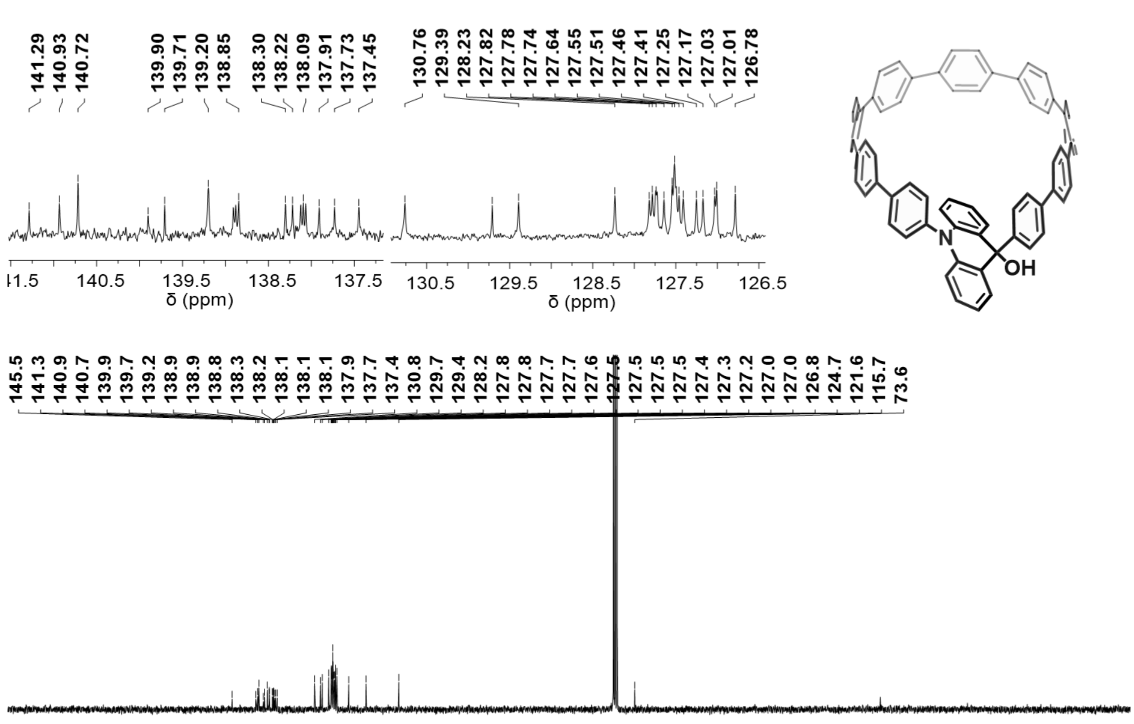

Figure S12. ^{13}C NMR spectra of **Ad[10]CPP-OH** in $CDCl_3$ (298 K, 101 MHz).

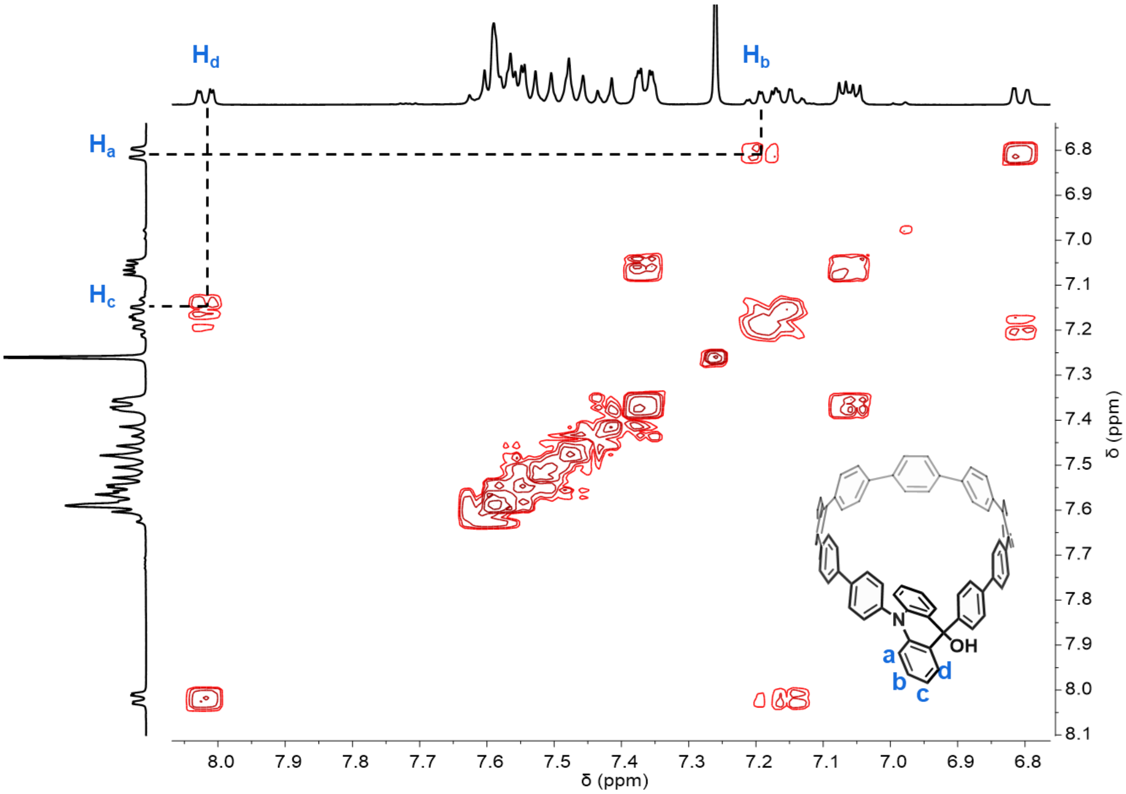

Figure S13. ^{1}H-^{1}H COSY spectra of **Ad[10]CPP-OH** in $CDCl_3$ (298 K, 400 MHz).

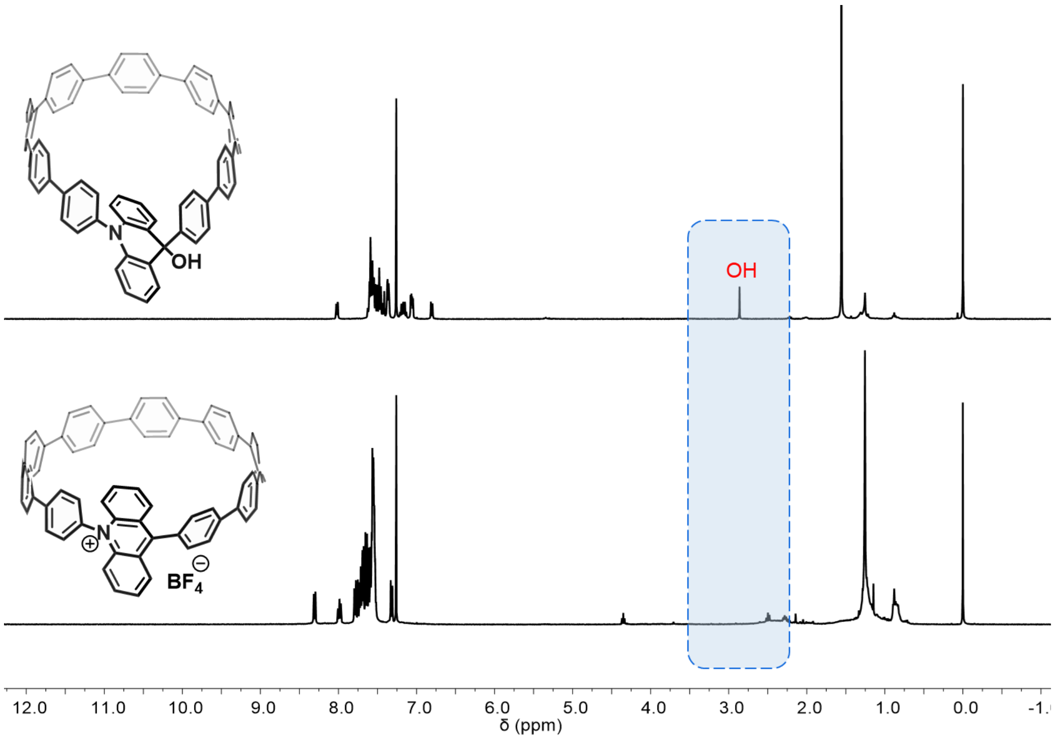

Figure S14. ^{1}H NMR spectra of **Ad[10]CPP-OH** before (top) and after (bottom) treatment with an excess of HBF_4 (40 wt % in water).

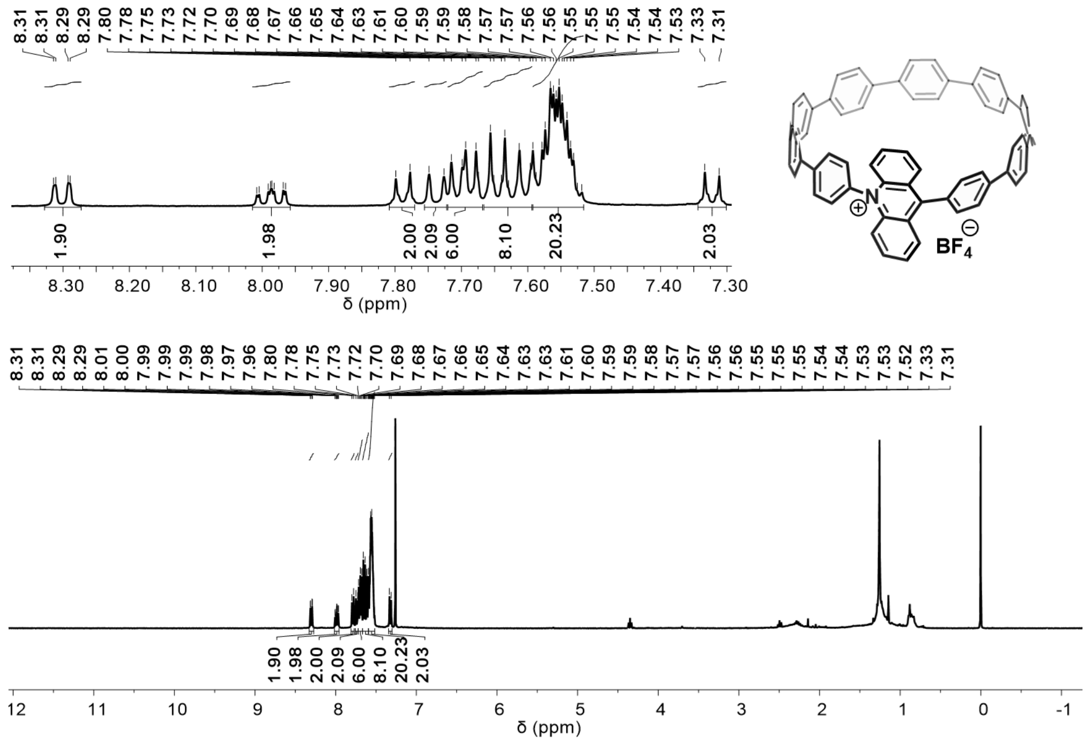

Figure S15. ^{1}H NMR spectra of **Ad[10]CPP·BF$_4$** in $CDCl_3$ (298 K, 400 MHz).

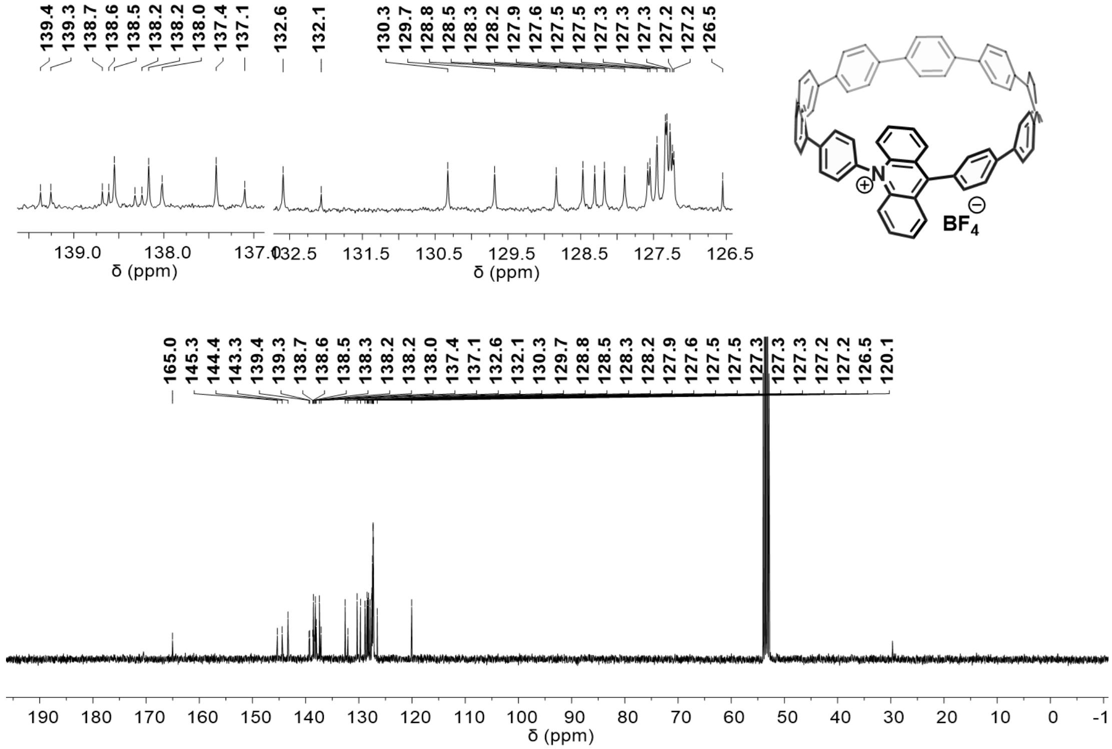

Figure S16. ^{13}C NMR spectra of **Ad[10]CPP·BF$_4$** in $CDCl_3$ (298 K, 101 MHz).

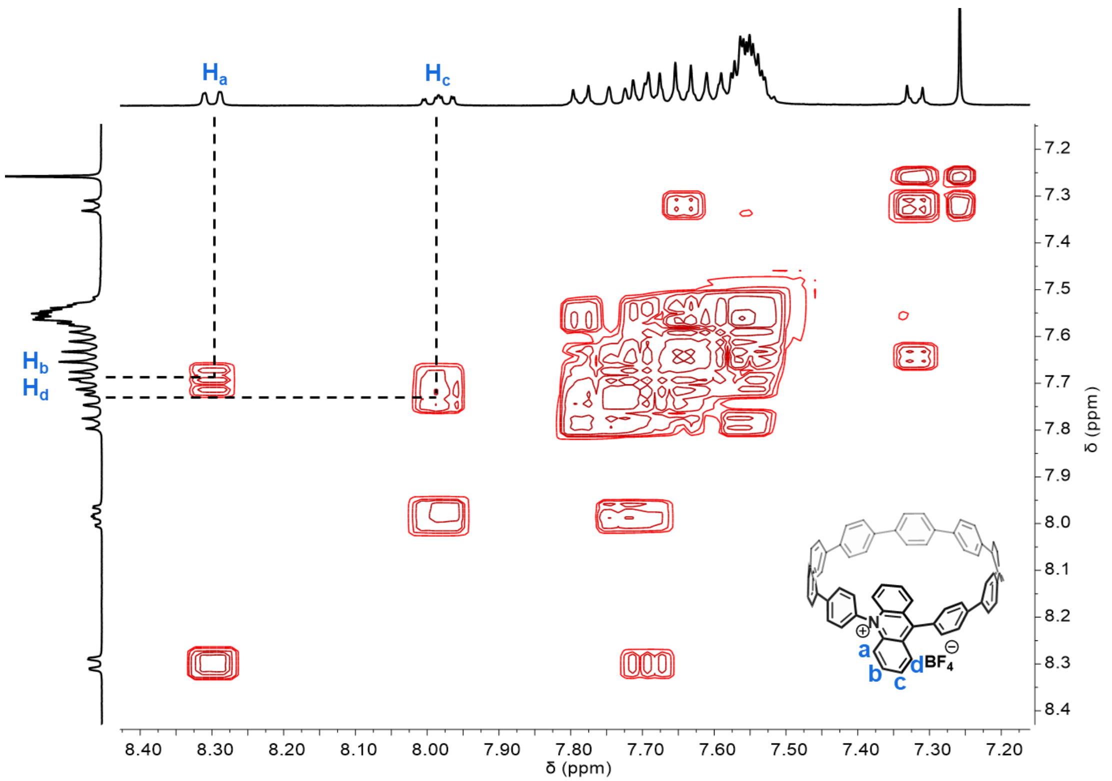

Figure S17. ^{1}H-^{1}H COSY spectra of **Ad[10]CPP·BF$_4$** in $CDCl_3$ (298 K, 400 MHz).

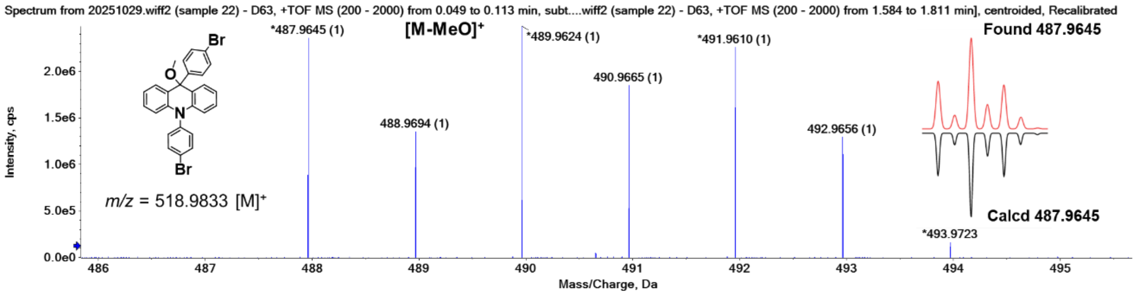

Figure S18. HRMS(ESI) spectrum of **3**.

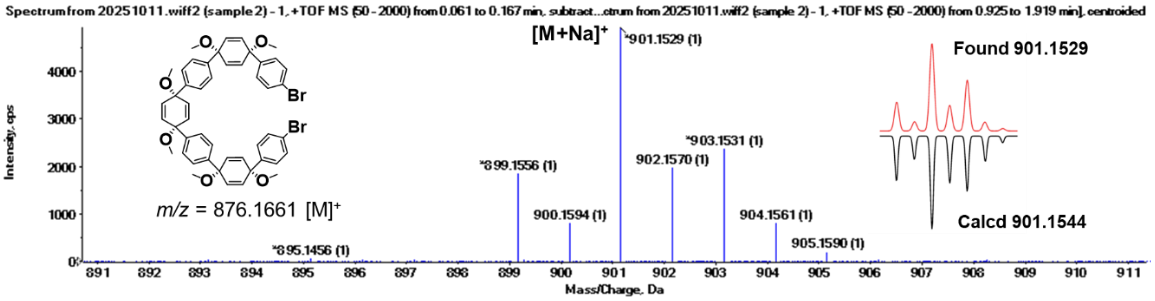

Figure S19. HRMS(ESI) spectrum of **6**(positive ion mode).

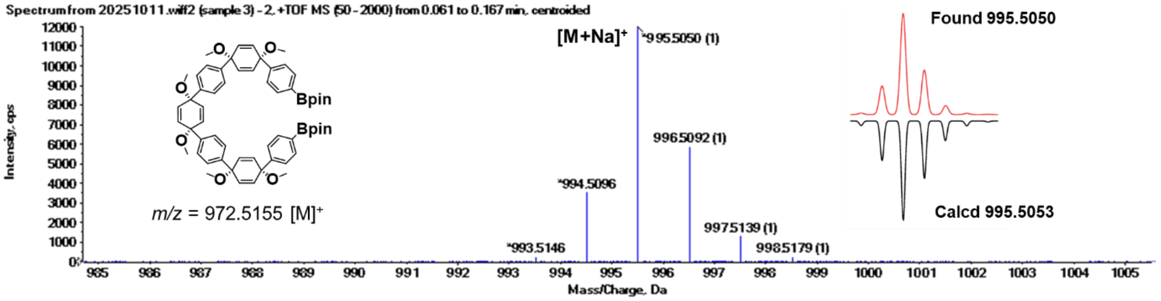

Figure S20. HRMS(ESI) spectrum of **7**(positive ion mode).

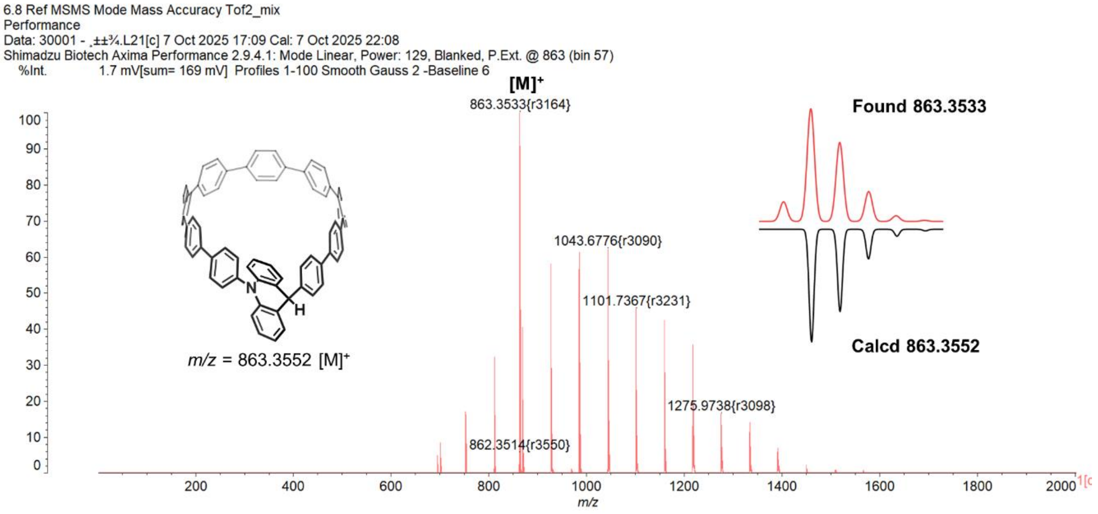

Figure S21. MALDI-TOF-MS spectrum of **Ad[10]CPP-H** (matrix: DCTB, internal standard: PPG, cationization agent: KI, linear mode).

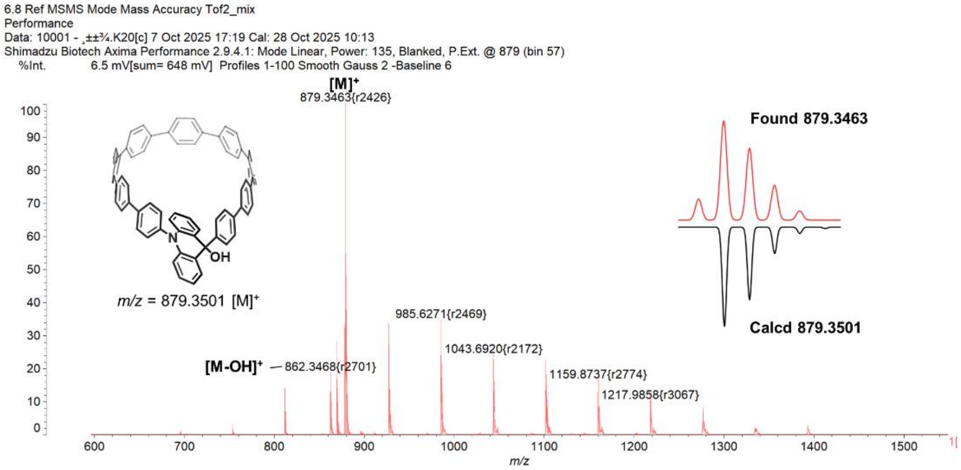

Figure S22. MALDI-TOF-MS spectrum of **Ad[10]CPP-OH** (matrix: DCTB, internal standard: PPG, cationization agent: KI, linear mode).

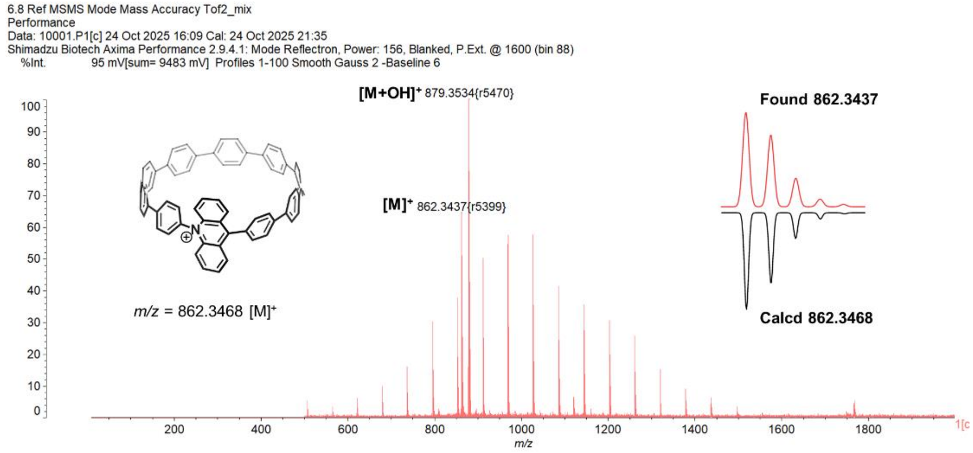

Figure S23. MALDI-TOF-MS spectrum of **Ad[10]CPP·TFA** (matrix: HABA, internal standard: PPG, reflectron mode).

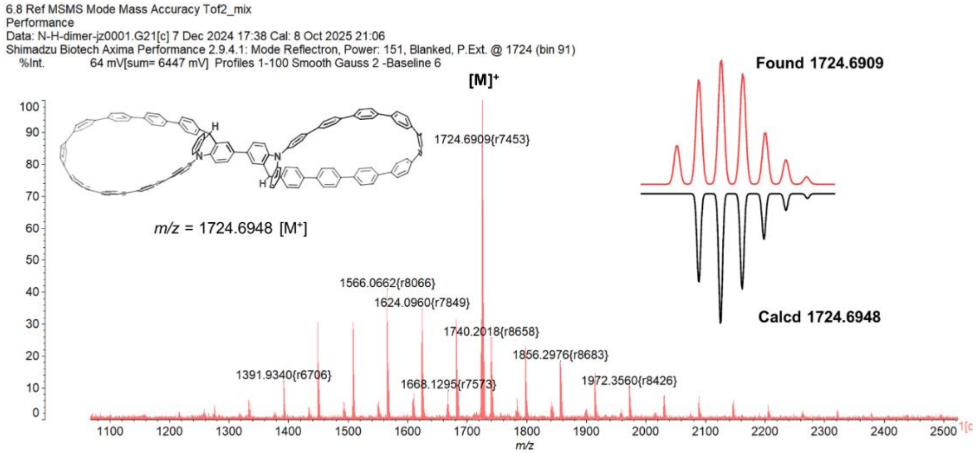

Figure S24. MALDI-TOF-MS spectrum of possible **Ad[10]CPP-dimer** captured (matrix: DCTB, internal standard: PPG, cationization agent: KI, reflectron mode)

4. Photophysical properties for Ad[10]CPP (UV, FL, PLQY and Fluorescence lifetime)

Table S1 Summary of experimental photophysical properties for Ad[10]CPP

Ad[10]CPP	Absorbance (nm)	Extinction coefficient ε (M^{-1} cm^{-1})	Fluorescence (nm)	Fluorescence quantum yield Φ	Singlet lifetime (ns)
**Ad[10]CPP-H**	326, 378	7.3×10^4	467	0.71	2.48
**Ad[10]CPP-OH**	326, 378	7.5×10^4	465	0.67	2.40
**Ad[10]CPP·TFA**	332, 365, 474	6.8×10^4	435	0.01	1.88

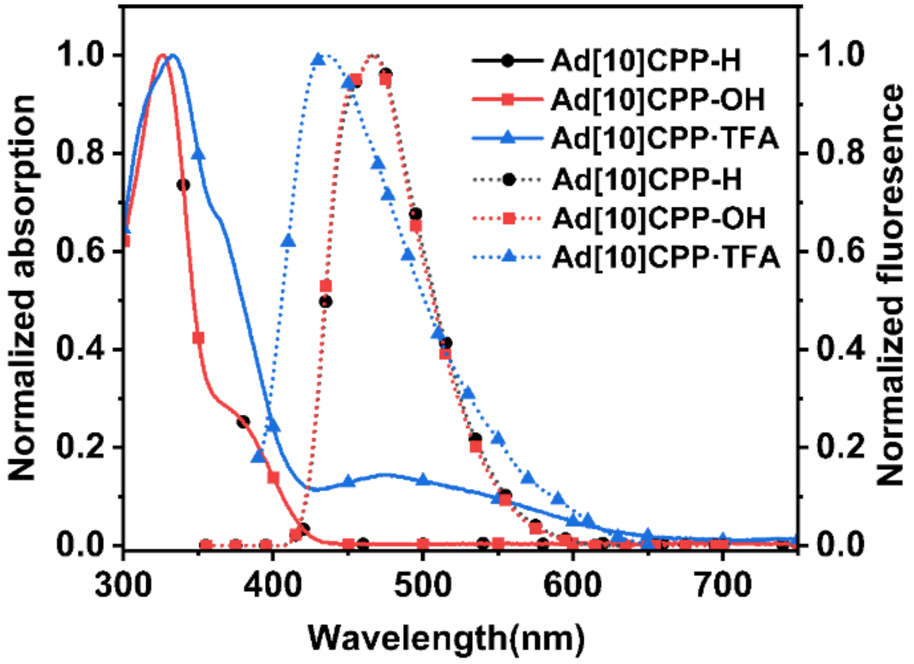

Figure S25. UV-vis absorption (solid line) and fluorescence emission (dotted line) spectra of **Ad[10]CPP-H** (λ_{ex} = 325 nm) (black circle), **Ad[10]CPP-OH** (λ_{ex} = 325 nm) (red square), and **Ad[10]CPP•TFA** (λ_{ex} = 370 nm) (blue triangle) measured in CH_2Cl_2 solutions (1×10^{-5} M) at room temperature. The **Ad[10]CPP•TFA** sample is prepared by adding excess trifluoroacetic acid into **Ad[10]CPP-OH**, with the acid concentration maintained at 1×10^{-3} M.

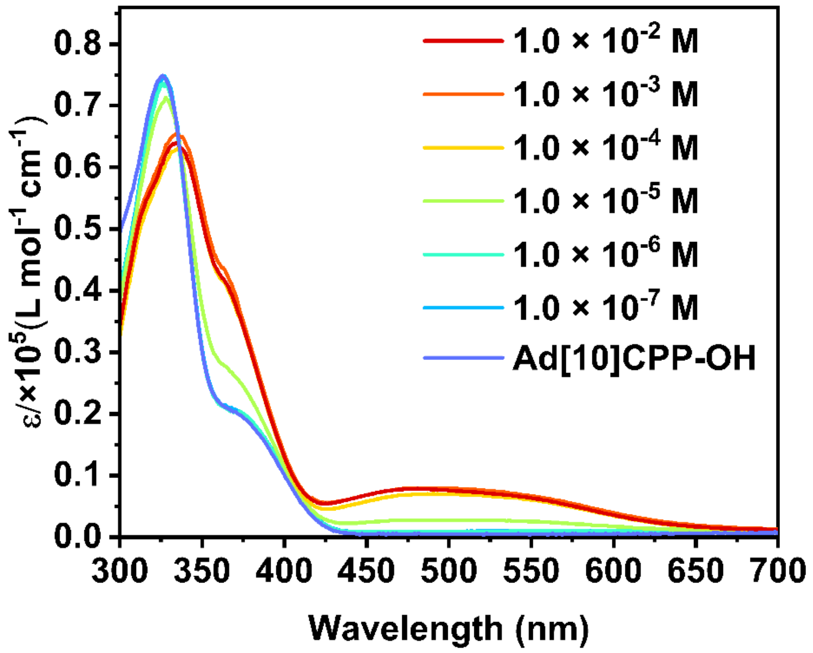

Figure S26. UV-vis spectra of **Ad[10]CPP-OH** in CH_2Cl_2 solutions (1×10^{-5} M) at room temperature with the addition of CF_3COOH at different concentrations.

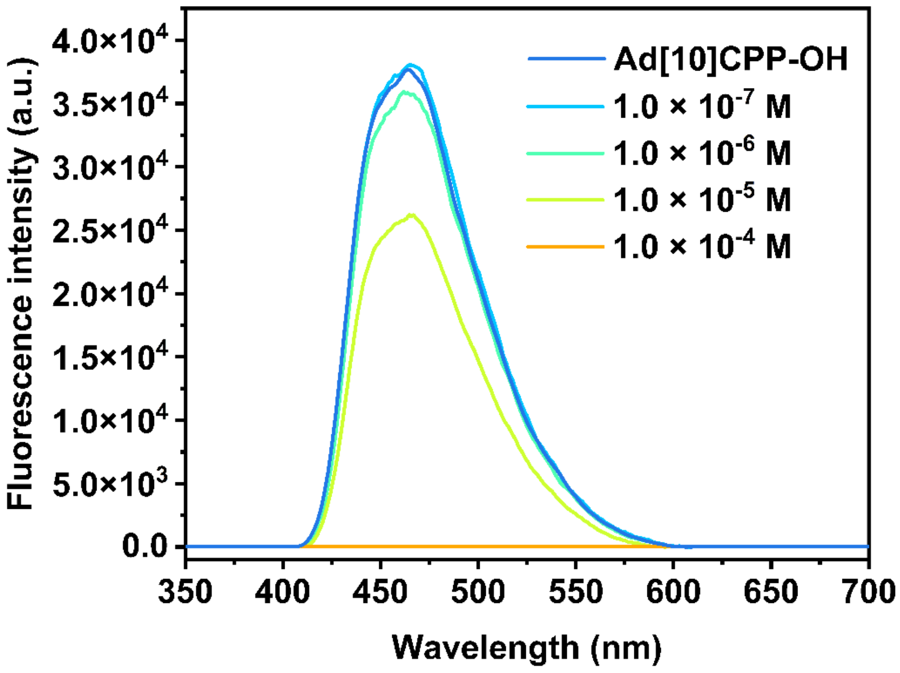

Figure S27. Fluorescence intensity changes of **Ad[10]CPP-OH** in CH_2Cl_2 (1×10^{-5} M) at room temperature (λ_{ex} = 327 nm) with the addition of CF_3COOH at different concentrations.

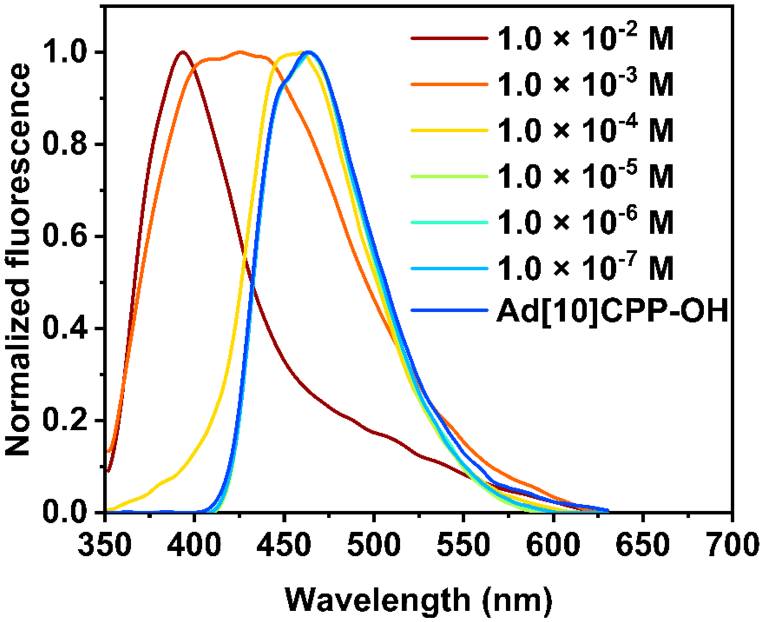

Figure S28. Normalized fluorescence emission spectra of **Ad[10]CPP-OH** (λ_{ex} = 327 nm) in CH_2Cl_2 solutions (1 × 10^{-5} M) at room temperature with the addition of CF_3COOH at different concentrations.

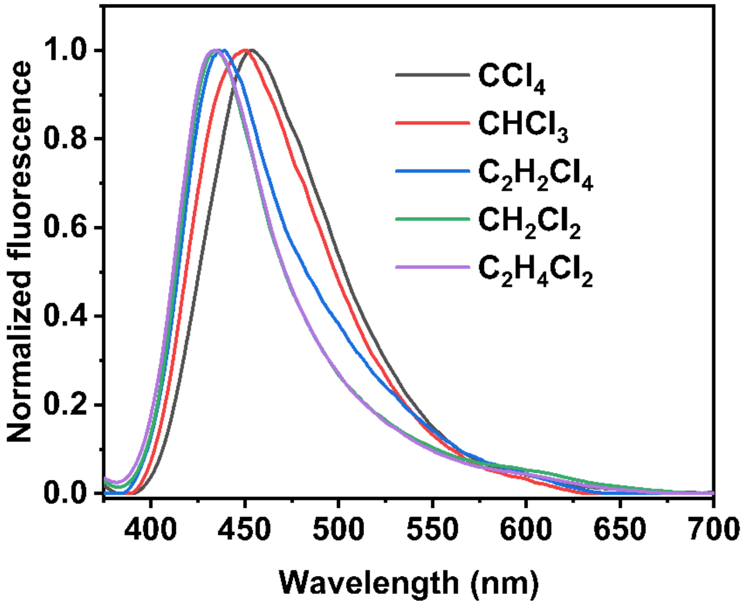

Figure S29. Normalized fluorescence emission spectra of **Ad[10]CPP-OH** (λ_{ex} = 360 nm) in various solvents (1 × 10^{-4} M) at room temperature, with the addition of CF_3COOH (acid concentration: 1 × 10^{-3} M).

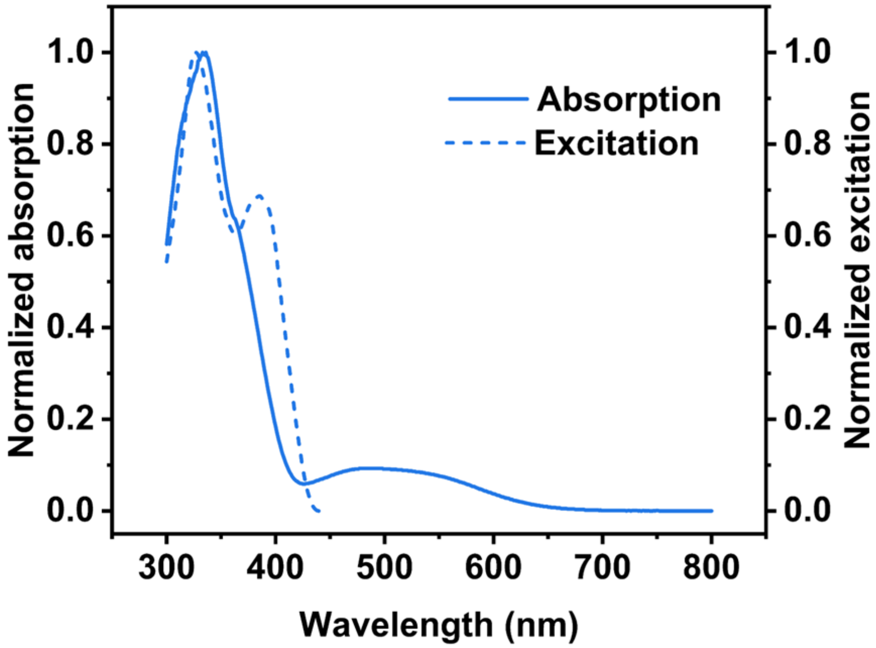

Figure S30. Absorption and excitation spectra (λ_{em}= 459 nm) of **Ad[10]CPP·TFA** in CH_2Cl_2 solutions (1×10^{-5} M) at room temperature (with an acid concentration of 1×10^{-4} M).

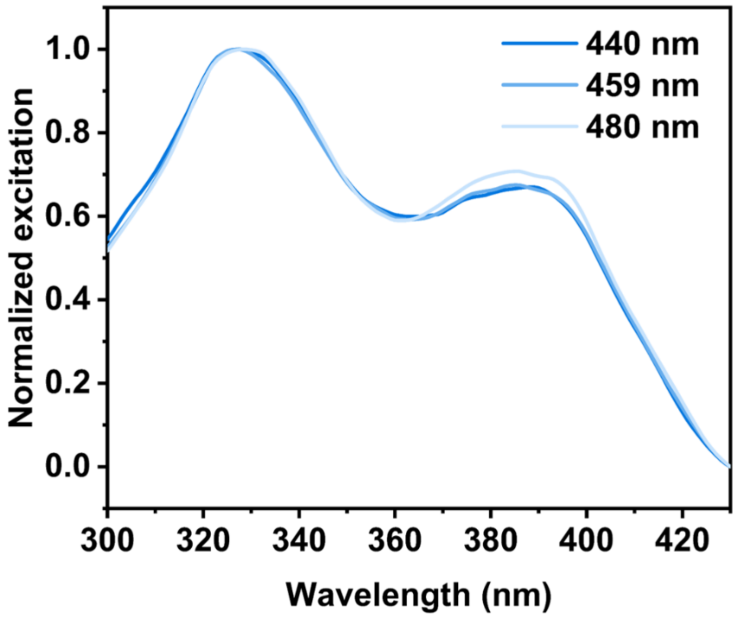

Figure S31. Excitation spectra of **Ad[10]CPP·TFA** measured (λ_{em}= 440 nm, 459 nm, 480 nm) in CH_2Cl_2 solutions (1×10^{-5} M) at room temperature (with an acid concentration of 1×10^{-4} M).

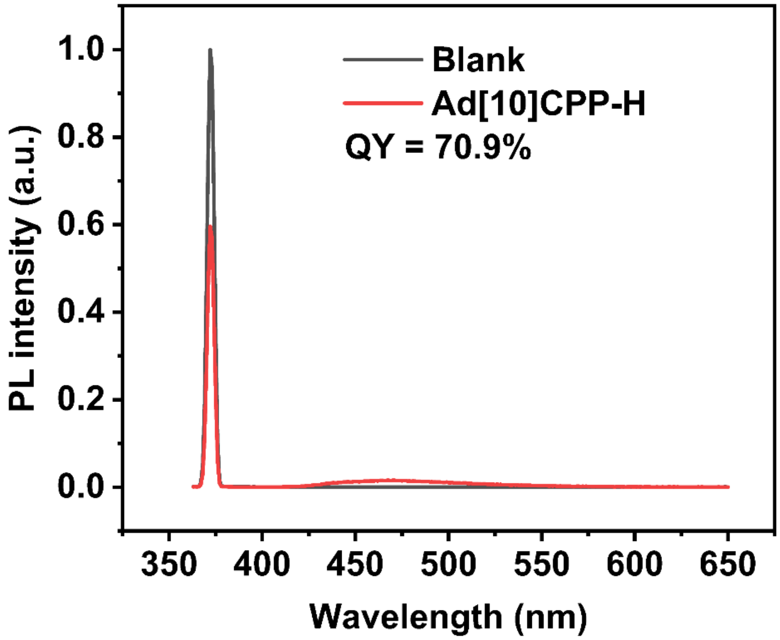

Figure S32. PLQY spectra of **Ad[10]CPP-H** in CH_2Cl_2 solutions (1 × 10^{-5} M) at room temperature.

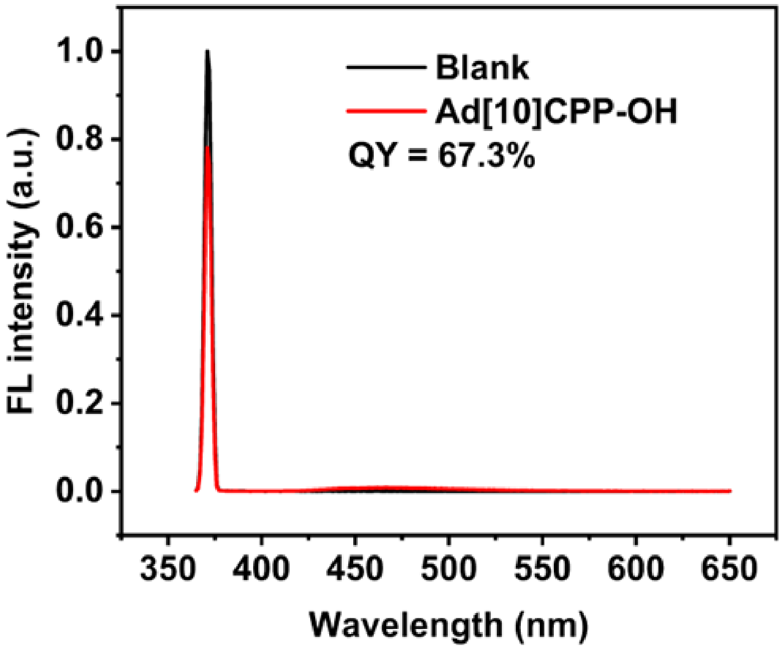

Figure S33. PLQY spectra of **Ad[10]CPP-OH** in CH_2Cl_2 solutions (1 × 10^{-5} M) at room temperature.

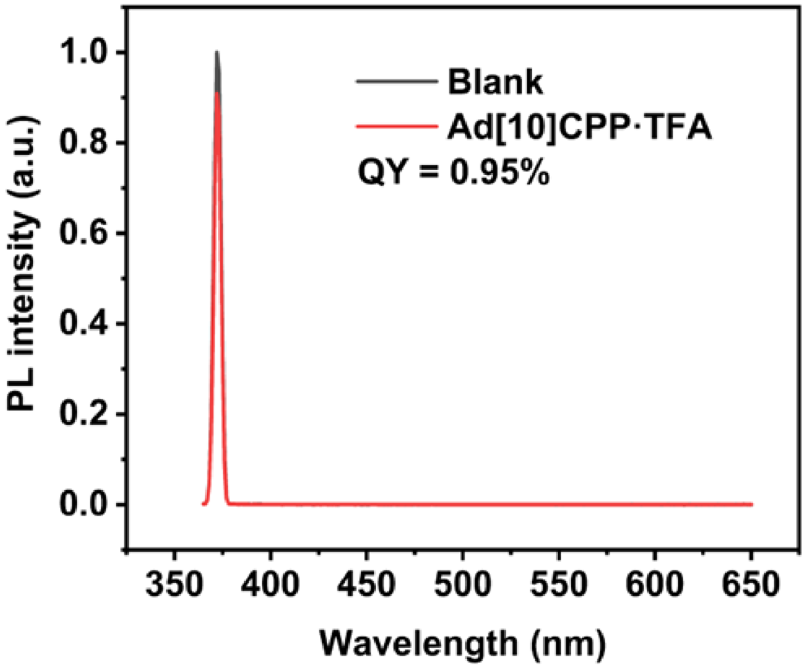

Figure S34. PLQY spectra of **Ad[10]CPP·TFA** in CH_2Cl_2 solutions (1×10^{-5} M) at room temperature.

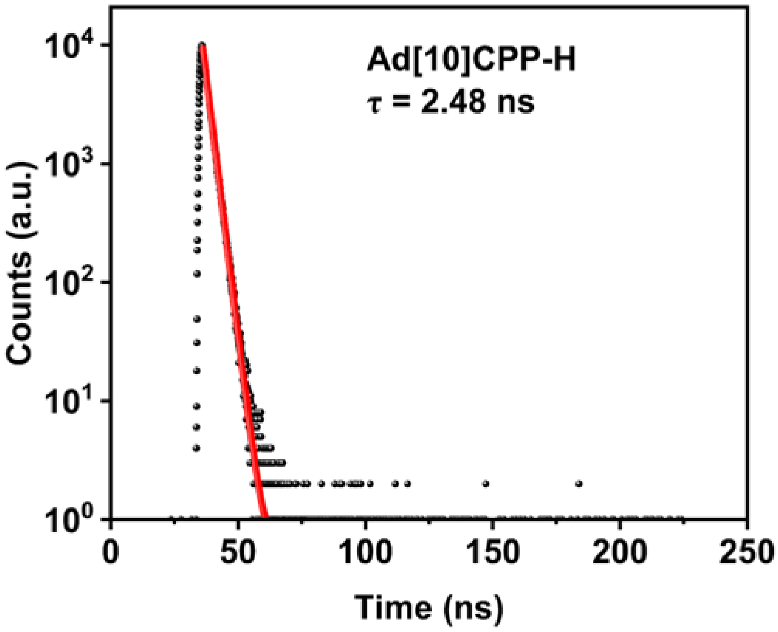

Figure S35. Fluorescence decay profiles of **Ad[10]CPP-H** measured at 458 nm in CH_2Cl_2 solution (1×10^{-5} M, room temperature). Data was acquired by time-resolved fluorescence spectroscopy.

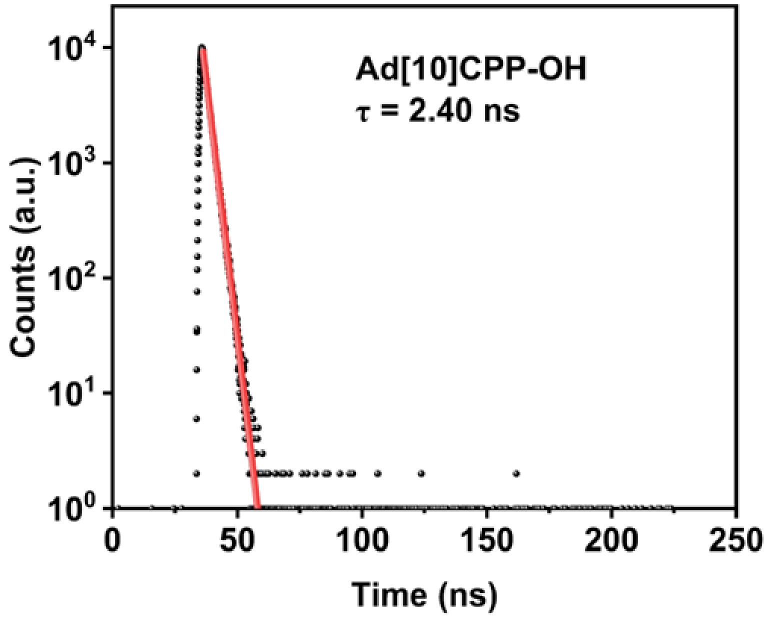

Figure S36. Fluorescence decay profiles of **Ad[10]CPP-OH** measured at 458 nm in CH_2Cl_2 solution (1 × 10^{-5} M, room temperature). Data was acquired by time-resolved fluorescence spectroscopy.

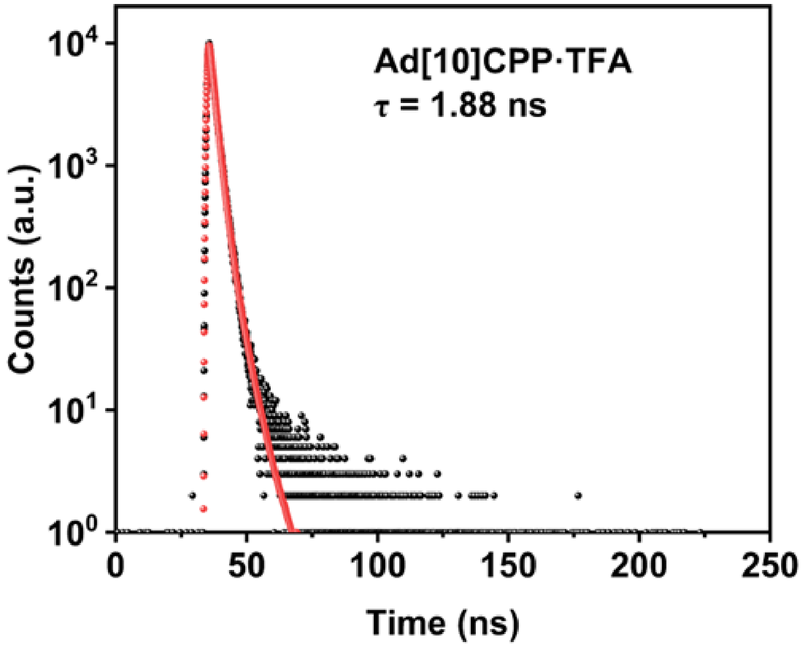

Figure S37. Fluorescence decay profiles of **Ad[10]CPP·TFA** measured at 435 nm in CH_2Cl_2 solution (1 × 10^{-5} M, room temperature). Data was acquired by time-resolved fluorescence spectroscopy.

The radiative decay constants (k_r) and non-radiative decay constants (k_{nr}) are calculated by means of the following relations:

$$k_{\mathrm{r}} = \Phi_{\mathrm{F}}/\tau \text{ and } k_{\mathrm{nr}} = (1 - \Phi_{\mathrm{F}})/\tau.$$

Φ_{F} is the fluorescence quantum yield, τ is the lifetime of the excited state.

From the estimated fluorescence quantum yield (Φ_{F}) and τ values, the radiative decay constants (k_r) are calculated to be 2.86×10^8 s^{-1} for **Ad[10]CPP-H**, 2.80×10^8 s^{-1} for **Ad[10]CPP-OH**, and 5.1×10^6 s^{-1} for **Ad[10]CPP$^+$**. The non-radiative decay constants (k_{nr}) are calculated to be 1.17×10^8 s^{-1} for **Ad[10]CPP-H**, 1.36×10^8 s^{-1} for **Ad[10]CPP-OH**, and 5.3×10^8 s^{-1} for **Ad[10]CPP$^+$**,

5. Electrochemical Characterization (CV and DPV)

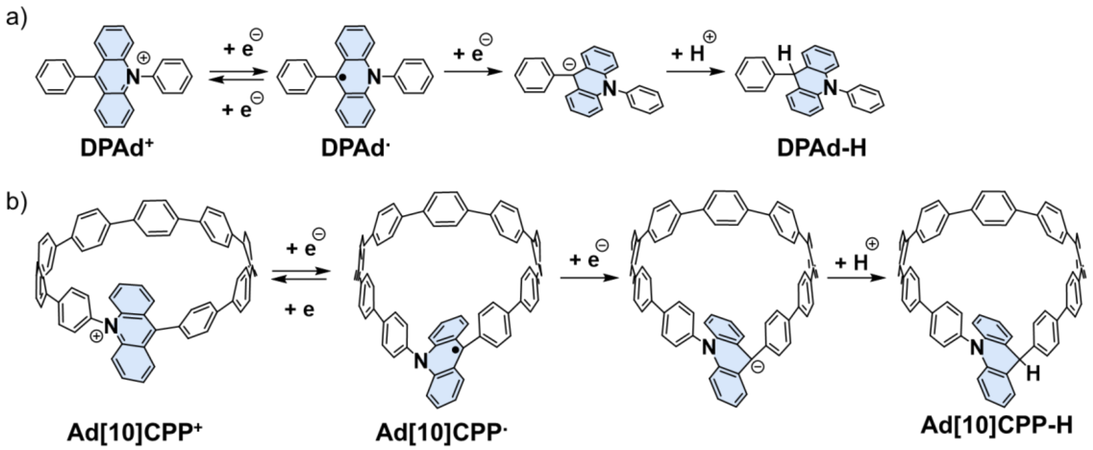

Figure S38. The stepwise reduction mechanism diagram of the a) **DPAd**$^{+}$ and b) **Ad[10]CPP**$^{+}$.

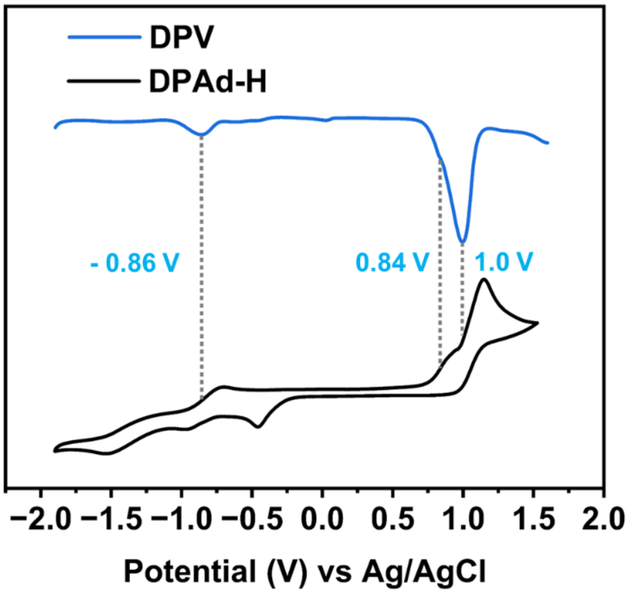

Figure S39. Cyclic voltammogram and differential pulse voltammogram of 2.0 mM **DPAd-H** in CH_2Cl_2 solution (V vs Ag/AgCl, in 0.1 M n-Bu_4NPF_6 / CH_2Cl_2, scan rate: 100 mV/s for CV, room temperature).

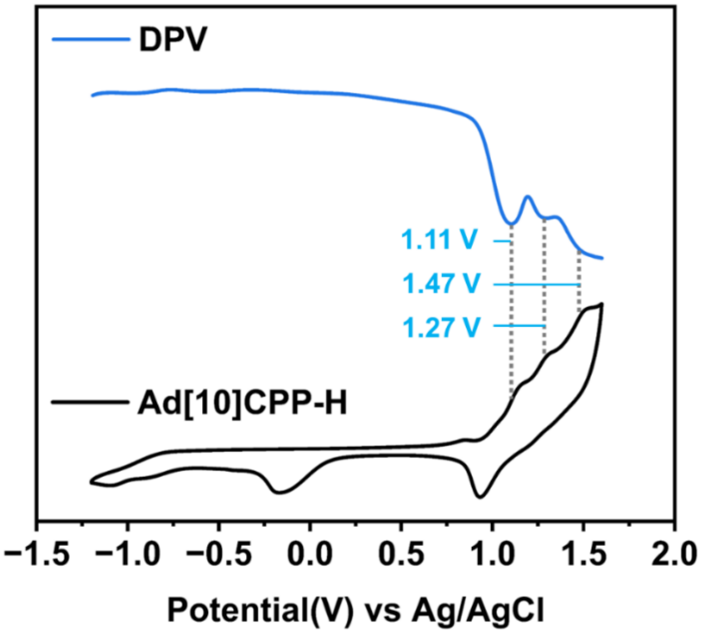

Figure S40. Cyclic voltammogram and differential pulse voltammogram of 1.0 mM **Ad[10]CPP-H** in CH_2Cl_2 (V vs Ag/AgCl, in 0.1 M n-Bu_4NPF_6 / CH_2Cl_2, scan rate: 100 mV/s for CV, room temperature)

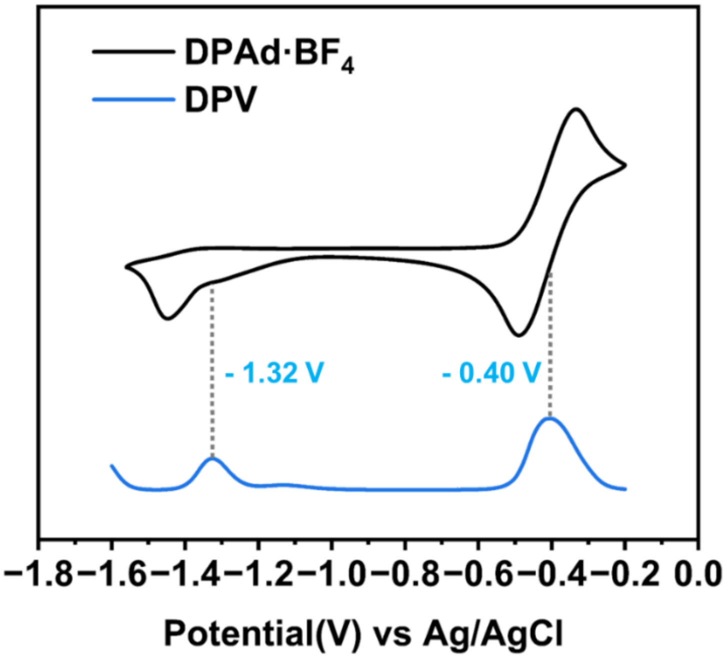

Figure S41. Cyclic voltammogram and differential pulse voltammogram of 1.0 mM **DPAd·BF_4** (V vs Ag/AgCl, in 0.1 M n-Bu_4NPF_6 / CH_2Cl_2, scan rate: 100 mV/s for CV, room temperature)

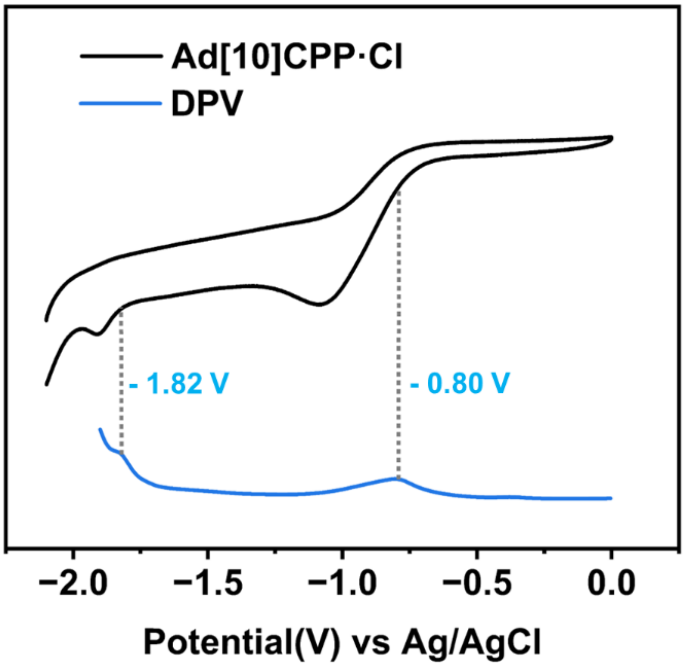

Figure S42. Cyclic voltammogram and differential pulse voltammogram of 1.0 mM **Ad[10]CPP·Cl** in CH_3CN solution (V vs Ag/AgCl, in 0.1 M n-Bu_4NBF_4 / CH_3CN, scan rate: 100 mV/s for CV, room temperature).

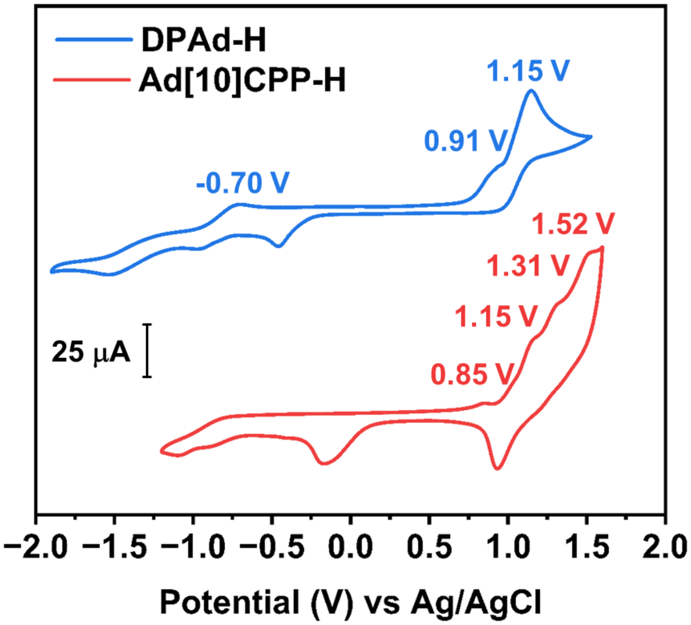

Figure S43. Comparison diagram of Cyclic voltammograms of 2.0 mM **DPAd-H** and 1.0 mM **Ad[10]CPP-H** in CH_2Cl_2 solution (V vs Ag/AgCl, in 0.1 M n-Bu_4NPF_6 / CH_2Cl_2, scan rate: 100 mV/s for CV, room temperature).

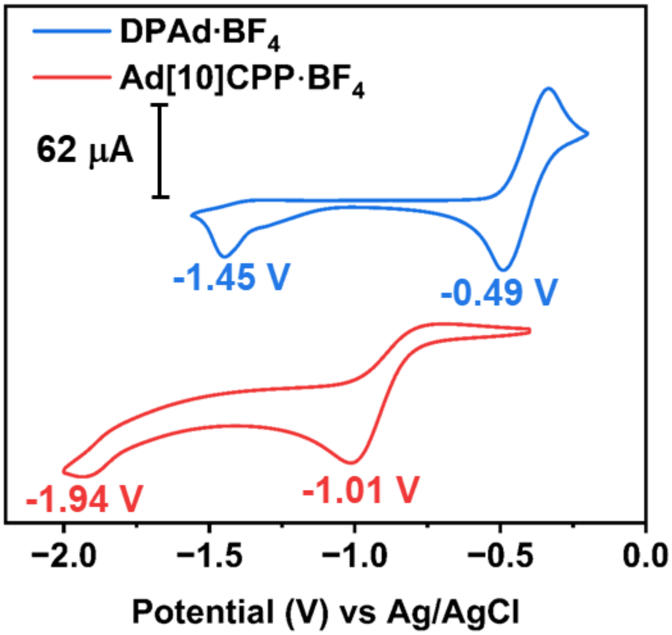

Figure S44. Comparison diagram of Cyclic voltammograms of 1.0 mM **DPAd·BF$_4$** in CH_2Cl_2 solution (V vs Ag/AgCl, in 0.1 M n-Bu_4NPF_6 / CH_2Cl_2 scan rate: 100 mV/s for CV, room temperature) and **Ad[10]CPP·BF$_4$** in CH_3CN solution (V vs Ag/AgCl, in 0.1 M n-Bu_4NBF_4 / CH_3CN, scan rate: 100 mV/s for CV, room temperature).

6. Chemical Oxidation Experiments (UV-vis-IR, EPR)

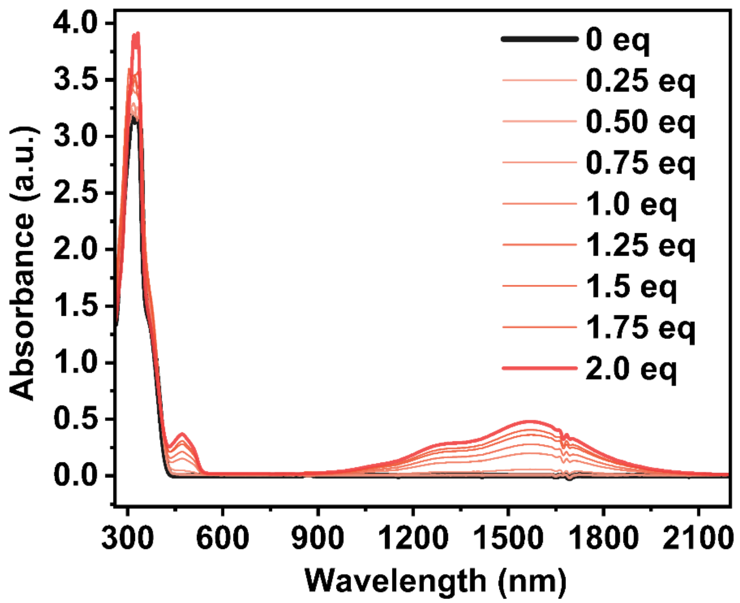

Figure S45. UV-vis-NIR absorption spectra of **Ad[10]CPP-H** and **Ad[10]CPP-H** with 0 to 2 equivalents of $NOBF_4$ in CH_2Cl_2 solutions (5×10^{-4} M).

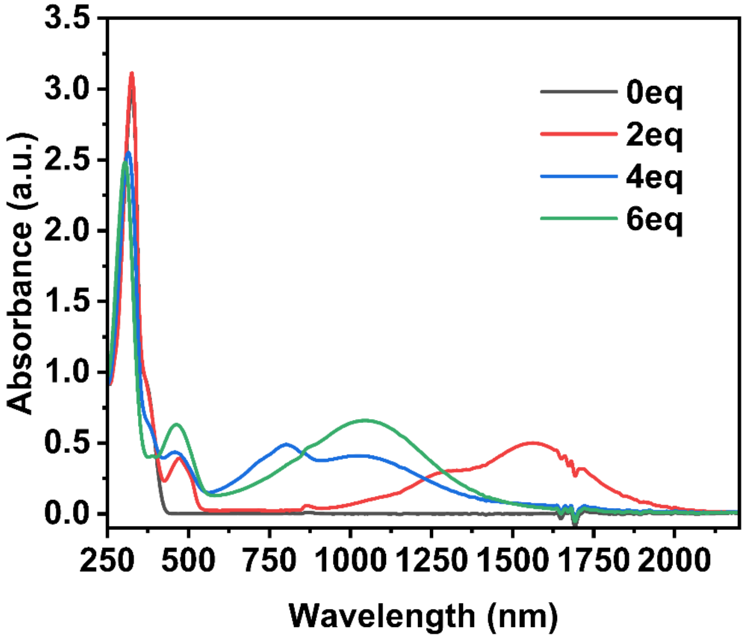

Figure S46. UV-vis-NIR absorption spectra of **Ad[10]CPP-H** and **Ad[10]CPP-H** with 0 to 6 equivalents of $NOBF_4$ in CH_2Cl_2 solutions (5×10^{-4} M).

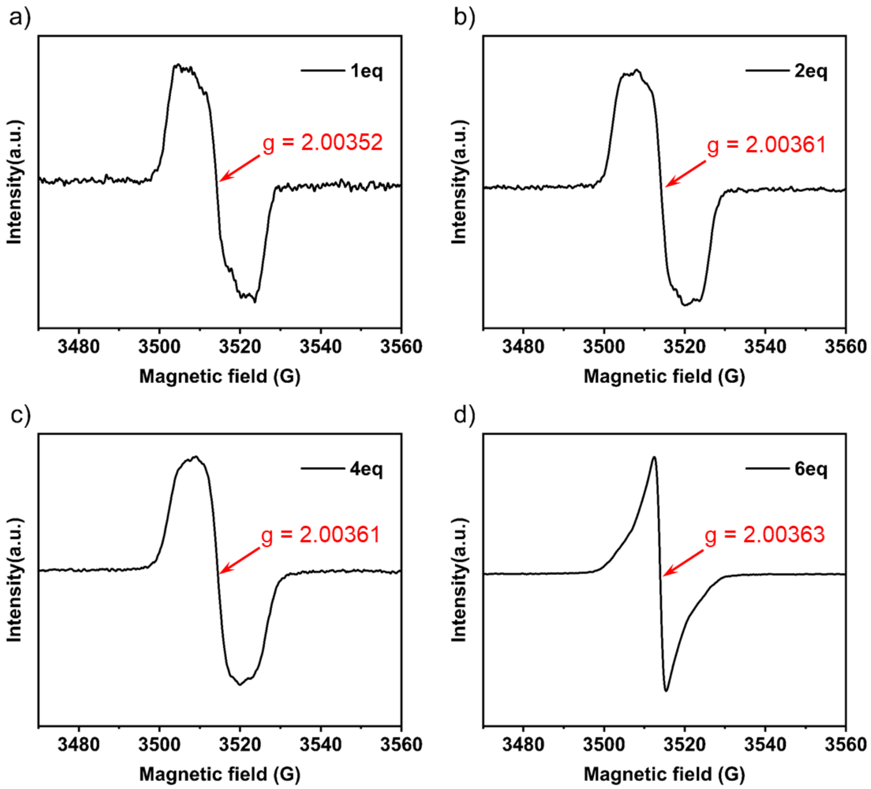

Figure S47. EPR spectra of the oxidation of **Ad[10]CPP-H** by 1 to 6 equivalents of $NOBF_4$ in CH_2Cl_2 solutions. (2×10^{-3} M).

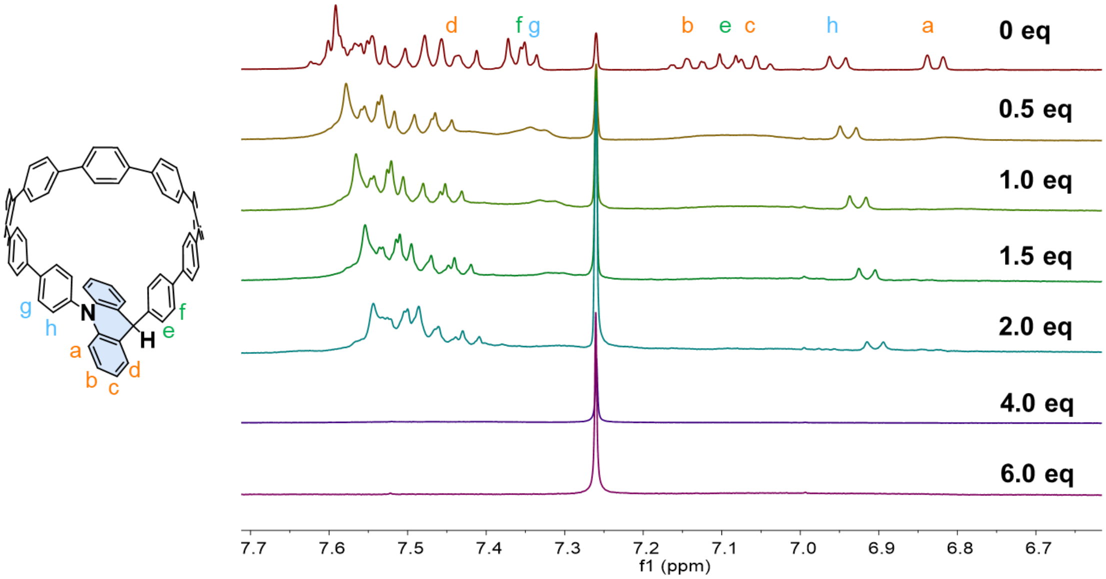

Figure S48. Partial ^{1}H NMR spectra of the oxidation of **Ad[10]CPP-H** by 0 to 6 equivalents of $NOBF_4$ in $CDCl_3$ solutions (2×10^{-3} M).

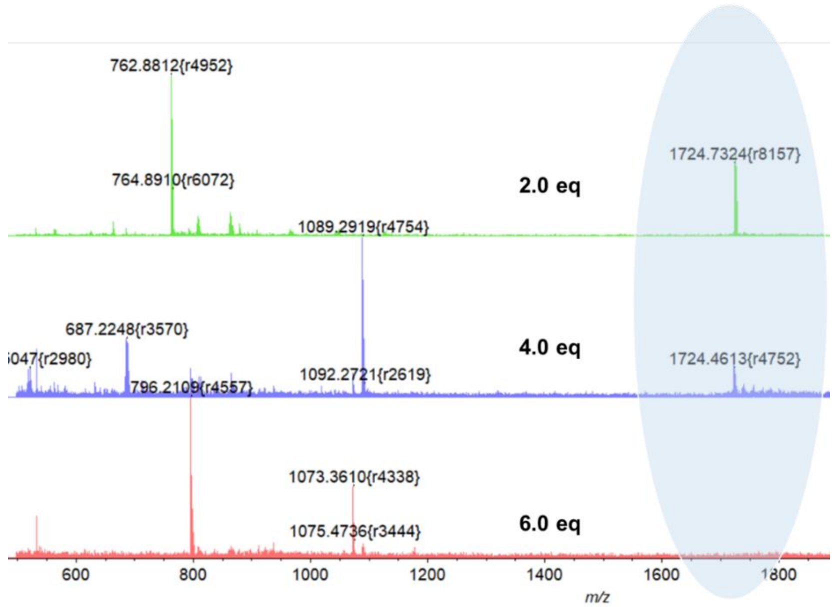

Figure S49. MALDI-TOF-MS spectrum of the oxidation of **Ad[10]CPP-H** by 2 to 6 equivalents of $NOBF_4$ in CH_2Cl_2 solution.

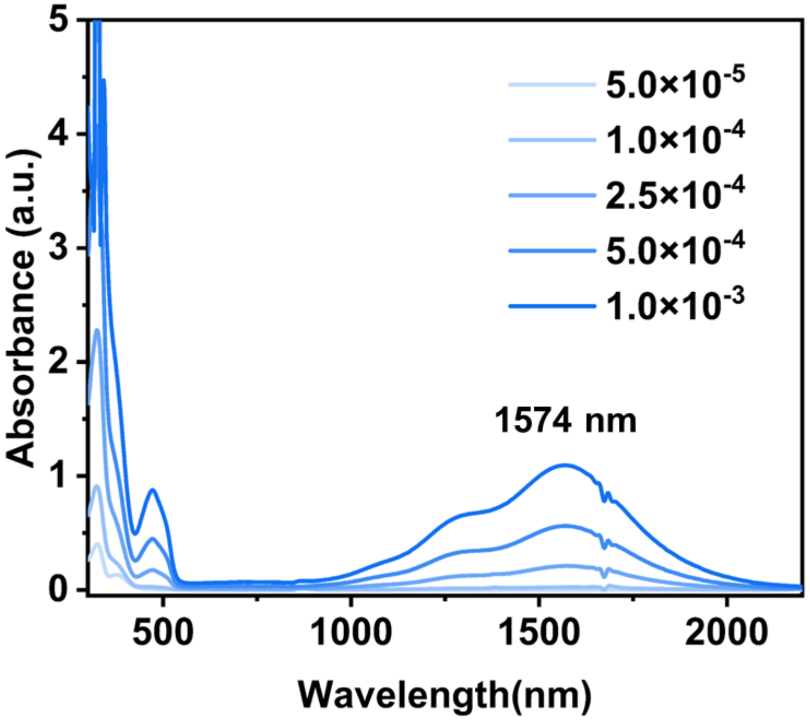

Figure S50. UV-vis-NIR absorption spectra of **Ad[10]CPP-H** with two equivalents of $NOBF_4$ recorded over a range of different concentrations CH_2Cl_2 solutions.

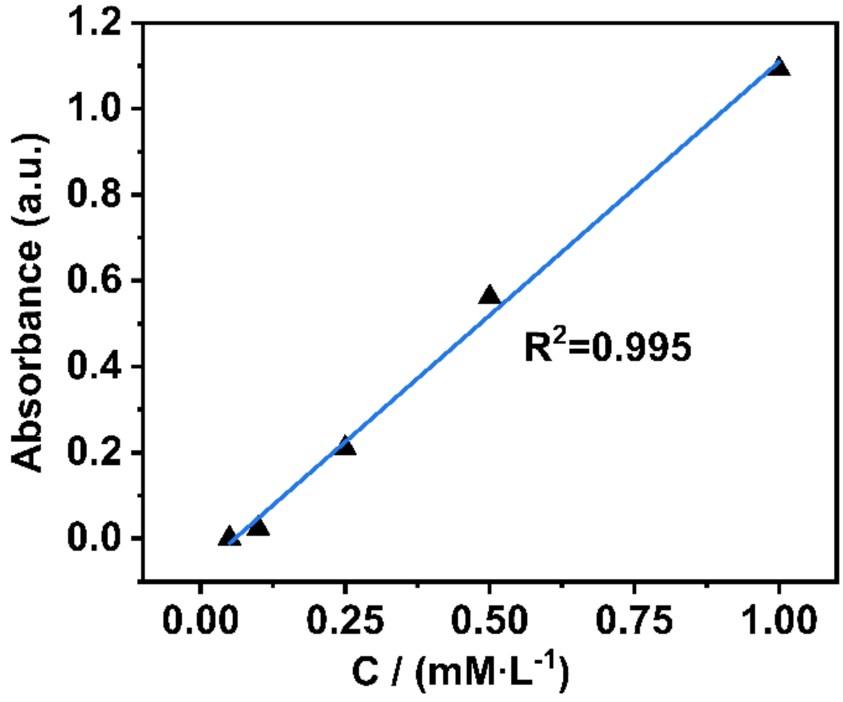

Figure S51. Dependence of the intensity of the band at 1574 nm on concentration.

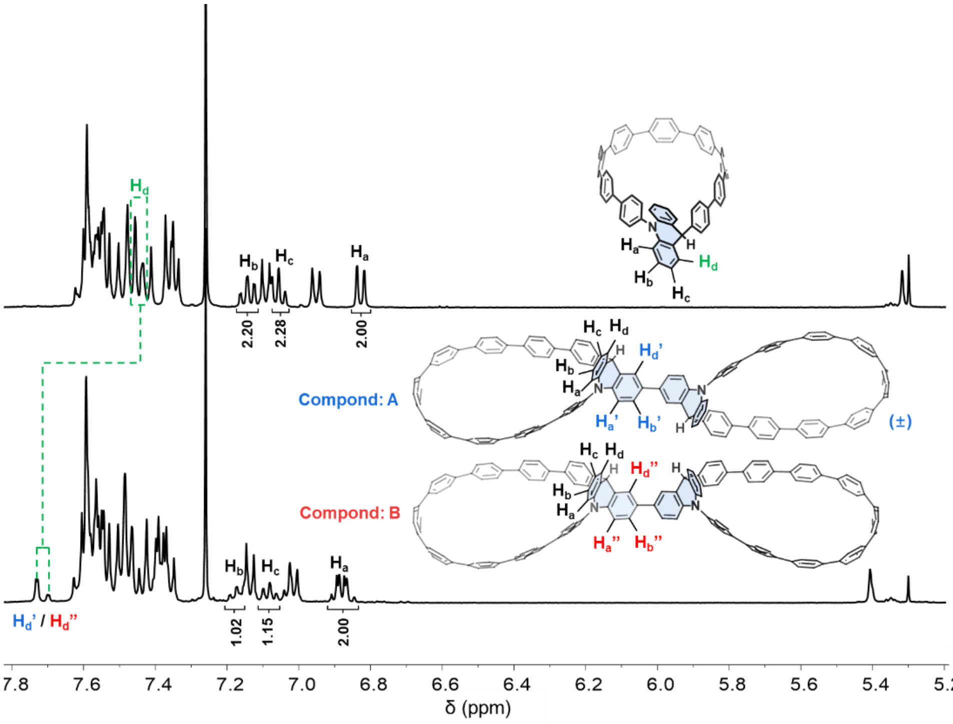

Figure S52. ^{1}H NMR spectra of **Ad[10]CPP-dimer** in $CDCl_3$ (298 K, 400 MHz).

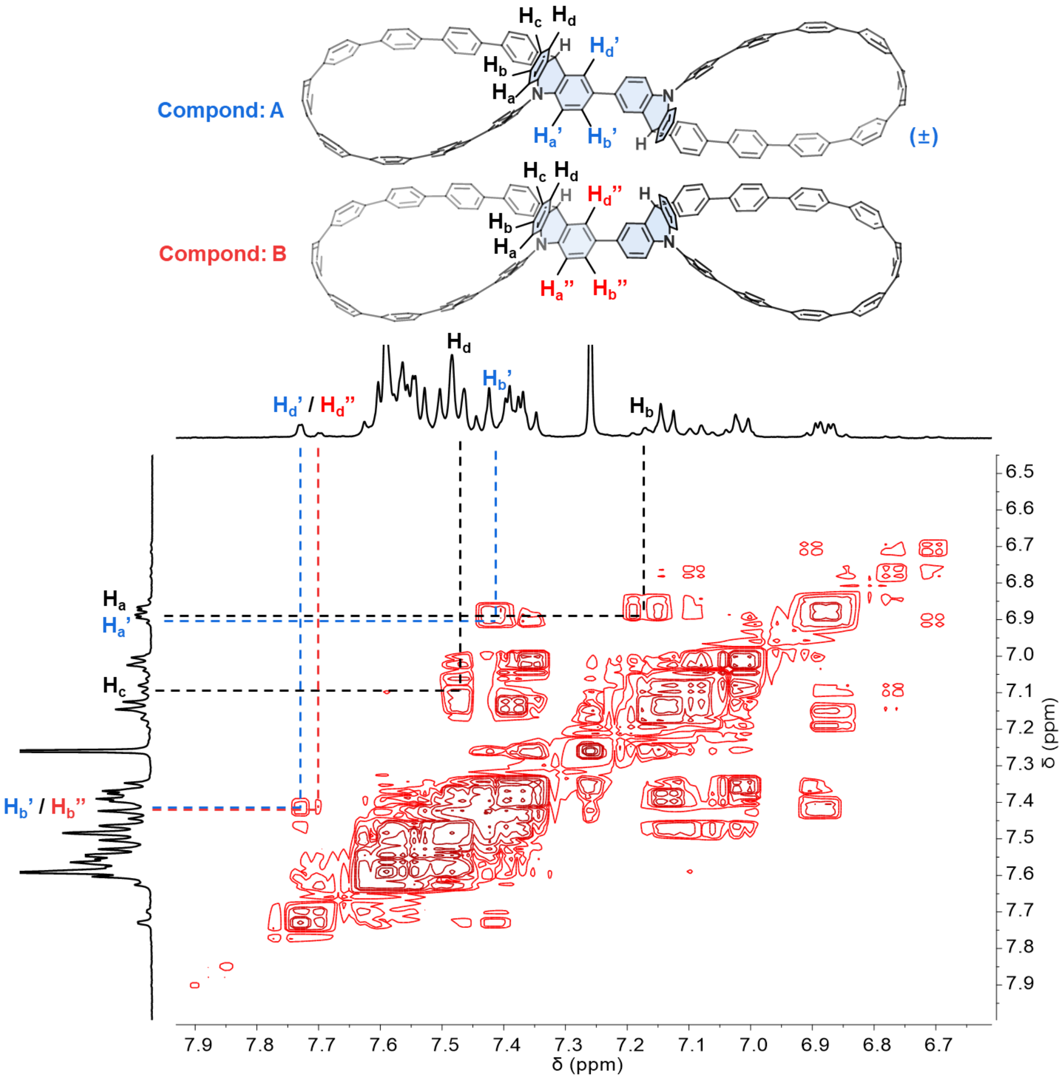

Figure S53. ${}^{1}H$-${}^{1}H$ COSY spectra of **Ad[10]CPP-dimer** in $CDCl_3$ (298 K, 400 MHz).

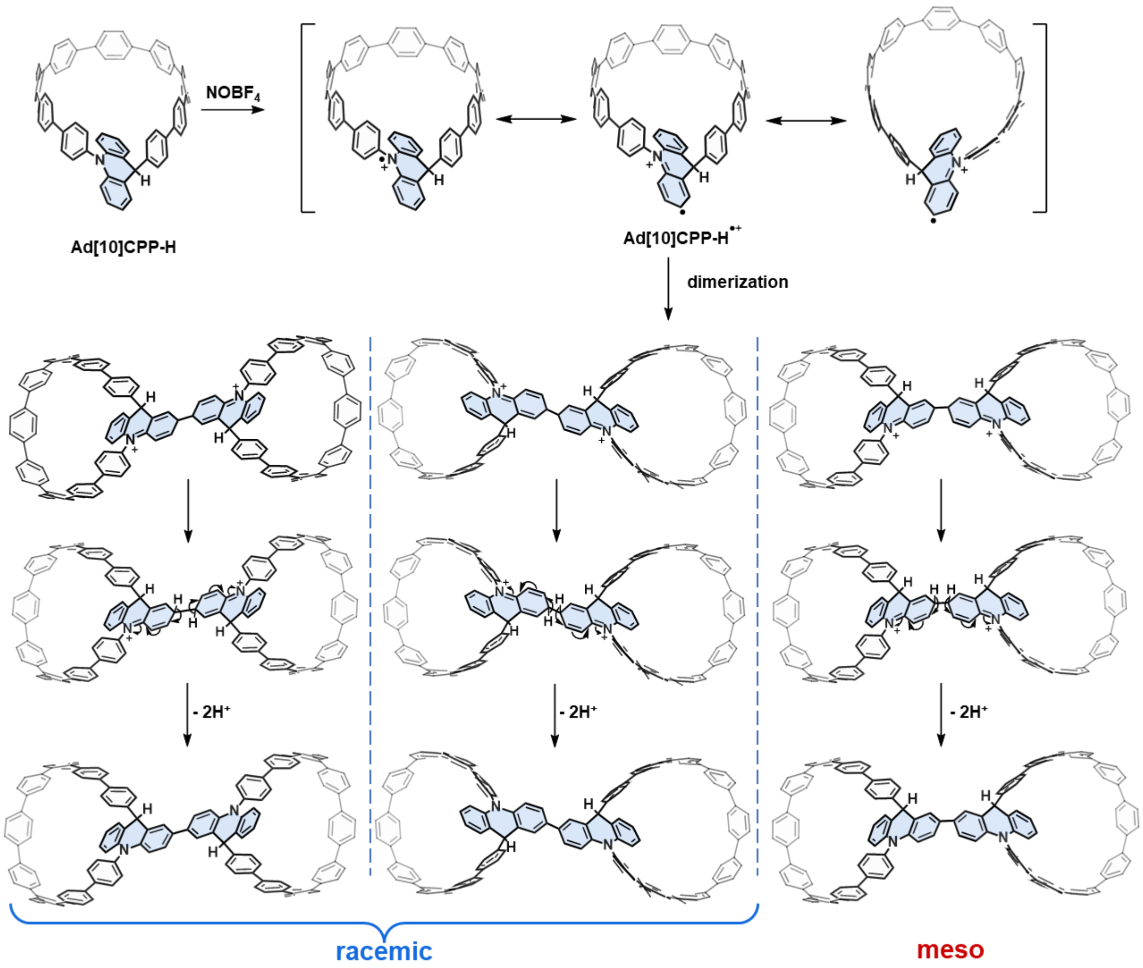

Figure S54. A plausible mechanism for the formation of **Ad[10]CPP-dimer**.

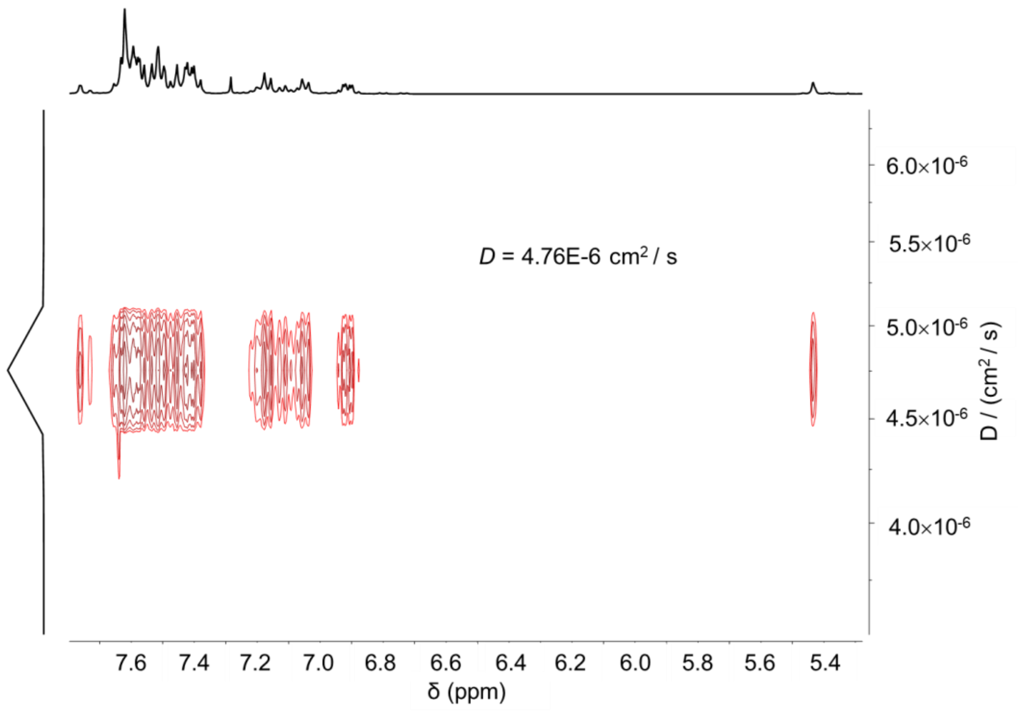

Figure S55. Partial DOSY spectrum of **Ad[10]CPP-dimer** in $CDCl_3$ (298 K, 400 MHz).

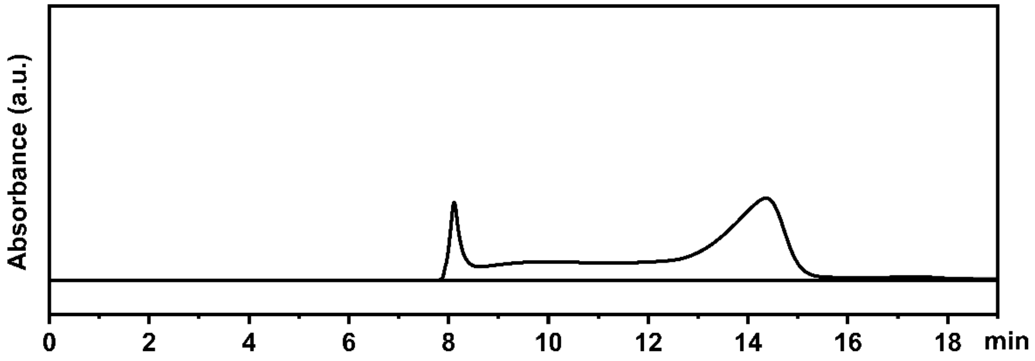

Figure S56. HPLC chromatogram of **Ad[10]CPP-dimer**, eluent: 1:1 CH_2Cl_2/n-hexane, flow rate: 2 mL/min, detection wavelength: 326 nm.

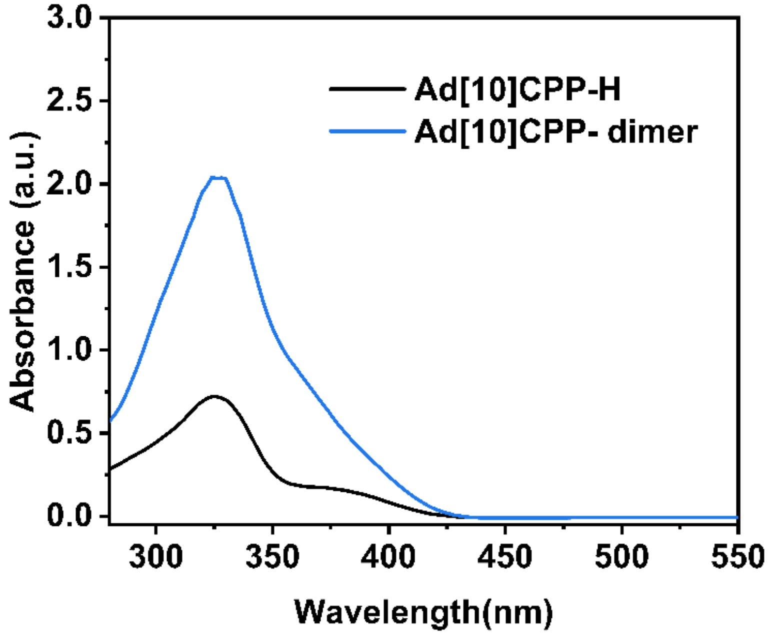

Figure S57. UV-vis absorption spectra of **Ad[10]CPP-H** in CH_2Cl_2 solution (1×10^{-4} M) (blank line) and **Ad[10]CPP-dimer** in CH_2Cl_2 solution (1×10^{-4} M) (blue line).

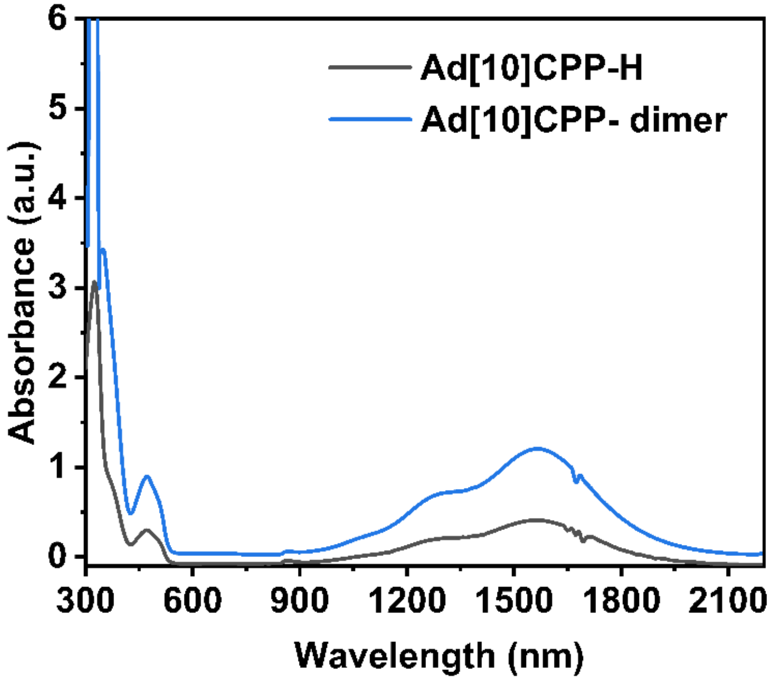

Figure S58. UV-vis-NIR absorption spectra of **Ad[10]CPP-H** with one equivalent of $NOBF_4$ in CH_2Cl_2 solution (5×10^{-4} M) (blank line), and **Ad[10]CPP-dimer** with one equivalent of $NOBF_4$ in CH_2Cl_2 solutions (5×10^{-4} M) (blue line).

7. Complexation of Ad[10]CPP·TFA with C_{60}

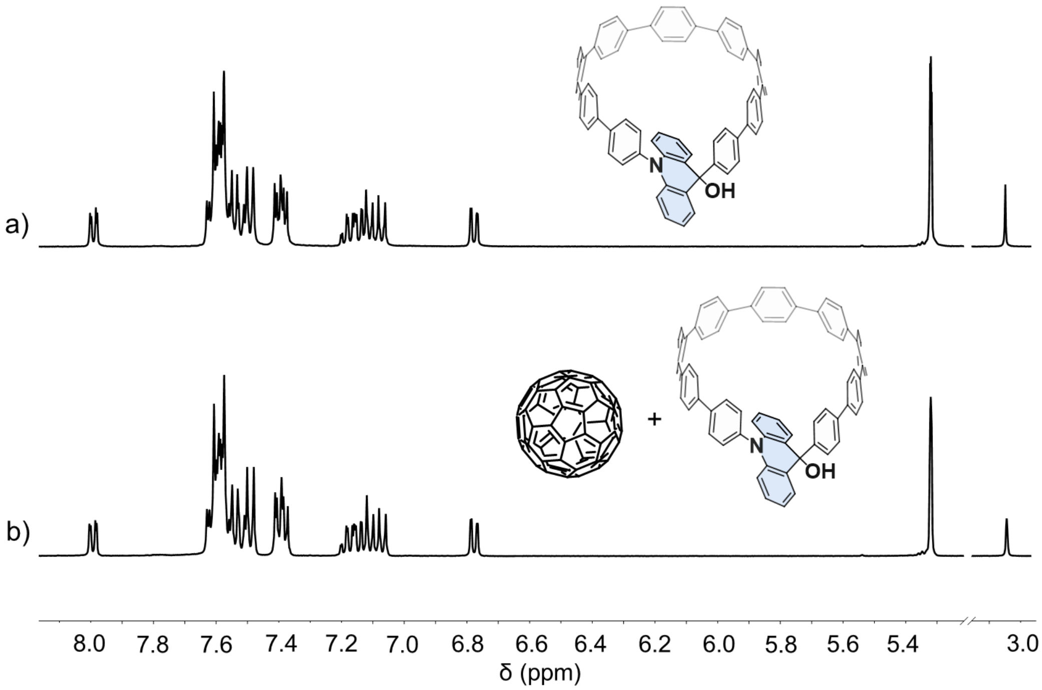

Figure S59. ^{1}H NMR spectra of **Ad[10]CPP-OH** in CD_2Cl_2 a) before and b) after addition to C_{60} (298 K, 400 MHz).

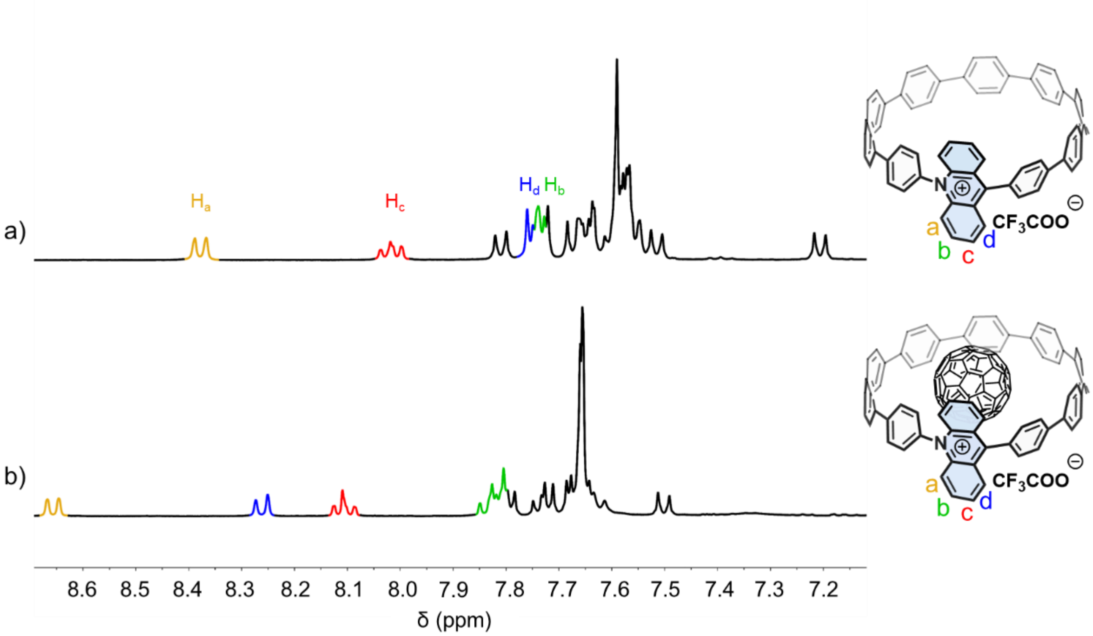

Figure S60. ^{1}H NMR spectra of **Ad[10]CPP·TFA** in CD_2Cl_2 a) before and b) after addition to C_{60} (298 K, 400 MHz).

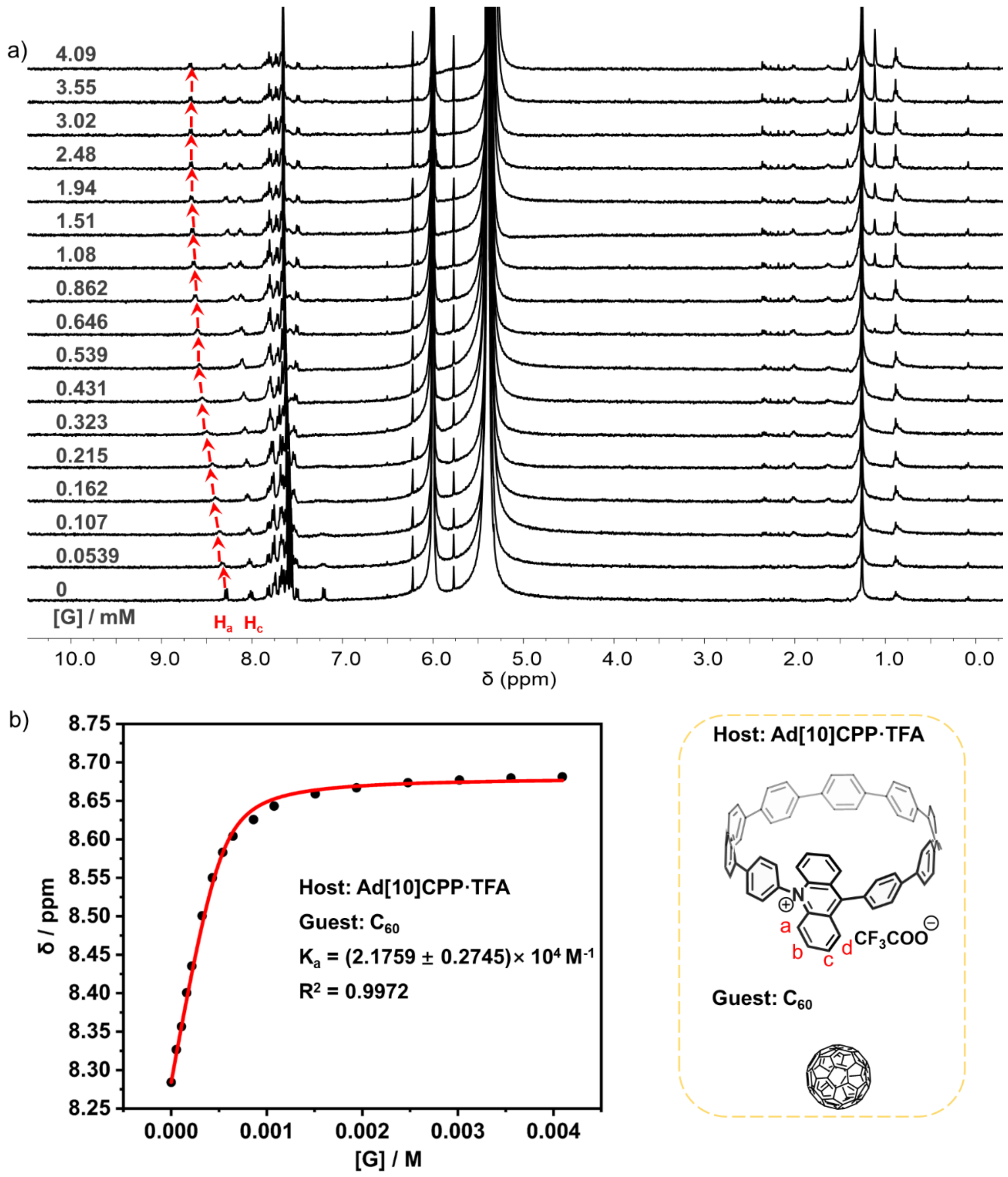

Figure S61. ^{1}H NMR titration experiments. a) ^{1}H NMR spectra (400 MHz, $C_2D_2Cl_4$-d_2, 298 K) of **Ad[10]CPP·TFA** (0.53 mM) titrated by $\mathbf{C_{60}}$, the concentration range of $\mathbf{C_{60}}$ is 0 ~ 4.09 mM, the changes of signals of **proton a** with the addition of $\mathbf{C_{60}}$ are marked with red arrow; b) Nonlinear fitting for the complexation between **Ad[10]CPP·TFA** and $\mathbf{C_{60}}$ based **proton a**.

Speciation Analysis for the Binding Experiment

The binding between **Ad[10]CPP**$^{+}$ (host) and $\mathbf{C_{60}}$ (guest) follows a 1:1 stoichiometry:

$$\text{Host} + \text{Guest} \rightleftharpoons \text{Complex}$$

The binding constant K_a is defined as:

$$K_a = \frac{[\text{Complex}]}{[\text{Host}]_{\text{free}}[\text{Guest}]_{\text{free}}}$$

Given the total concentrations of host and guest:

$$[\text{Host}]_{\text{total}} = [\text{Host}]_{\text{free}} + [\text{Complex}]$$
$$[\text{Guest}]_{\text{total}} = [\text{Guest}]_{\text{free}} + [\text{Complex}]$$

For the titration experiment shown in Figure 5, the host concentration was fixed at **0.53 mM**, while the guest concentration ranged from **0 to 4.09 mM**. Using the measured binding constant $K_a = (2.18 \pm 0.27)\times10^4\ M^{-1}$, the equilibrium concentrations at the titration endpoint (4.09 mM C_{60}) were calculated to be $[\text{Complex}] \approx 0.523\ \text{mM}$, $[\text{Host}]_{\text{free}} \approx 0.007\ \text{mM}$, and $[\text{Guest}]_{\text{free}} \approx 3.567\ \text{mM}$. This corresponds to a **1:1 complex molar fraction of 98.7%** relative to total host, indicating that under the experimental conditions, the host exists almost exclusively as the host–guest complex.

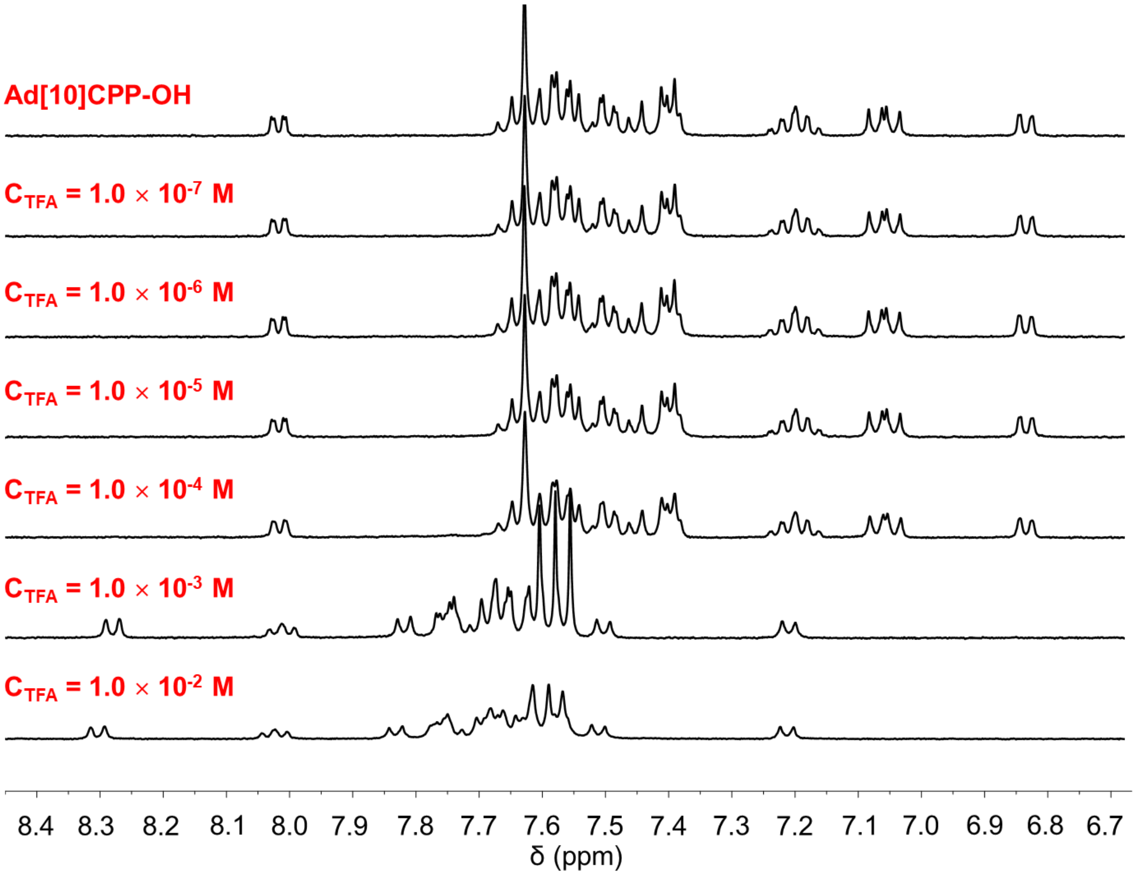

Figure S62. ^{1}H NMR titration experiments. ^{1}H NMR spectra (400 MHz, $C_2D_2Cl_4$-d_2, 298 K) of **Ad[10]CPP-OH** (1.0 mM) without C_{60} titrated by **TFA**, the concentration range of **TFA** is 1.0×10^{-7} M ~ 1.0×10^{-2} M.

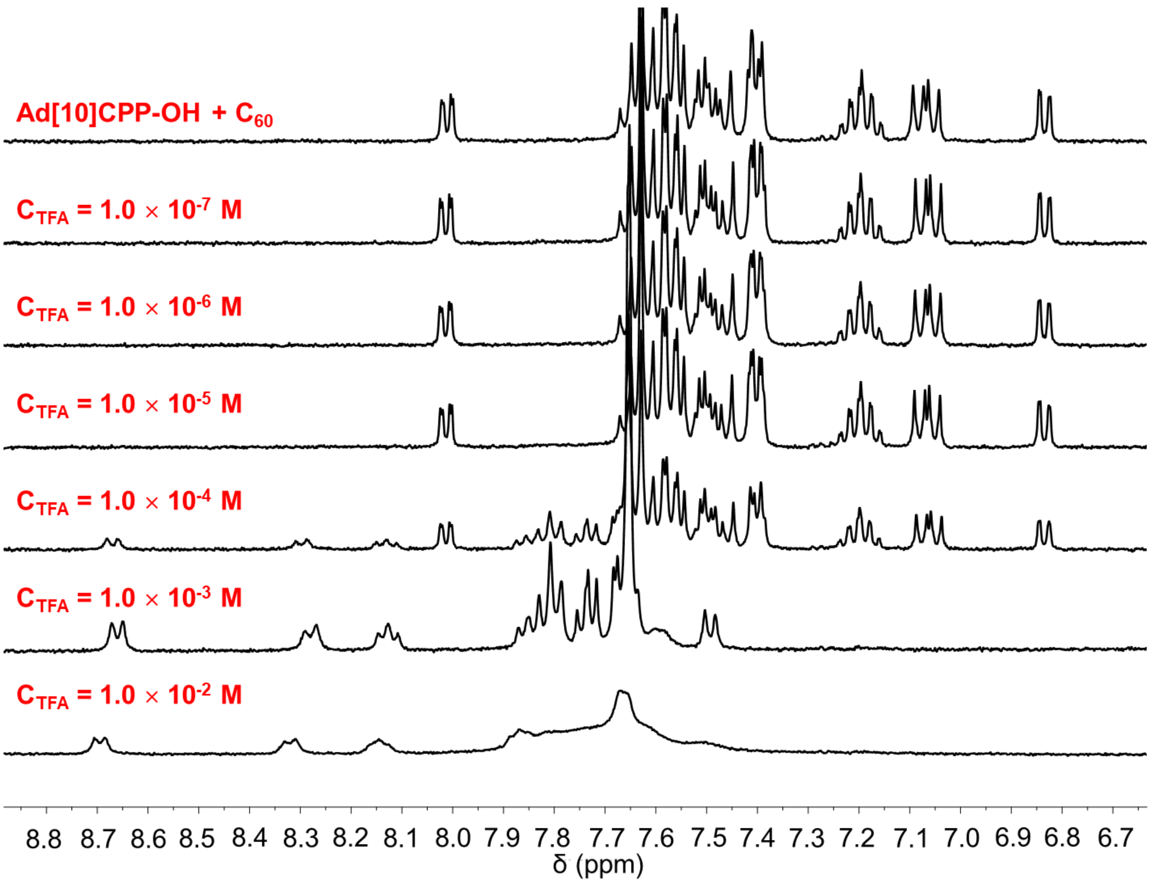

Figure S63. ^{1}H NMR titration experiments. ^{1}H NMR spectra (400 MHz, $C_2D_2Cl_4$-d_2, 298 K) of **Ad[10]CPP-OH** (1.0 mM) with C_{60} (2.4 mM) titrated by **TFA**, the concentration range of **TFA** is 1.0×10^{-7} M ~ 1.0×10^{-2} M.

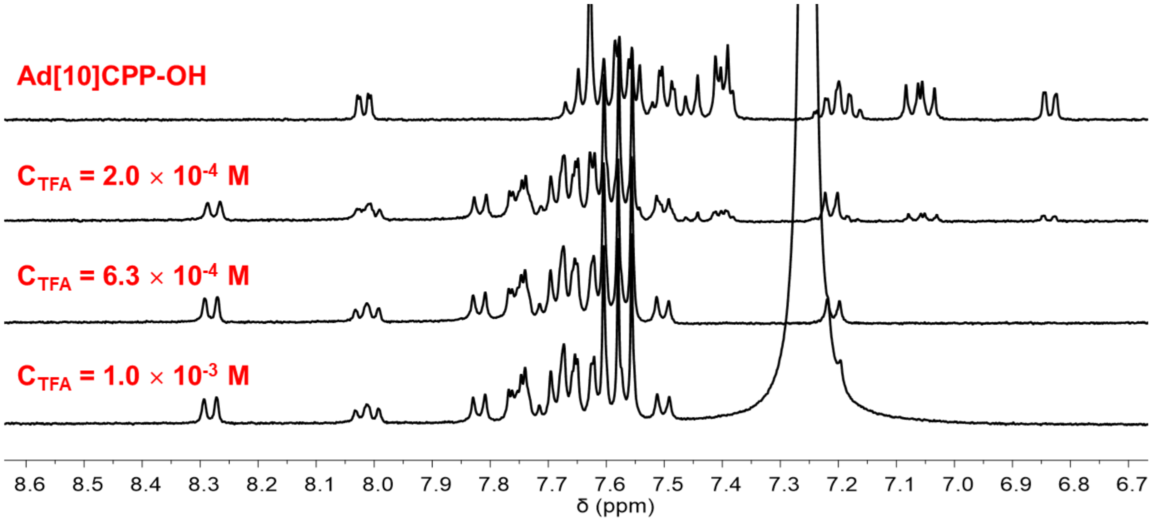

Figure S64. ^{1}H NMR titration experiments. ^{1}H NMR spectra (400 MHz, $C_2D_2Cl_4$-d_2, 298 K) of **Ad[10]CPP-OH** (1.0 mM) without C_{60} titrated by **TFA**, the concentration range of **TFA** is pH = 2.0×10^{-4} M ~ 1.0×10^{-3} M.

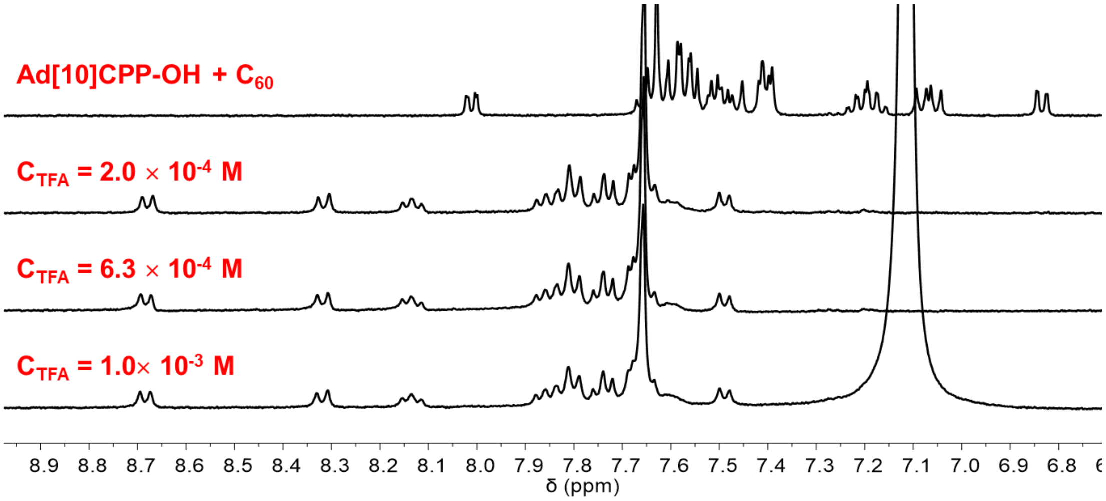

Figure S65. ^{1}H NMR titration experiments. ^{1}H NMR spectra (400 MHz, $C_2D_2Cl_4$-d_2, 298 K) of **Ad[10]CPP-OH** (1.0 mM) with C_{60} (5.0 mM) titrated by **TFA**, the concentration range of **TFA** is 2.0×10^{-4} M ~ 1.0×10^{-3} M.

8. pH-stimulate responsive properties of Ad[10]CPP·TFA

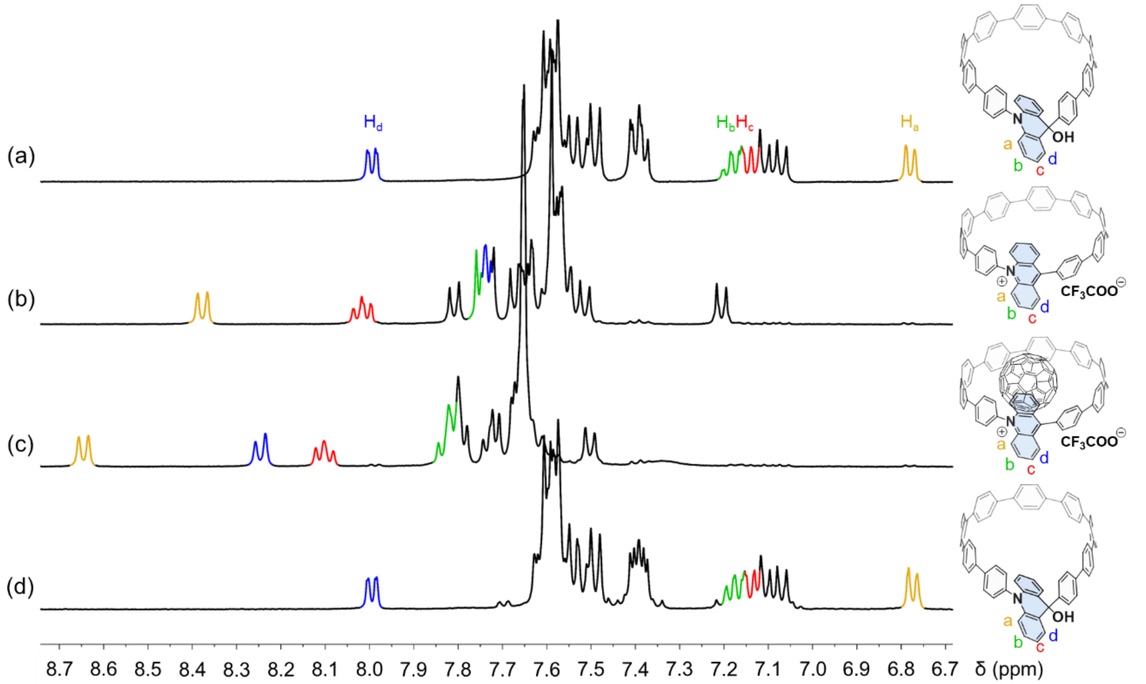

Figure S66. ^{1}H NMR spectra (400MHz, CD_2Cl_2, 298 K) of a) **Ad[10]CPP-OH** and **Ad[10]CPP-OH** with the addition of b) 5 eq TFA, c) 1.5 eq C_{60}, d) 5 eq TEA sequentially.

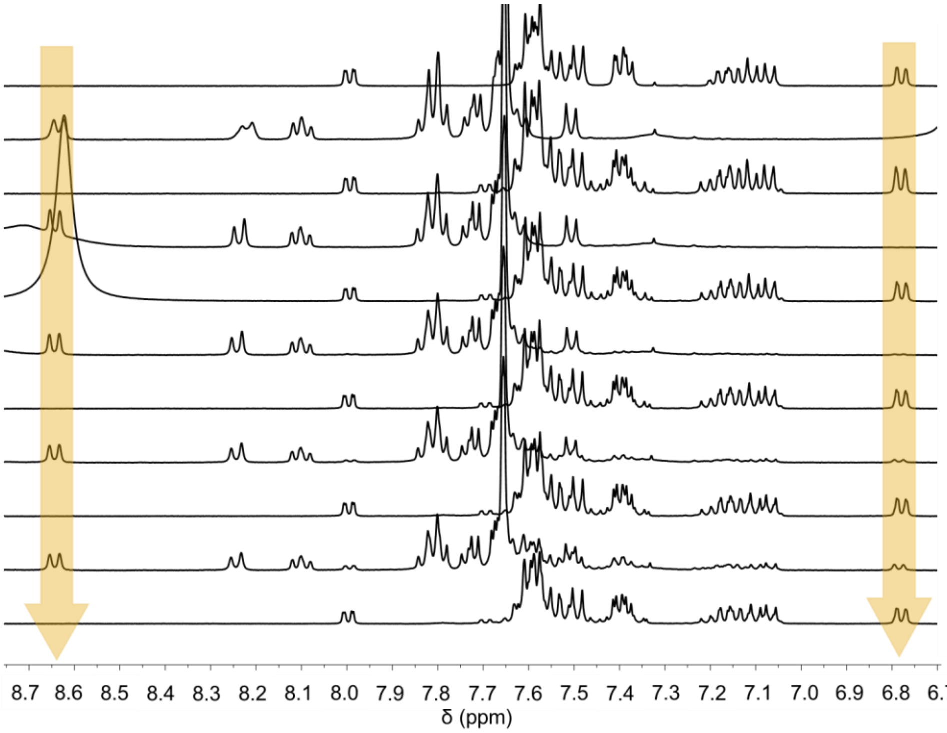

Figure S67. ^{1}H NMR spectra of catch and release C_{60} switching cycles of **Ad[10]CPP·TFA** under Acid (TFA) / neutral (TEA) conditions at least five times.

9. X-ray crystallographic data

The single crystal of **Ad[10]CPP-H** suitable for X-ray crystallographic analysis was obtained by diffusion of pentane into the solution of **Ad[10]CPP-H** in THF at room temperature. The single crystal X-ray diffraction study of **Ad[10]CPP-H** was performed on the Bruker D8 Venture diffractometer using GaKα (λ = 1.34139 Å). The crystal was kept at 213.00 K during data collection. Using Olex2[6], the structure was solved with the SHELXT[7] structure solution program using Intrinsic Phasing and refined with the SHELXL[8]refinement package using Least Squares minimization. Crystallographic data for **Ad[10]CPP-H** reported in this paper have been deposited with the Cambridge Crystallographic Data Centre (CCDC: 2449559).

The single crystal of **Ad[10]CPP-OH** suitable for X-ray crystallographic analysis was obtained by diffusion of pentane into the solution of **Ad[10]CPP-OH** in THF at room temperature. The single crystal X-ray diffraction study of **Ad[10]CPP-H** and **Ad[10]CPP-OH** was performed on the Bruker D8 Venture diffractometer using GaKα (λ = 1.34139 Å). The crystal was kept at 173.00 K during data collection. Using Olex2[5], the structure was solved with the SHELXT[7] structure solution program using Intrinsic Phasing and refined with the SHELXL[8] refinement package using Least Squares minimization. Crystallographic data for **Ad[10]CPP-OH** reported in this paper have been deposited with the Cambridge Crystallographic Data Centre (CCDC: 2449558).

The single crystal of **C_{60}⊂Ad[10]CPP·TFA** suitable for X-ray crystallographic analysis was obtained by diffusion of the solution of **Ad[10]CPP·TFA** in toluene and TFA at room temperature. The single crystal X-ray diffraction study **C_{60}⊂Ad[10]CPP·TFA** was performed on the Bruker D8 Venture diffractometer using GaKα (λ= 1.34139 Å). The crystal was kept at 100.00 K during data collection. Using Olex2[6], the structure was solved with the SHELXT[7] structure solution program using Intrinsic Phasing and refined with the SHELXL[8] refinement package using Least Squares minimization. Crystallographic data for **C60⊂Ad[10]CPP·TFA** reported in this paper have been deposited with the Cambridge Crystallographic Data Centre (CCDC: 2449561).

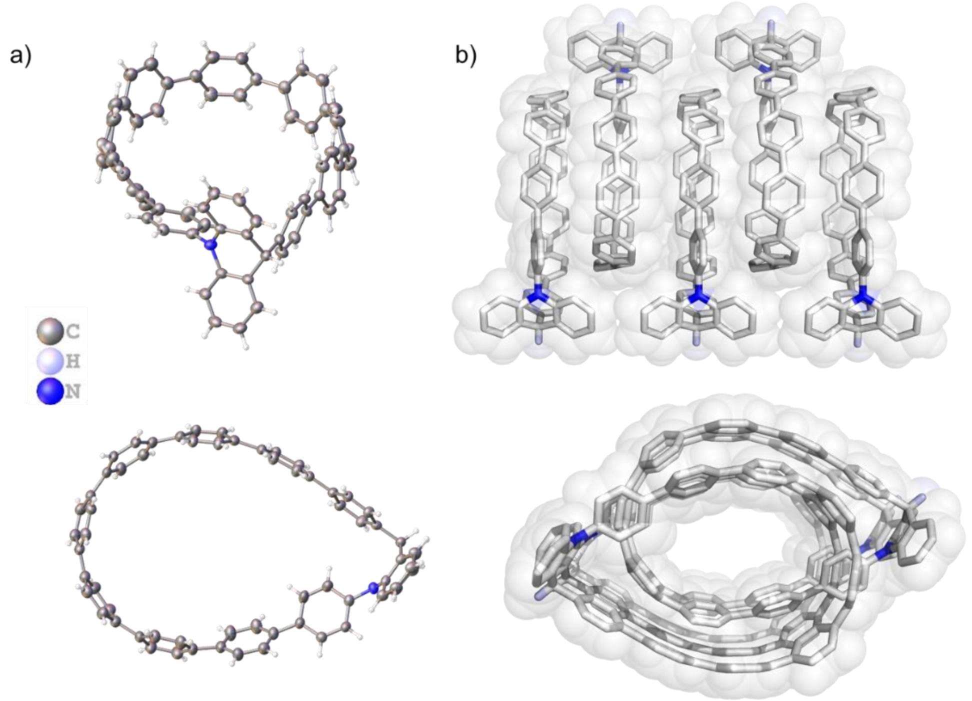

Figure S68. a) ORTEP drawings and b) Packing diagram (space-filling model) of X-ray solid-state structure of **Ad[10]CPP-H** in side view and top view. Thermal ellipsoids are set at 30% probability. Grey C, White H, Blue N. Solvent molecules are omitted for clarity.

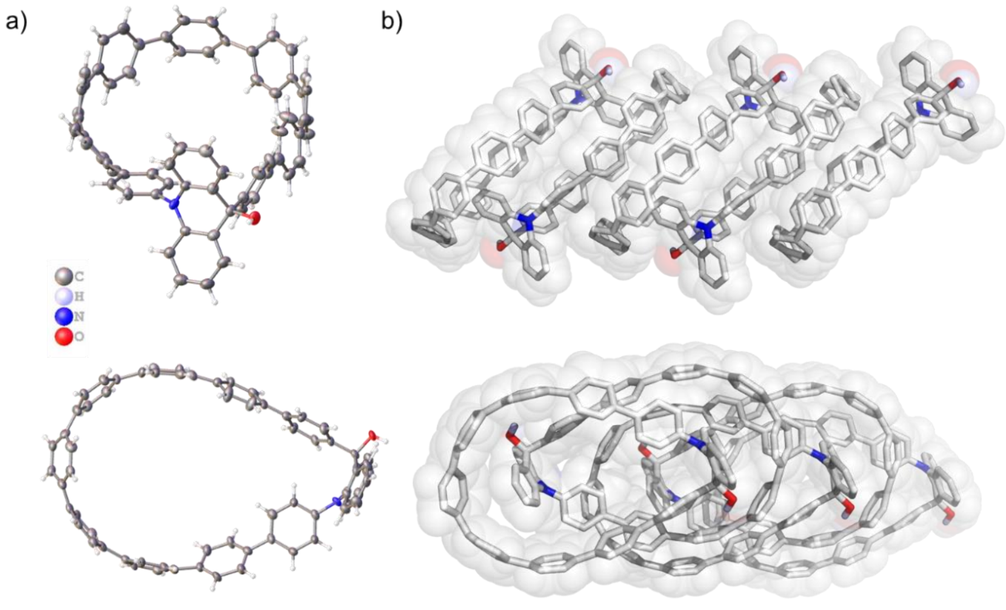

Figure S69. a) ORTEP drawings and b) Packing diagram (space-filling model) of X-ray solid-state structure of **Ad[10]CPP-OH** in side view and top view. Thermal ellipsoids are set at 30% probability. Grey C, White H, Blue N, Red O. Solvent molecules are omitted for clarity.

a)

b)

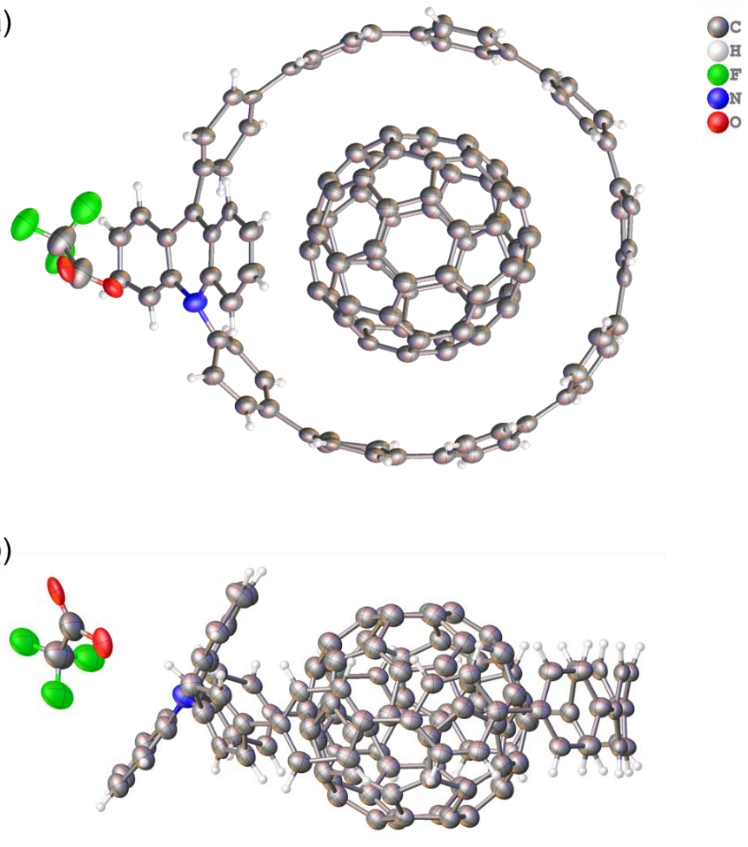

Figure S70. ORTEP drawings of X-ray solid-state structure of **C_{60}⊂ Ad[10]CPP·TFA** in a) top view and b) side view. Thermal ellipsoids are set at 30% probability. grey C, White H, Green F, Blue N, Red O. Solvent molecules are omitted for clarity.

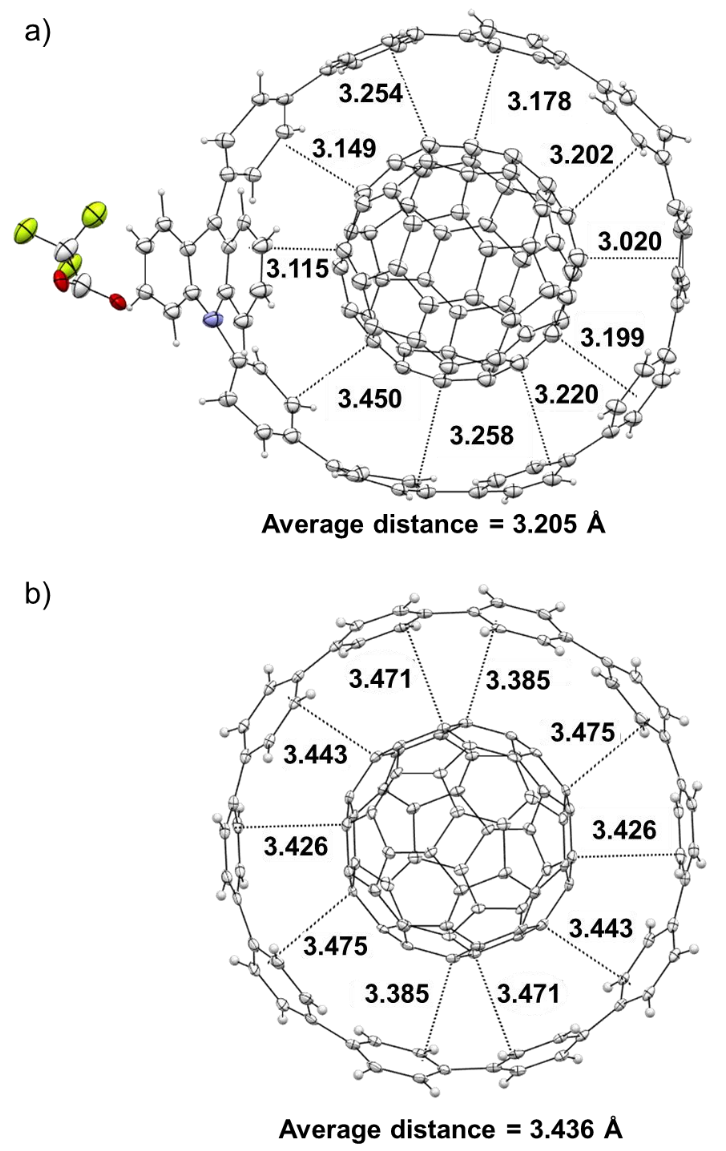

Figure S71. The distance of C_{60} and phenylene moieties in the X-ray solid-state structure of **C_{60}⊂ Ad[10]CPP·TFA** and b) **C_{60}⊂ [10]CPP.** Thermal ellipsoids are set at 15% probability, respectively. Grey C, White H, Blue N. Solvent molecules are omitted for clarity.

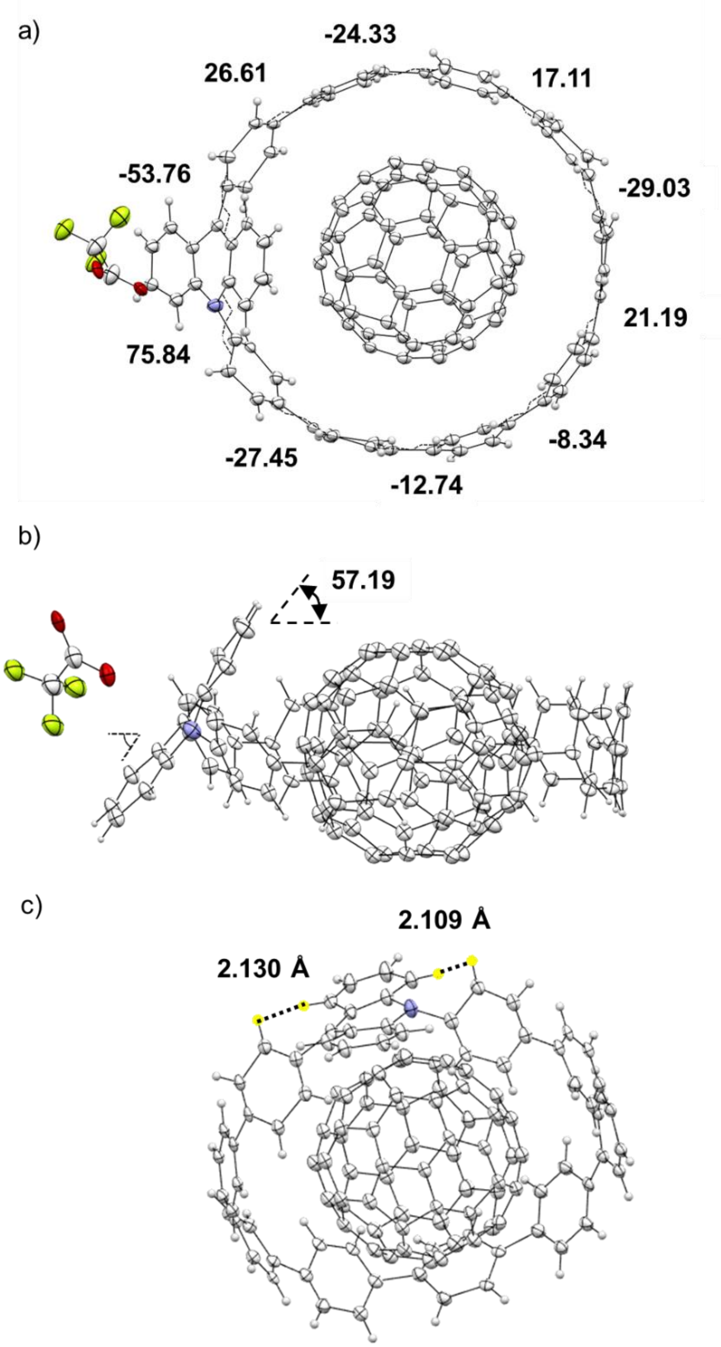

Figure S72. a) The torsion angles of between adjacent phenylene moieties of **Ad[10]CPP**$^{+}$ in the X-ray solid-state structure of **C_{60}⊂ Ad[10]CPP·TFA**, b) The dihedral angle between the acridinium and the ring plane of cycloparaphenylenes of **Ad[10]CPP**$^{+}$ in X-ray solid-state structure of **C_{60}⊂ Ad[10]CPP·TFA**, c) Schematic diagram of steric hindrance between the close contacted hydrogen atoms on acridinium and adjacent phenylene moieties of **Ad[10]CPP**$^{+}$. Thermal ellipsoids are set at 15% probability, respectively. Grey C, White H, Blue N. Solvent molecules are omitted for clarity.

Table S2. Crystal data and structure refinement for Ad[10]CPP-H at 213 K

**Identification code**	**Ad[10]CPP-H**
**Empirical formula**	$C_{73.5}H_{58}ClNO$
**Formula weight**	1006.66
**Temperature/K**	213.00
**Crystal system**	triclinic
**Space group**	P-1
**a/Å**	10.
**b/Å**	17.7237(15)
**c/Å**	19.0853(17)
**α/°**	113.419(6)
**β/°**	92.807(7)
**γ/°**	104.671(6)
**Volume/Å^3**	3135.1(5)
**Z**	2
**ρ_{calc}g/cm^3**	1.066
**μ/mm^{-1}**	0.555
**F (000)**	1062.0
**Crystal size/mm^3**	0.07 × 0.07 × 0.05
**Radiation**	GaKα (λ = 1.34139)
**2Θ range for data collection/°**	7.614 to 110.286
**Index ranges**	$-12 \leq h \leq 12$, $-21 \leq k \leq 21$, $-23 \leq l \leq 23$
**Reflections collected**	37383
**Independent reflections**	11912 [R_{int} = 0.1230, R_{sigma} = 0.1470]
**Data/restraints/parameters**	11912/166/714
**Goodness-of-fit on F^2**	0.834
**Final R indexes [I>=2σ (I)]**	R_1 = 0.1192, wR_2 = 0.2676
**Final R indexes [all data]**	R_1 = 0.2134, wR_2 = 0.3215
**Largest diff. peak/hole / e Å^{-3}**	0.77/-0.42

Table S3. Crystal data and structure refinement for Ad[10]CPP-OH at 173 K

**Identification code**	**Ad[10]CPP-OH**
**Empirical formula**	$C_{71}H_{53}NO_2$
**Formula weight**	952.14
**Temperature/K**	173.00
**Crystal system**	triclinic
**Space group**	P-1
**a/Å**	12.6507(8)
**b/Å**	17.9247(11)
**c/Å**	25.9006(19)
**α/°**	79.650(4)
**β/°**	79.495(4)
**γ/°**	73.761(4)
**Volume/Å^3**	5492.6(6)
**Z**	4
**ρ_{calc}g/cm^3**	1.151
**μ/mm^{-1}**	0.334
**F (000)**	2008.0
**Crystal size/mm^3**	0.17 × 0.17 × 0.05
**Radiation**	GaKα (λ = 1.34139)
**2Θ range for data collection/°**	5.086 to 110.696
**Index ranges**	$-14 \leq h \leq 15$, $-21 \leq k \leq 21$, $-31 \leq l \leq 29$
**Reflections collected**	45042
**Independent reflections**	20364 [R_{int} = 0.1584, R_{sigma} = 0.2025]
**Data/restraints/parameters**	20364/19/1335
**Goodness-of-fit on F^2**	0.831
**Final R indexes [I>=2σ (I)]**	R_1 = 0.1515, wR_2 = 0.2959
**Final R indexes [all data]**	R_1 = 0.2425, wR_2 = 0.3501
**Largest diff. peak/hole / e Å^{-3}**	0.44/-0.45

Table S4. Crystal data and structure refinement for C_{60}⊂Ad[10]CPP·TFA at 100 K

**Identification code**	**C_{60}⊂Ad[10]CPP·TFA**
**Empirical formula**	$C_{322}H_{156}F_{18}N_2O_{12}$
**Formula weight**	4586.48
**Temperature/K**	100.00
**Crystal system**	triclinic
**Space group**	P1
**a/Å**	9.8726(18)
**b/Å**	17.269(3)
**c/Å**	31.681(5)
**α/°**	78.618(8)
**β/°**	89.112(8)
**γ/°**	78.742(8)
**Volume/Å^3**	5191.7(16)
**Z**	1
**ρ_{calc}g/cm^3**	1.466
**μ/mm^{-1}**	0.515
**F(000)**	2360.0
**Crystal size/mm^3**	0.3 × 0.03 × 0.03
**Radiation**	GaKα (λ = 1.34139)
**2Θ range for data collection/°**	2.476 to 75.13
**Index ranges**	$-8 \leq h \leq 8$, $-15 \leq k \leq 15$, $-28 \leq l \leq 28$
**Reflections collected**	41467
**Independent reflections**	14490 [R_{int} = 0.0961, R_{sigma} = 0.1505]
**Data/restraints/parameters**	14490/11258/3215
**Goodness-of-fit on F^2**	1.530
**Final R indexes [I>=2σ (I)]**	R_1 = 0.1476, wR_2 = 0.3683
**Final R indexes [all data]**	R_1 = 0.2184, wR_2 = 0.4298
**Largest diff. peak/hole / e Å^{-3}**	0.44/-0.42
**Flack parameter**	0.5(9)

10. Theoretical Calculations

Ring strain energies are estimated by hypothetical reactions as shown below[9] (**Figure S72**). In this way, considering many possible conformations of each long ring opening counterpart as reference can be avoided. The heat of formation (ΔH) of the optimized structures of the reference compounds were calculated to obtain the ring strain energies of **Ad[10]CPP-H**, **Ad[10]CPP-OH** and **Ad[10]CPP**$^+$ (**Table S5**).

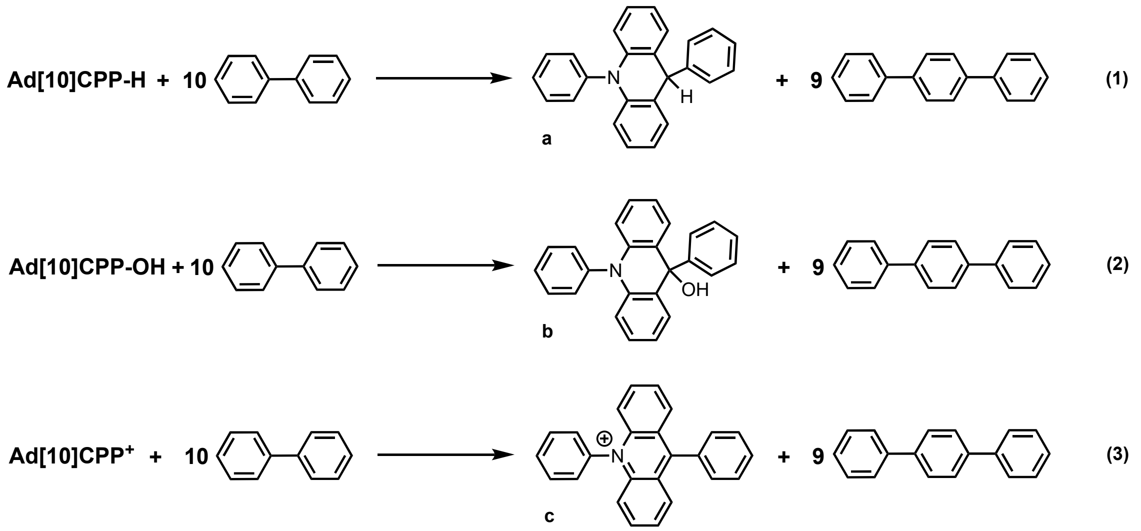

Figure S73. Hypothetical reactions for the calculation of strain energies of **Ad[10]CPP-H**, **Ad[10]CPP-OH** and **Ad[10]CPP**$^+$.

Table S5. Electronic energies (E_e), relative energies (ΔE), and strain energies (ΔH) of **Ad[10]CPP-H**, **Ad[10]CPP-OH** and **Ad[10]CPP**$^+$ obtained at the B3LYP-D3(BJ)/def2-TZVP-IEFPCM(Dichloromethane)/B3LYP-D3(BJ)/def2-SVP level of theory.

	E_e (a.u.)	ΔE (kcal/mol)	ΔH (kcal/mol)
Biphenyl	-463.522649		
p-terphenyl	-694.687904		
a	-1019.350484		
b	-1094.605215		
c	-1018.585656		
**Ad[10]CPP-H**	-2636.250092	40.8	38.8
**Ad[10]CPP-OH**	-2711.504635	40.9	38.7
**Ad[10]CPP**$^+$	-2635.456602	58.8	56.2

Gaussian 16 package[10] was applied to perform all the DFT calculations. Optimizations of the geometries were carried out with B3LYP-D3 functional with Becke-Johnson (BJ) damping function[11, 12] and def2-SVP basis set[13, 14]. For the radical species, the optimization was conducted with unrestricted UB3LYP functional. IEFPCM solvation model[15] was used to model the solvation effects of dichloromethane/acetonitrile, while the single-point energies were evaluated at B3LYP-D3(BJ)/def2-TZVP level of theory. The DFT optimized structures are illustrated using VMD[16]. The spin density analysis was performed by using Multwfn[17]. The photophysical property of the macrocycles were studied using TDDFT at the CAM-B3LYP/ def2-SVP level of theory[18], and the solvation effect was taken into consideration for both ground-state and excited state calculations using IEFPCM solvation model. Larger basis set def2-SVP were tested as well. As noticed by Jacquemin *et al.*[19], calculation with basis set larger than 6-31+G(d) only changes the relative intensities by a trifling amount, we found that the adsorption and emission spectrum obtained through def2-SVP and def2-SVP is not significantly different. Hence the lower cost def2-SVP basis set was used. Conformational search was conducted with xTB and CREST programs[20, 21]. All reported bond lengths are in Ångstroms (Å).

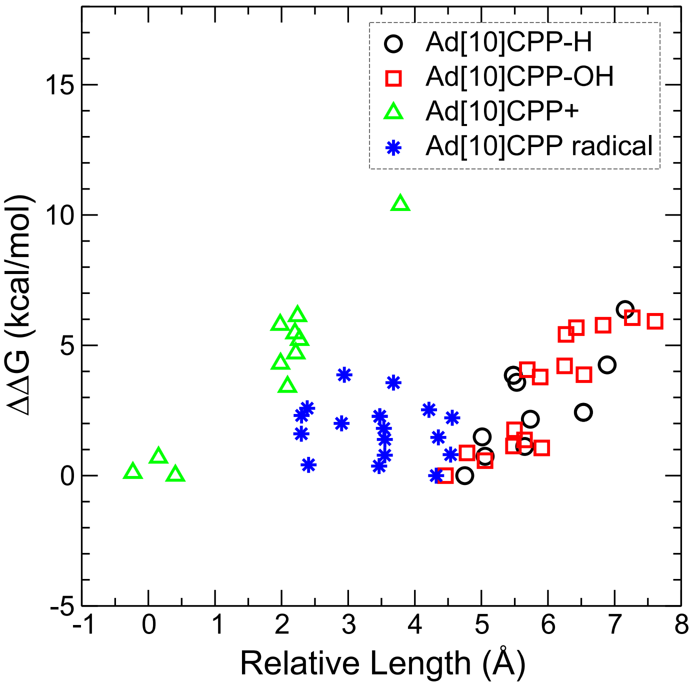

Figure S74. The length of long axis relative to short axis versus the free energy relative to that of the lowest conformation. Different conformations of **Ad[10]CPP-H** (black circle), **Ad[10]CPP-OH** (red square), **Ad[10]CPP$^+$** cation (green triangle) and **Ad[10]CPP$^•$**, that have been calculated.

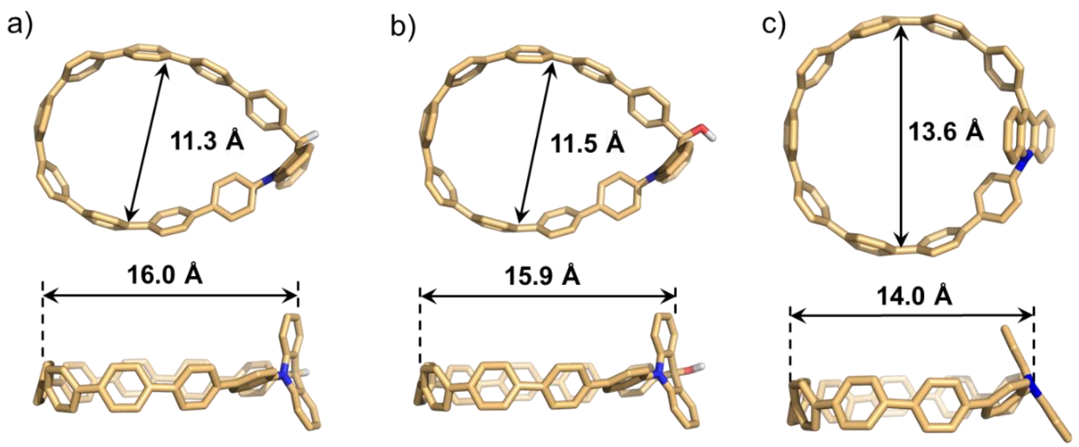

Figure S75. The DFT optimized structures of a) **Ad[10]CPP-H**, b) **Ad[10]CPP-OH**, and c) **Ad[10]CPP$^+$,** at B3LYP-D3(BJ)/def2-SVP level of theory.

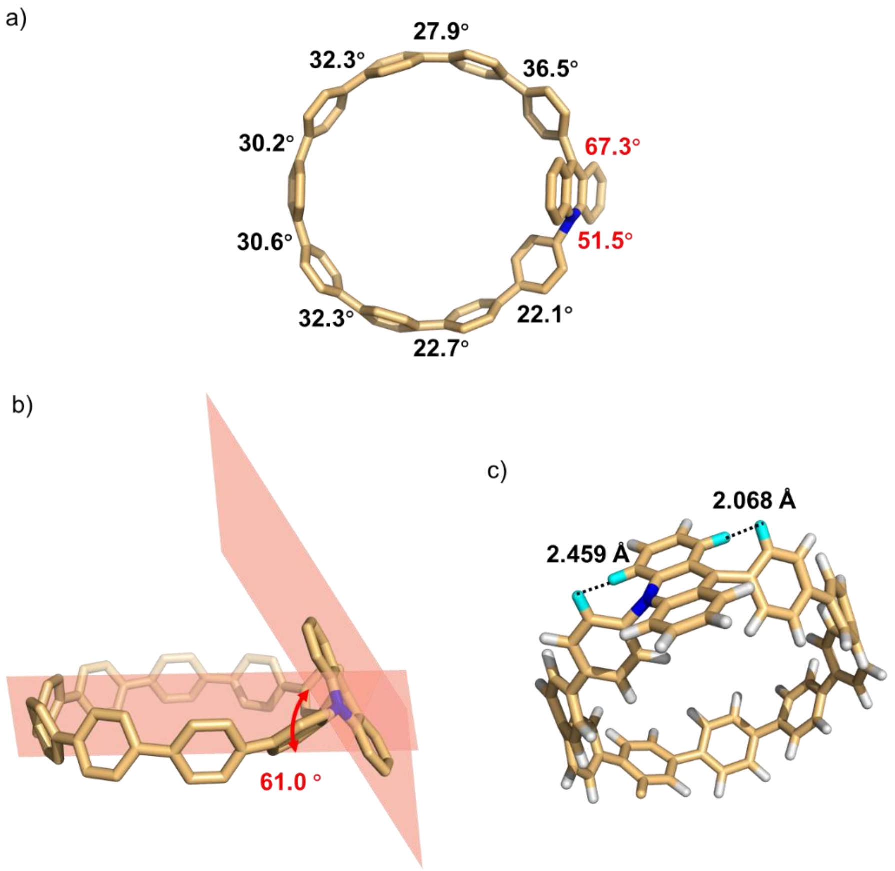

Figure S76. a) The torsion angles between the acridinium and adjacent phenylene moieties of **Ad[10]CPP$^+$**, b) The dihedral angle between the acridinium and the ring plane of cycloparaphenylenes of **Ad[10]CPP$^+$**, c) Schematic diagram of steric hindrance between the close contacted hydrogen atoms on acridinium and adjacent phenylene moieties of **Ad[10]CPP$^+$** These structures are energetically favorable at B3LYP-D3(BJ)/def2-SVP level of theory.

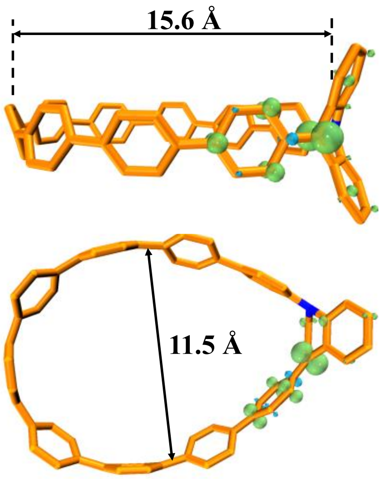

Figure S77. The DFT optimized structures of **Ad[10]CPP˙**, and its spin density plot. The α - and β-spin densities at B3LYP-D3(BJ)/def2-SVP level of theory are in green and blue, respectively.

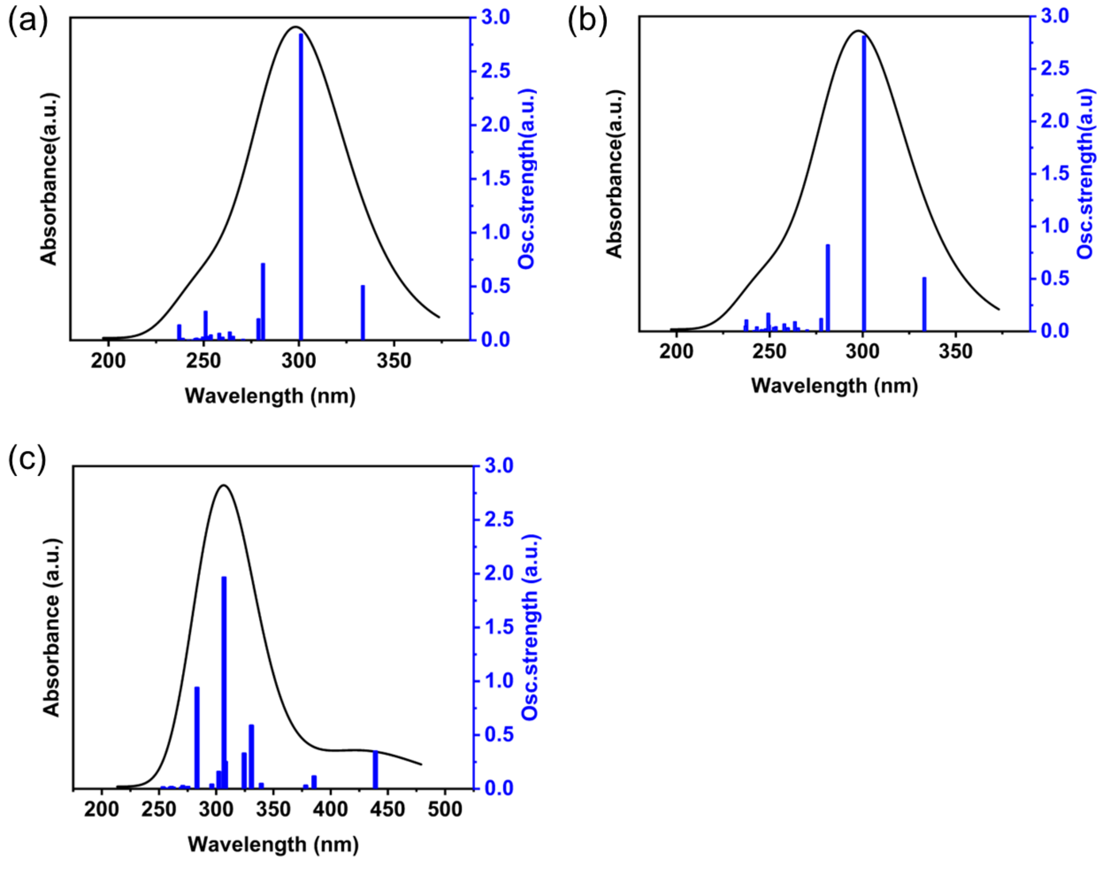

Figure S78. The UV-vis absorption spectra of DFT optimized structures of a) **Ad[10]CPP-H**, b) **Ad[10]CPP-OH**, c) **Ad[10]CPP^{+}** at CAM-B3LYP/def2-SVP-IEFPCM(Dichloromethane) level of theory.

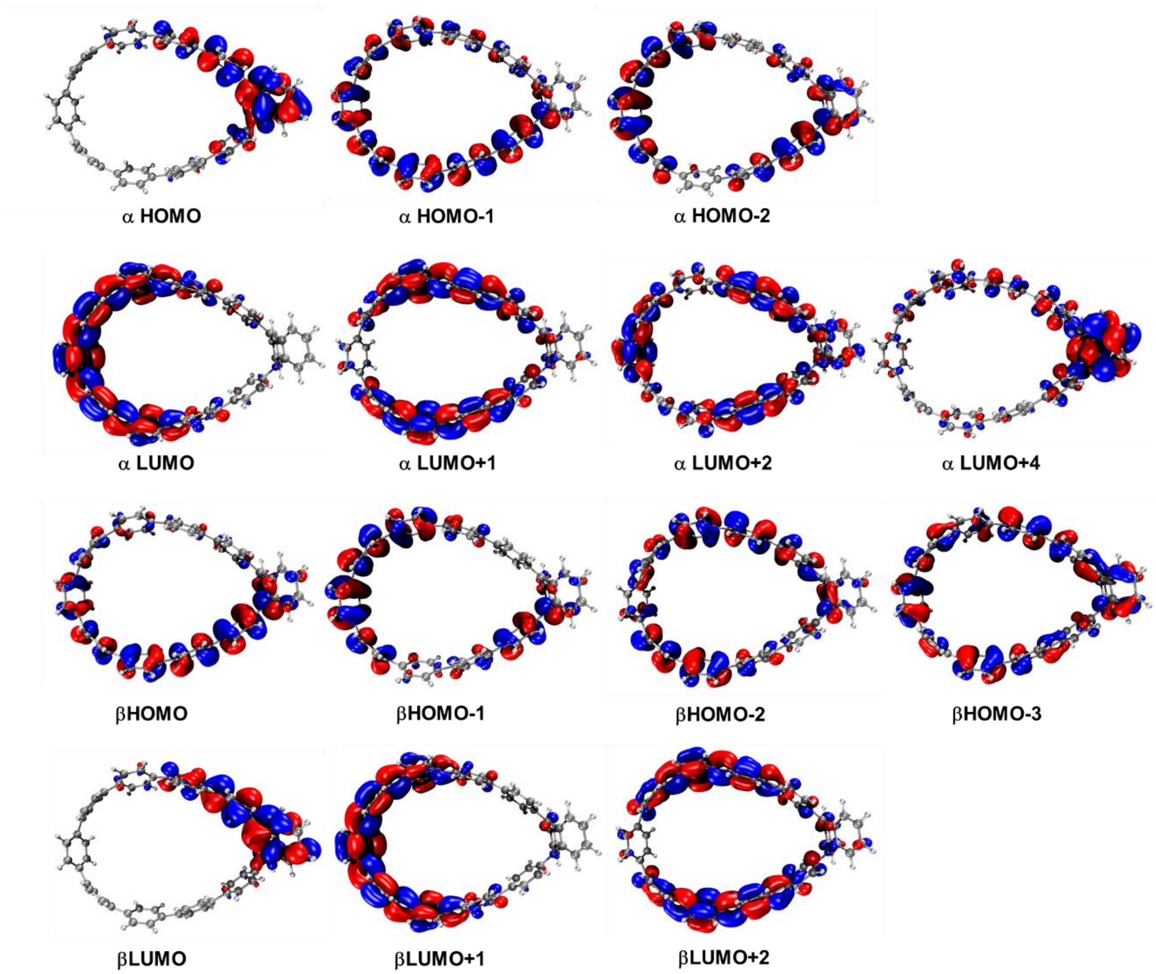

Figure S79. The frontier molecular orbital of **Ad[10]CPP·** in ground states at CAM-B3LYP/def2-SVP-IEFPCM(Dichloromethane) level of theory.

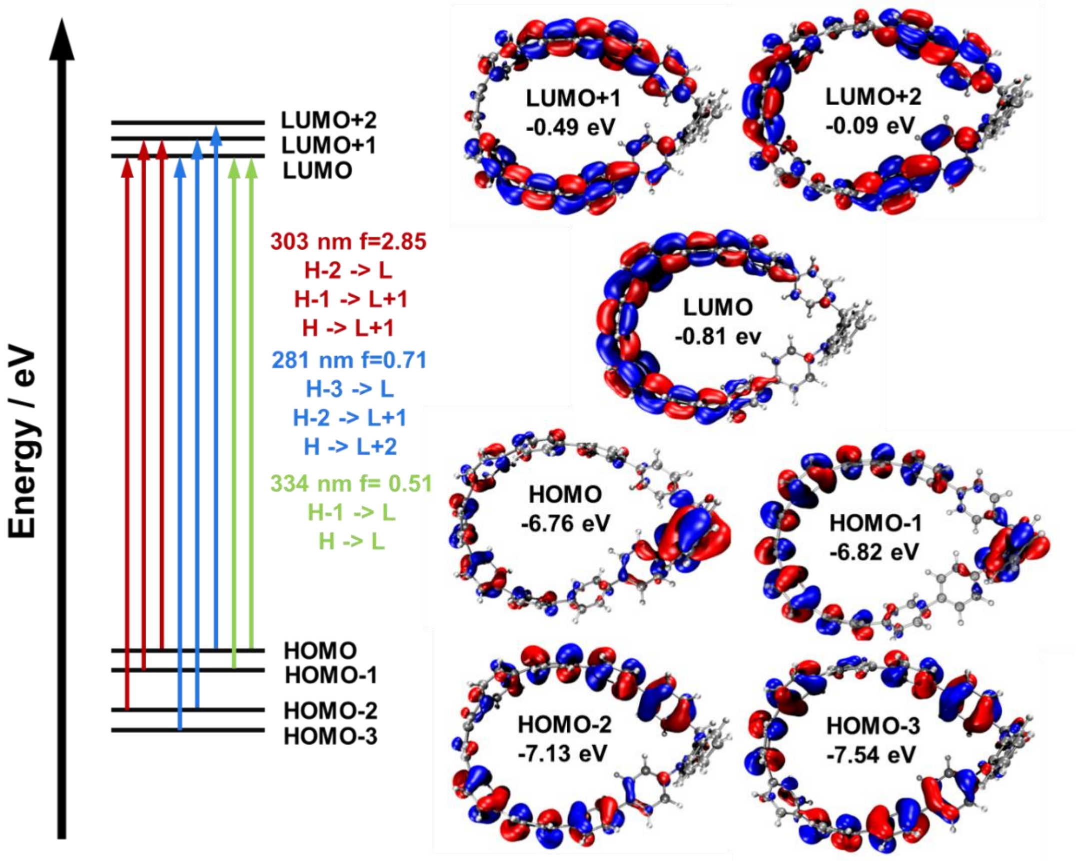

Figure S80. The frontier molecular orbitals and LUMO-HOMO gaps for **Ad[10]CPP-H** at the optimized geometries of S_0 state calculated by DFT at the CAM-B3LYP/def2-SVP-IEFPCM(Dichloromethane) level.

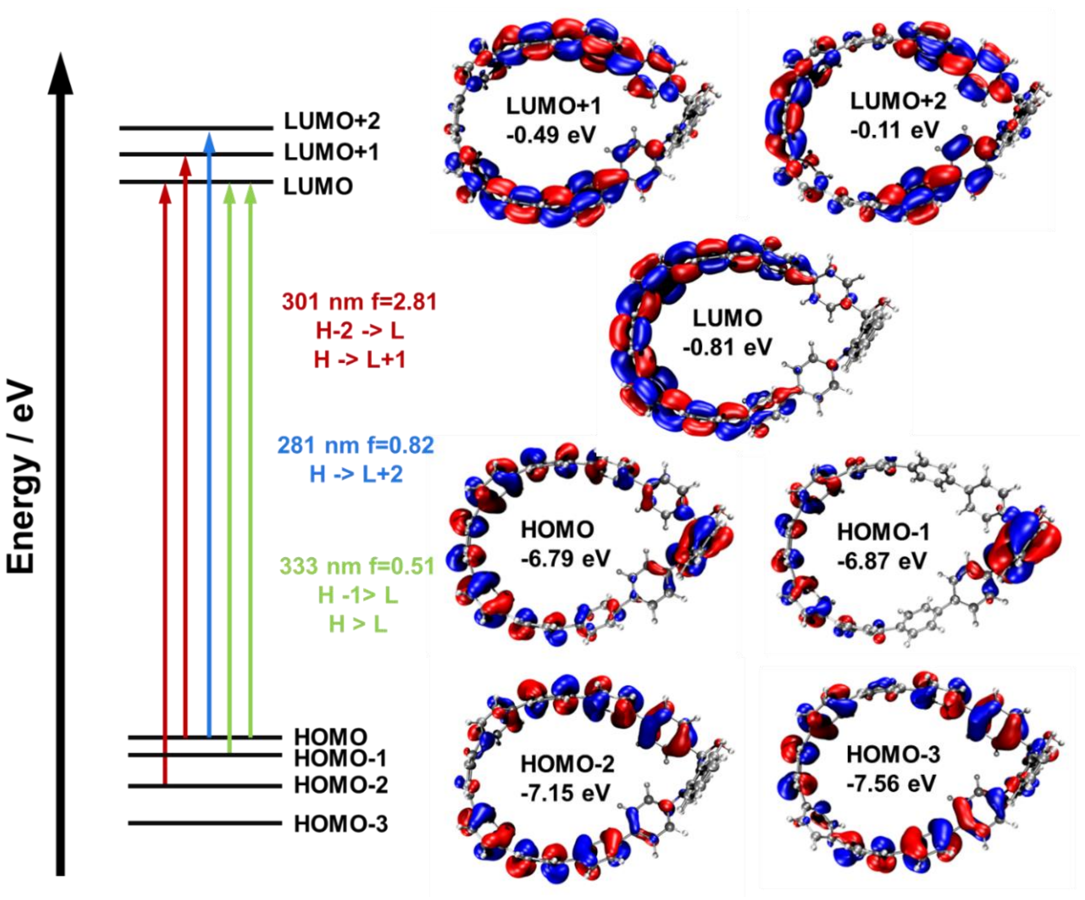

Figure S81. The frontier molecular orbitals and LUMO-HOMO gaps for **Ad[10]CPP-OH** at the optimized geometries of S_0 state calculated by DFT at the CAM-B3LYP/def2-SVP-IEFPCM (Dichloromethane) level.

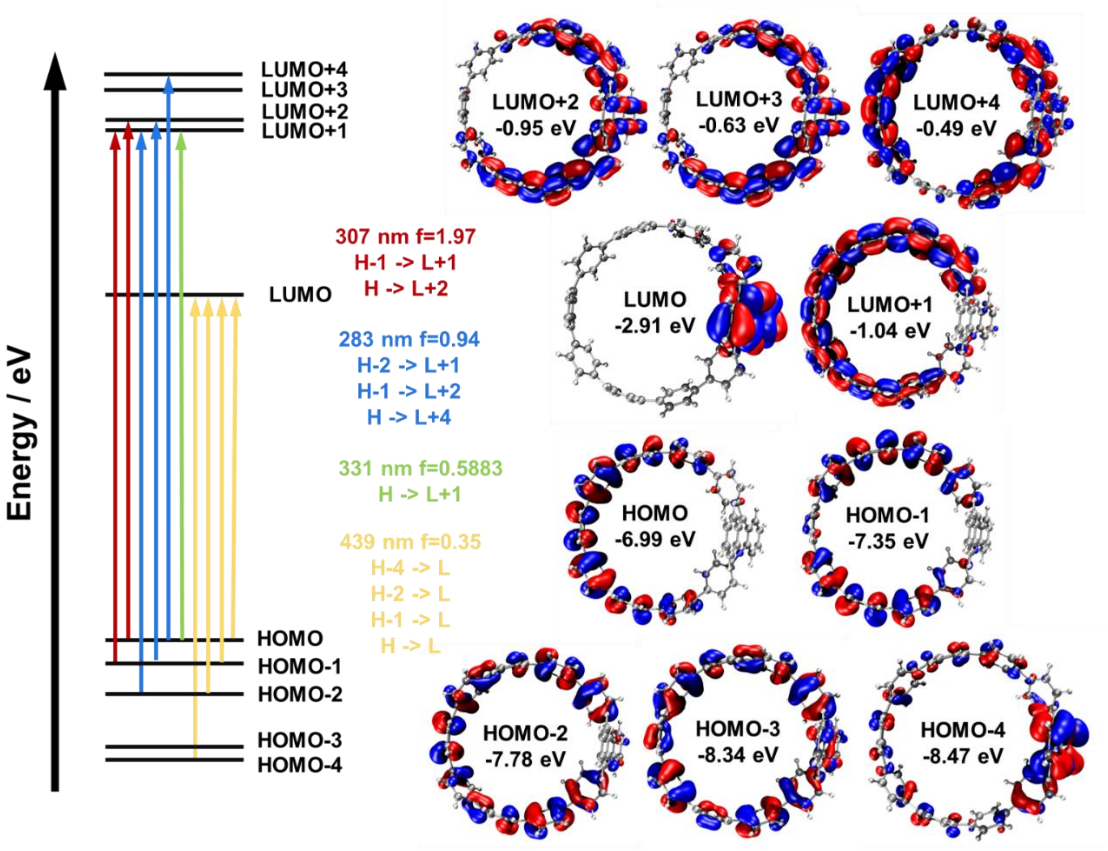

Figure S82. The frontier molecular orbitals and LUMO-HOMO gaps for **Ad[10]CPP**$^+$ at the optimized geometries of S_0 state calculated by DFT at the CAM-B3LYP/def2-SVP-IEFPCM(Dichloromethane) level.

Table S6. Oscillator strengths (> 0.01) of Ad[10]CPP-H by applying TD-DFT calculations at the level of CAM-B3LYP/def2-SVP-IEFPCM (Dichloromethane).

Excited state	Energy (eV)	Wavelength (nm)	Osc. Strength	Major contributions	
1	3.7166	333.6	0.5059	H-1 -> L	50.7%
				H -> L	21.6%
2	4.1174	301.13	2.8455	H-2 -> L	38.0%
				H-1 -> L+1	22.3%
				H -> L+1	15.7%
3	4.4096	281.17	0.7123	H-3 -> L	15.1%
				H-2 -> L+1	16.3%
				H -> L+2	22.5%
4	4.4476	278.76	0.1978	H -> L+3	28.3%
6	4.6705	265.46	0.0339	H-1 -> L+5	20.8%
7	4.7018	263.69	0.0747	H -> L+4	23.5%
				H -> L+5	15.1%
12	4.9395	251	0.2672	H -> L+11	17.2%
				H -> L+12	28.9%
20	5.2278	237.16	0.141	H-3 -> L+2	16.7%

Table S7. Oscillator strengths (> 0.01) of Ad[10]CPP-OH by applying TD-DFT calculations at the level of CAM-B3LYP/def2-SVP-IEFPCM(Dichloromethane).

Excited state	Energy (eV)	Wavelength (nm)	Osc. Strength	Major contributions	
1	3.7215	333.15	0.5113	H-1 -> L	16.1%
				H -> L	56.3%
2	4.1229	300.72	2.8106	H-2 -> L	38.1%
				H -> L+1	34.0%
3	4.4081	281.27	0.8226	H -> L+2	22.7%
4	4.4649	277.69	0.1207	H-1 -> L+1	21.2%
				H-1 -> L+3	23.8%
5	4.5897	270.14	0.013	H-1 -> L+2	29.8%
6	4.6746	265.23	0.031	H -> L+5	26.8%
7	4.7038	263.58	0.0905	H-1 -> L+4	38.4%
13	4.9745	249.24	0.1711	H-1 -> L+10	23.2%

Table S8. Oscillator strengths (> 0.01) of Ad[10]CPP cation by applying TD-DFT calculations at the level of CAM-B3LYP/def2-SVP-IEFPCM(Dichloromethane).

Excited state	Energy (eV)	Wavelength (nm)	Osc. Strength	Major contributions	
1	2.8241	439.02	0.3484	H-4 -> L	15.3%
				H-2 -> L	24.9%
				H-1 -> L	16.7%
				H -> L	24.7%
2	3.2167	385.44	0.1154	H-4 -> L	55.0%
				H-1 -> L	22.9%
3	3.2786	378.16	0.0293	H-1 -> L	31.6%
				H -> L	49.4%
4	3.6538	339.33	0.0463	H-2 -> L	41.8%
				H -> L	18.8%
5	3.7492	330.69	0.5883	H -> L+1	56.8%
6	3.8224	324.36	0.3285	H-16 -> L	39.9%
				H-13 -> L	38.0%
7	4.0246	308.07	0.2501	H-14 -> L	62.8%
8	4.0412	306.8	1.9651	H-1 -> L+1	35.8%
				H -> L+2	24.1%
9	4.103	302.18	0.1575	H-16 -> L	31.0%
				H-3 -> L	16.1%
10	4.1868	296.13	0.0401	H-15 -> L	17.1%
				H-3 -> L	24.0%
11	4.3792	283.12	0.9401	H-2 -> L+1	25.5%
				H-1 -> L+2	20.7%
				H -> L+4	27.0%
12	4.5056	275.18	0.0188	H-15 -> L	48.9%
13	4.5795	270.74	0.0275	H-18 -> L	15.4%
				H-12 -> L	19.1%

Table S9. Oscillator strengths (> 0.01) of Ad[10]CPP$^{\bullet}$ by applying TD-DFT calculations at the level of CAM-B3LYP/def2-SVP-IEFPCM(Dichloromethane).

Excited state	Energy (eV)	Wavelength (nm)	Osc. Strength	Major contributions
2	2.5350	489.09	0.01170	Hβ-2 -> Lβ 13.6%<br>Hβ -> Lβ 12.8%,<br>Hα-3 -> Lα 5.6%

				Hα-1 -> Lα+1 5.5%
3	2.5939	477.98	0.02080	Hβ -> Lβ 17.8%<br>Hβ -> Lβ+2 10.5%<br>Hβ-1 -> Lβ 7.1%<br>Hα-1 -> Lα+1 6.6%
4	2.8176	440.03	0.06110	Hβ -> Lβ 19.8%<br>Hβ-1 -> Lβ 11.9%<br>Hβ-3 -> Lβ 5.9%,<br>Hα-1 -> Lα+2 5.5%<br>Hα -> Lα+1 5.3%
6	3.1095	398.73	0.07280	Hα -> Lα+3 9.4%<br>Hα -> Lα+1 5.8%
9	3.3965	365.04	0.37400	Hβ-3 -> Lβ 8.4%<br>Hβ-2 -> Lβ 7.2%<br>Hα -> Lα+1 6.7%
10	3.4898	355.28	0.34630	Hβ-1 -> Lβ 5.9%
11	3.5472	349.53	0.02650	Hβ-9 -> Lβ 16.8%<br>Hα -> Lα+6 13.4%<br>Hβ-14 -> Lβ 11.7%<br>Hα -> Lα+14 8.5%<br>Hα -> Lα+4 6.0%<br>Hα -> Lα+5 5.0%
13	3.7145	333.78	0.23670	Hα -> Lα+4 20.7%<br>Hα -> Lα+2 5.5%
14	3.7518	330.47	0.85510	Hα-1 -> Lα 13.1%<br>Hβ -> Lβ+1 10.5%<br>Hβ-1 -> Lβ+1 8.4%<br>Hα-2 -> Lα 5.7%
15	3.8037	325.96	0.23130	Hα -> Lα+4 10.5%
16	3.8221	324.39	0.23860	Hα -> Lα+4 12.2%
17	3.8517	321.89	0.03630	Hα -> Lα+7 19.9%<br>Hα-1 -> Lα+7 8.3%<br>Hβ -> Lβ+8 6.2%
				Hβ-5 -> Lβ 5.1%
18	3.9094	317.14	0.01700	Hβ-5 -> Lβ 28.2%<br>Hβ-11 -> Lβ 15.7%<br>Hα -> Lα+6 6.9%
19	3.9633	312.83	0.06260	Hβ-11 -> Lβ 11.8%<br>Hα -> Lα+6 6.7%<br>Hβ-10 -> Lβ 5.7%

				Hβ-14 -> Lβ 5.3%
20	4.0280	307.81	1.66060	Hβ -> Lβ+2 11.5%<br>Hα-1 -> Lα+1 10.7%<br>Hα -> Lα 9.7%

Cartesian coordinates of optimized structures

Ad[10]CPP-H

C	-8.46637900	-0.04352900	1.32995300
C	-8.25703000	1.33153100	1.32259300
C	-8.38998300	2.08143400	0.13881400
C	-8.97780000	1.43015300	-0.96453300
C	-9.18832900	0.05334200	-0.95695900
C	-8.81856800	-0.73216000	0.15377900
H	-8.19381100	-0.60672700	2.22409800
H	-7.82939800	1.79769900	2.21183800
H	-9.19184700	1.99503600	-1.87477000
H	-9.56164900	-0.43131400	-1.86205700
C	-7.61605300	3.34021100	0.00608900
C	-6.92650200	3.58558700	-1.19671600
C	-7.26682300	4.13478700	1.11631600
C	-5.81483700	4.42199300	-1.23324300
H	-7.17091400	3.00249900	-2.08629400
C	-6.15155100	4.96801300	1.08115600
H	-7.83106800	4.04130500	2.04698300
C	-5.33963900	5.04980100	-0.06751500
H	-5.22592600	4.47330400	-2.15104100
H	-5.86501200	5.51324800	1.98334700
C	-8.43460100	-2.16007700	0.03032300
C	-7.84178600	-2.59170700	-1.17138700
C	-8.30546300	-3.01236400	1.14569700
C	-6.97727800	-3.68090100	-1.19685600
H	-7.93027800	-1.97604200	-2.06803000
C	-7.43868100	-4.10248600	1.12030300
H	-8.82439500	-2.76865800	2.07559500
C	-6.66517900	-4.39054100	-0.02220300
H	-6.42125200	-3.88260700	-2.11392400
H	-7.29979400	-4.69126000	2.02991100
C	-5.38038500	-5.12919000	0.04580800
C	-4.59968800	-5.03611400	1.21375200
C	-4.75934700	-5.66875700	-1.09814800

C	-3.23086800	-5.28866200	1.18991300
H	-5.04084200	-4.62993200	2.12559000
C	-3.38861100	-5.91265600	-1.12501200
H	-5.34635900	-5.83556500	-2.00396200
C	-2.57902300	-5.64091100	-0.00523600
H	-2.63991000	-5.08041000	2.08414900
H	-2.92691100	-6.26660100	-2.04968000
C	-3.92927300	5.50318300	-0.01197500
C	-3.23681400	5.98675200	-1.13846900
C	-3.16597300	5.20993300	1.13350800
C	-1.84461300	6.03939800	-1.16167800
H	-3.79624800	6.28640900	-2.02765300
C	-1.77620400	5.25896500	1.10838600
H	-3.66284800	4.82510000	2.02590100
C	-1.07704800	5.60775700	-0.06195000
H	-1.34233600	6.38776000	-2.06663000
H	-1.22477600	4.91576900	1.98519500
C	-1.11058400	-5.46540100	-0.11509200
C	-0.22965200	-5.70526500	0.95523800
C	-0.58785800	-4.82938600	-1.25699400
C	1.08307700	-5.23324100	0.92790900
H	-0.59139300	-6.22866800	1.84320800
C	0.71752800	-4.35001700	-1.28042100
H	-1.24253800	-4.63204000	-2.10795000
C	1.56381800	-4.49406600	-0.16728100
H	1.72380000	-5.37714600	1.80119900
H	1.07748700	-3.81578000	-2.16235800
C	0.37831500	5.34204100	-0.16044600
C	0.94873000	4.92801600	-1.37910500
C	1.20954400	5.31942700	0.97511400
C	2.24623100	4.42711900	-1.43986500
H	0.34375900	4.93488800	-2.28769400
C	2.50297700	4.80774600	0.91636300
H	0.82453600	5.67228200	1.93383800
C	3.03836600	4.30282400	-0.28362800
H	2.63963000	4.09338000	-2.40194700
H	3.08647400	4.74286500	1.83664400
C	4.32618400	3.56753400	-0.29710700
C	4.53365500	2.50544800	-1.19377900
C	5.34821400	3.82941500	0.63580100
C	5.67855600	1.70962600	-1.12984900

H	3.76395100	2.26089300	-1.92832200
C	6.48260100	3.02503500	0.70682400
H	5.25044900	4.67249800	1.32275400
C	6.65785200	1.93132900	-0.15506800
H	5.79553000	0.88827400	-1.83742400
H	7.23798600	3.23853100	1.46662300
C	2.85469300	-3.76524000	-0.11150800
C	2.89291400	-2.42407600	-0.53895600
C	4.03404600	-4.32872200	0.40451200
C	4.05553100	-1.66820500	-0.43502600
H	1.98080400	-1.95621300	-0.91410000
C	5.21100200	-3.57893700	0.48833000
H	4.03596000	-5.37307600	0.72496500
C	5.22380400	-2.23885700	0.08293400
H	4.06917700	-0.61897800	-0.73339700
H	6.12905600	-4.03110300	0.86845900
C	6.41753800	-1.41644900	2.63159500
C	6.80668300	-0.87064400	1.39647400
C	6.91993300	-0.88907300	3.82129800
N	6.39491100	-1.42412200	0.16926600
C	7.65614800	0.25711000	1.37651200
C	7.82444500	0.17576700	3.80295700
H	6.61010300	-1.32990200	4.77190600
C	7.15114700	-1.18461800	-0.99222600
C	7.84806900	0.97646400	0.05388300
C	8.17913100	0.74264400	2.57698800
H	8.23574400	0.57173400	4.73372300
C	7.11132800	-2.06914800	-2.08430000
C	7.97165800	-0.03849600	-1.05504600
H	8.85774700	1.59953900	2.54733100
C	7.90200600	-1.82901500	-3.20759400
H	6.46719300	-2.94763200	-2.04400500
C	8.77117700	0.17051600	-2.18050900
C	8.74802600	-0.71691700	-3.25861300
H	7.86336900	-2.52760800	-4.04694500
H	9.39981100	1.06441700	-2.21891500
H	9.37259900	-0.53547200	-4.13569100
H	5.72980600	-2.26044900	2.66105800
H	8.76515300	1.58418400	0.10462700

Ad[10]CPP-OH

C	-8.53473200	0.08112500	1.36093900
C	-8.29995900	1.45214100	1.34930300
C	-8.43738700	2.20384900	0.16724600
C	-9.05046700	1.56227200	-0.92795700
C	-9.28642000	0.18967000	-0.91607600
C	-8.91816200	-0.60148300	0.19105700
H	-8.26075200	-0.48667200	2.25176100
H	-7.85173000	1.91156300	2.23193500
H	-9.26673200	2.13064300	-1.83548500
H	-9.68155200	-0.28877300	-1.81519400
C	-7.64972800	3.45277300	0.02418300
C	-6.96960300	3.68719600	-1.18604500
C	-7.28288400	4.24844800	1.12783400
C	-5.85260400	4.51547900	-1.23711500
H	-7.22733700	3.10230000	-2.07067600
C	-6.16304600	5.07487500	1.07754000
H	-7.83849900	4.16272500	2.06444400
C	-5.36222200	5.14709800	-0.07963200
H	-5.27291300	4.55822100	-2.16109600
H	-5.86504800	5.62292000	1.97425300
C	-8.57215800	-2.03880000	0.06588000
C	-8.00587900	-2.48853500	-1.14193400
C	-8.45550800	-2.89344600	1.18077500
C	-7.17586900	-3.60401200	-1.17646200
H	-8.08619800	-1.87097400	-2.03805100
C	-7.62359500	-4.01012800	1.14619100
H	-8.95621000	-2.63339500	2.11621600
C	-6.87344700	-4.32343700	-0.00523300
H	-6.63712600	-3.82341400	-2.09975700
H	-7.49292100	-4.60267400	2.05455300
C	-5.61352200	-5.10482600	0.04819000
C	-4.81803400	-5.04066600	1.20801000
C	-5.02295700	-5.66322100	-1.10296100
C	-3.45876100	-5.33852900	1.16916200
H	-5.23562500	-4.62199600	2.12525600
C	-3.66146300	-5.95253500	-1.14487600
H	-5.62473800	-5.80891100	-2.00268900
C	-2.83141900	-5.70946300	-0.03349800
H	-2.85197900	-5.15134300	2.05741200
H	-3.22174400	-6.31973200	-2.07508400
C	-3.95001100	5.59688900	-0.04212700

C	-3.26535900	6.05393300	-1.18462900
C	-3.17782800	5.32614600	1.10298200
C	-1.87332000	6.09657000	-1.22256000
H	-3.83118200	6.33846500	-2.07467200
C	-1.78784400	5.36753700	1.06427200
H	-3.66793800	4.96361300	2.00834300
C	-1.09851600	5.68359000	-0.12091600
H	-1.37664400	6.42033900	-2.13974700
H	-1.22885700	5.04120700	1.94292200
C	-1.35968000	-5.57658700	-0.15844100
C	-0.47598200	-5.83755900	0.90471900
C	-0.83058900	-4.95466300	-1.30525800
C	0.84593100	-5.39266900	0.86875200
H	-0.84261900	-6.35288100	1.79537200
C	0.48429500	-4.50245200	-1.33746500
H	-1.48660900	-4.74333700	-2.15178800
C	1.33351800	-4.66127200	-0.22862400
H	1.48881800	-5.54874200	1.73836300
H	0.84899400	-3.97484800	-2.22147700
C	0.35161000	5.39506100	-0.23035300
C	1.20439300	5.41780000	0.88895600
C	0.88870800	4.90721700	-1.43676700
C	2.48743900	4.87903200	0.83236400
H	0.84345700	5.82772100	1.83444900
C	2.17374700	4.37531100	-1.49498000
H	0.26434600	4.87849300	-2.33178900
C	2.98519900	4.29677000	-0.34840200
H	3.09095700	4.85135400	1.74179400
H	2.54172100	3.97926400	-2.44353100
C	4.24983900	3.52265400	-0.34470700
C	5.32253700	3.83564600	0.51277700
C	4.37790600	2.37427700	-1.14381100
C	6.43313000	3.00166500	0.61397300
H	5.28352300	4.74617700	1.11460900
C	5.49497500	1.54362700	-1.04885900
H	3.56851100	2.09459200	-1.82091200
C	6.52171100	1.82222700	-0.14121900
H	7.24347900	3.25948100	1.29625000
H	5.55311600	0.65773800	-1.68074500
C	2.63093600	-3.94404200	-0.17173500
C	2.67507100	-2.59960000	-0.58790900

C	3.80644300	-4.51559600	0.34445500
C	3.83784300	-1.84656500	-0.46731200
H	1.76622000	-2.12606600	-0.96363600
C	4.98350300	-3.76792400	0.44574700
H	3.80370200	-5.56239400	0.65673100
C	5.00011300	-2.42354100	0.05618300
H	3.85262900	-0.79358500	-0.75145200
H	5.89695500	-4.22261400	0.83407600
C	6.02387500	-1.54447800	2.63334100
C	6.49798000	-1.01994600	1.41712200
C	6.42698400	-0.98772300	3.84542300
N	6.16680900	-1.60560700	0.18354200
C	7.34021600	0.11222400	1.43861900
C	7.31313600	0.09307400	3.87189300
H	6.05107900	-1.41386100	4.77872700
C	6.96029900	-1.35131600	-0.94581500
C	7.66643900	0.82829000	0.12744300
C	7.75394900	0.63850200	2.66636000
H	7.64114600	0.51960800	4.82207100
C	6.95327400	-2.22681000	-2.04734200
C	7.78023000	-0.20414200	-0.98145700
H	8.41464000	1.50675100	2.66014500
C	7.76833800	-1.97454300	-3.14912300
H	6.31351800	-3.10911100	-2.02856800
C	8.59835300	0.02501900	-2.09074000
C	8.60548000	-0.85432900	-3.17403300
H	7.75328700	-2.66775100	-3.99369800
H	9.21188600	0.92739800	-2.09596800
H	9.24439900	-0.65914700	-4.03778000
H	5.34733400	-2.39781300	2.62751100
O	8.85242900	1.60096800	0.24477800
H	9.56347500	0.99207100	0.49167900

Ad[10]CPP$^+$

C	-7.44959900	-0.10875000	1.21464900
C	-7.30582200	1.27491900	1.20602400
C	-7.49222500	2.02015200	0.02685200
C	-8.00999400	1.33468700	-1.08990100
C	-8.15556300	-0.05050300	-1.08057800
C	-7.78734200	-0.81445200	0.04480000
H	-7.16862200	-0.65850300	2.11451900

H	-6.91716600	1.76633000	2.09962000
H	-8.24943000	1.88786600	-2.00075600
H	-8.50601600	-0.55358300	-1.98441500
C	-6.89092600	3.37200100	-0.07231300
C	-6.23895900	3.75081400	-1.26088200
C	-6.70672600	4.20077100	1.05284800
C	-5.30907900	4.78607100	-1.27474500
H	-6.36388300	3.14581200	-2.16052300
C	-5.77280200	5.23294200	1.04116600
H	-7.26097900	3.99563200	1.97112500
C	-4.98642100	5.48985800	-0.09944800
H	-4.73332900	4.96209100	-2.18531200
H	-5.61298800	5.81595900	1.95091800
C	-7.47275300	-2.26155800	-0.03945700
C	-6.91989600	-2.77565500	-1.22714000
C	-7.44162500	-3.09621800	1.09577100
C	-6.20459500	-3.96926400	-1.23135000
H	-6.93449300	-2.17072300	-2.13534200
C	-6.72283600	-4.28896400	1.09296100
H	-7.93579000	-2.77609000	2.01556300
C	-6.00764900	-4.70415600	-0.04751300
H	-5.68033700	-4.26298400	-2.14257900
H	-6.66662400	-4.87844100	2.01089300
C	-4.87047300	-5.65302400	0.02536200
C	-4.05654100	-5.65405100	1.17354800
C	-4.39408700	-6.35723900	-1.09862600
C	-2.75892500	-6.15394700	1.13605200
H	-4.39314600	-5.13496100	2.07251700
C	-3.09512900	-6.85766400	-1.13643400
H	-5.02918600	-6.45828700	-1.98130600
C	-2.21470400	-6.68058500	-0.05040700
H	-2.11975500	-6.00922400	2.00867100
H	-2.74120200	-7.34255200	-2.04890900
C	-3.70321800	6.22653300	-0.02858300
C	-3.10445300	6.82815200	-1.15428500
C	-2.91598900	6.11907400	1.13377900
C	-1.74633800	7.13231900	-1.17146200
H	-3.70165700	7.01020000	-2.05016100
C	-1.55967900	6.42146100	1.11632900
H	-3.34269600	5.67252400	2.03325600
C	-0.92035300	6.85260500	-0.06287700

H	-1.31266300	7.55043900	-2.08211900
H	-0.96949000	6.20063600	2.00660900
C	-0.74057800	-6.73290100	-0.20333800
C	0.13053200	-6.86242100	0.89640500
C	-0.16403900	-6.37597500	-1.43920000
C	1.46056400	-6.45246900	0.81446200
H	-0.25786600	-7.20950000	1.85573500
C	1.16136400	-5.96748400	-1.52313400
H	-0.79274800	-6.30675200	-2.32813300
C	1.97478000	-5.90766200	-0.37645200
H	2.07458000	-6.45757200	1.71836900
H	1.55006100	-5.61771900	-2.48219300
C	0.55065900	6.73582200	-0.17497100
C	1.38669600	6.68318200	0.95999700
C	1.14685500	6.42704600	-1.41672300
C	2.66634300	6.14396400	0.88902200
H	1.00202000	6.99613600	1.93173800
C	2.42702100	5.89539800	-1.49026900
H	0.56067200	6.50554700	-2.33314800
C	3.18244100	5.64584300	-0.32554300
H	3.23098500	6.01871700	1.81479900
H	2.81815300	5.60738700	-2.46770300
C	4.29150400	4.67424400	-0.31941200
C	5.22589100	4.57388500	0.74007100
C	4.32989200	3.65653600	-1.29547500
C	5.98276000	3.42472800	0.93155300
H	5.32476200	5.39463600	1.45238100
C	5.10395200	2.51953400	-1.12479200
H	3.65468700	3.69885800	-2.14976200
C	5.87118900	2.32772500	0.04875000
H	6.66687100	3.37348600	1.78026900
H	5.01580400	1.70798400	-1.84850200
C	3.17605800	-5.04099900	-0.35045000
C	3.09075200	-3.80214500	-1.01932200
C	4.32139100	-5.28552800	0.43142600
C	4.04012300	-2.81162000	-0.82238300
H	2.21402900	-3.58171300	-1.62901900
C	5.29605500	-4.29747900	0.62137000
H	4.44228600	-6.25332400	0.92219000
C	5.12086100	-3.04324400	0.03480600
H	3.91605900	-1.82978800	-1.28370500

H	6.16349800	-4.49253200	1.25514100
C	4.99496400	-1.80965500	2.60015200
C	5.58925600	-1.15496300	1.49591000
C	4.61554800	-1.09513100	3.71766800
N	5.92004300	-1.87186900	0.35677800
C	5.75395100	0.27265500	1.52135900
C	4.76716500	0.30784500	3.76081000
H	4.18101700	-1.62369400	4.56867000
C	6.65531900	-1.28945500	-0.65199000
C	6.22743900	0.93123000	0.34138100
C	5.29833600	0.97054000	2.67897400
H	4.44469100	0.86703100	4.64064600
C	7.17039700	-2.07604000	-1.70724900
C	6.83754400	0.13032700	-0.66624300
H	5.37097500	2.05307000	2.70434200
C	7.89491100	-1.47820600	-2.71949000
H	7.00533000	-3.15291500	-1.69888000
C	7.59002300	0.70568800	-1.73252500
C	8.11714100	-0.07988300	-2.73187100
H	8.31421200	-2.09845500	-3.51459500
H	7.75919500	1.78271300	-1.72341300
H	8.71194000	0.36928100	-3.52889800
H	4.84579500	-2.88547300	2.56940000

Ad[10]CPP·

C	9.08675400	-0.95717400	-0.96938600
C	9.15256400	0.43397200	-0.96560200
C	8.70221600	1.17956300	0.14264000
C	8.41770600	0.46172700	1.31952500
C	8.35211800	-0.92793800	1.31579100
C	8.56774000	-1.66290400	0.13491100
H	9.36042500	-1.49967700	-1.87726700
H	9.47648300	0.95287100	-1.87074600
H	8.08602300	0.99683600	2.21103300
H	7.97151300	-1.43456200	2.20439400
C	8.18876800	2.56566500	0.01618800
C	7.99884200	3.41168000	1.12716000
C	7.55625500	2.93937400	-1.18468400
C	7.05843800	4.43880900	1.09598200
H	8.53767200	3.21283300	2.05641600
C	6.61785100	3.96553400	-1.21579900

H	7.68494000	2.32290900	-2.07593300
C	6.26736100	4.66773100	-0.04772400
H	6.88324400	5.02582000	2.00038700
H	6.04559900	4.12132100	-2.13189400
C	7.92930200	-2.99538200	0.00211900
C	7.67077400	-3.82941100	1.10806100
C	7.25960200	-3.30149800	-1.19773700
C	6.64629300	-4.77258300	1.07316500
H	8.22881400	-3.68505900	2.03608700
C	6.23671600	-4.24351100	-1.23228000
H	7.43687500	-2.69050400	-2.08447300
C	5.83462500	-4.92526100	-0.06882400
H	6.42603700	-5.35099400	1.97321200
H	5.64898000	-4.34290800	-2.14646400
C	4.47269900	-5.50912400	-0.00829700
C	3.79789800	-5.97402000	-1.15404600
C	3.71089700	-5.35532400	1.16545800
C	2.41241100	-6.11563200	-1.16713800
H	4.36137400	-6.17699600	-2.06746300
C	2.32696900	-5.50174600	1.15378200
H	4.19239700	-4.99538600	2.07639800
C	1.63639100	-5.80560200	-0.03343000
H	1.91675000	-6.42955300	-2.08850500
H	1.76327100	-5.25924100	2.05671200
C	4.95237700	5.35075400	0.01344400
C	4.17705000	5.24360400	1.18366200
C	4.31503600	5.86618300	-1.13249500
C	2.80485500	5.47504900	1.16542300
H	4.62993100	4.85043200	2.09547200
C	2.94097500	6.09099600	-1.15302300
H	4.89511000	6.03833600	-2.04176400
C	2.14097300	5.82052500	-0.02570100
H	2.22254700	5.26326700	2.06429100
H	2.47067300	6.43720600	-2.07613800
C	0.17288700	-5.58613700	-0.11033400
C	-0.38172000	-4.98615200	-1.25625700
C	-0.67867400	-5.77257200	0.99443800
C	-1.68632600	-4.50176000	-1.26066000
H	0.25052600	-4.80958600	-2.12865200
C	-1.98650800	-5.29320000	0.98720800
H	-0.30284800	-6.28362700	1.88377300

C	-2.50665700	-4.59608900	-0.12101300
H	-2.04391600	-3.96095100	-2.13882200
H	-2.61269400	-5.44542400	1.86881600
C	0.67044100	5.66785300	-0.11666100
C	0.10348200	5.09209300	-1.26940300
C	-0.18463100	5.88538100	0.98051600
C	-1.21591300	4.65322100	-1.28562900
H	0.73594500	4.89512500	-2.13705100
C	-1.50659700	5.44946700	0.96295800
H	0.20107100	6.38233000	1.87349300
C	-2.04210000	4.76868200	-0.15024400
H	-1.58061500	4.12802200	-2.16960700
H	-2.13159400	5.62293600	1.84105900
C	-3.34889400	4.08256700	-0.09699500
C	-4.06514300	3.73725200	-1.26349700
C	-3.86924900	3.60424500	1.12528700
C	-5.19011100	2.92979500	-1.22189500
H	-3.71806100	4.09464800	-2.23468200
C	-4.99666000	2.80153600	1.17789000
H	-3.33242000	3.80596900	2.05381800
C	-5.70428600	2.41250000	0.00296900
H	-5.65723700	2.65245900	-2.16394300
H	-5.29738800	2.40060300	2.14372200
C	-3.79947700	-3.87694600	-0.06019500
C	-4.25604100	-3.31430500	1.14470300
C	-4.56522300	-3.60393000	-1.20703100
C	-5.37902800	-2.49535300	1.20025000
H	-3.68196700	-3.46469300	2.06110800
C	-5.69216100	-2.78755600	-1.16766800
H	-4.27243000	-4.03437200	-2.16700300
C	-6.11615300	-2.19277100	0.03773000
H	-5.64846600	-2.04289000	2.15229200
H	-6.22339500	-2.58906900	-2.09541700
C	-8.26088600	-1.22516600	-2.14176400
C	-7.61026500	-0.57556700	-1.08961100
C	-8.66675500	-0.50696600	-3.26811600
N	-7.19697000	-1.27619500	0.07858900
C	-7.37663800	0.82538200	-1.13561600
C	-8.45559000	0.87457400	-3.32525400
H	-9.17485400	-1.02169400	-4.08648500
C	-7.48617400	-0.56816800	1.27719400

C	-6.76431700	1.43828300	0.04910500
C	-7.83204600	1.53271500	-2.26690500
H	-8.80323400	1.44681500	-4.18831100
C	-8.00242300	-1.21963400	2.40061800
C	-7.24829100	0.82948000	1.29260400
H	-7.73173600	2.61800900	-2.29305900
C	-8.28208600	-0.49732400	3.56107600
H	-8.18225400	-2.29492200	2.35211300
C	-7.57115800	1.54242100	2.46605500
C	-8.07209800	0.88699900	3.58835100
H	-8.68736400	-1.01016000	4.43608500
H	-7.45580100	2.62696200	2.47804600
H	-8.32024500	1.46053200	4.48426800
H	-8.46282200	-2.29404600	-2.06151800

Ad[10]CPP-H (optimized in dichloromethane)

C	-8.36212500	-0.06151300	1.32745200
C	-8.16578300	1.31295700	1.31783700
C	-8.31022200	2.05705400	0.13905700
C	-8.88613500	1.40093400	-0.96015800
C	-9.08362100	0.02468700	-0.95028400
C	-8.71156300	-0.75162800	0.15868900
H	-8.08950400	-0.61744700	2.22575500
H	-7.74793200	1.78296500	2.20938700
H	-9.10038000	1.95741000	-1.87509500
H	-9.44655000	-0.46157100	-1.85830800
C	-7.55911000	3.33505800	0.00827700
C	-6.87916200	3.59577900	-1.18961900
C	-7.23145500	4.13421500	1.11442000
C	-5.79253000	4.46017600	-1.22692500
H	-7.11023400	3.01258500	-2.08237200
C	-6.14144800	4.99619300	1.07827400
H	-7.78529200	4.02341400	2.04902200
C	-5.33700900	5.09983900	-0.06685600
H	-5.21160100	4.52921800	-2.14825500
H	-5.86878000	5.54169300	1.98420400
C	-8.33196000	-2.18612300	0.04241100
C	-7.75088400	-2.62700500	-1.15449000
C	-8.20777400	-3.02966400	1.15755500
C	-6.90351700	-3.72650600	-1.17936700
H	-7.83831700	-2.01962600	-2.05654000

C	-7.35826000	-4.13016700	1.13282600
H	-8.71144500	-2.77432200	2.09229400
C	-6.59984600	-4.43403800	-0.00847100
H	-6.36062000	-3.94157700	-2.10096300
H	-7.22032100	-4.70983800	2.04796800
C	-5.32575700	-5.20022000	0.05406100
C	-4.53819900	-5.11379400	1.21058900
C	-4.72763500	-5.75862700	-1.08591900
C	-3.17751100	-5.39270000	1.17820800
H	-4.96255600	-4.69805900	2.12583000
C	-3.36510100	-6.03123600	-1.12065700
H	-5.31996600	-5.91470200	-1.98981200
C	-2.54473600	-5.76634400	-0.01405700
H	-2.57970000	-5.19272000	2.06936500
H	-2.92113500	-6.39554100	-2.04955800
C	-3.93580800	5.59469800	-0.01450500
C	-3.25975000	6.08577700	-1.14082300
C	-3.16817300	5.33145500	1.12815500
C	-1.87174300	6.16881900	-1.16713900
H	-3.82285900	6.35999400	-2.03541700
C	-1.78247500	5.41184300	1.10052900
H	-3.65176300	4.94824800	2.02814400
C	-1.09692300	5.76242100	-0.07022600
H	-1.38208400	6.51327600	-2.08014200
H	-1.22513000	5.09309100	1.98251900
C	-1.07056500	-5.61450200	-0.13624700
C	-0.19085500	-5.83979800	0.93116000
C	-0.54899500	-5.00830400	-1.28797500
C	1.12002400	-5.37189900	0.89315200
H	-0.54931000	-6.34179300	1.83233600
C	0.75483400	-4.53403600	-1.32286600
H	-1.20020500	-4.82580900	-2.14452400
C	1.59709900	-4.65355900	-0.21068800
H	1.75927800	-5.50340400	1.76911400
H	1.10943900	-4.01460100	-2.21539900
C	0.36476300	5.50878700	-0.17340200
C	0.92380500	5.07575900	-1.38414800
C	1.19997700	5.50735700	0.95238800
C	2.21518800	4.56695500	-1.44365600
H	0.31641600	5.06978800	-2.29076600
C	2.48839100	4.98931400	0.89477700

H	0.82409400	5.87453600	1.90906600
C	3.00799800	4.45651800	-0.29342700
H	2.59550100	4.20894400	-2.40195100
H	3.07621300	4.94175500	1.81320300
C	4.28175100	3.68929700	-0.29724400
C	4.44268000	2.59416500	-1.15375600
C	5.32003200	3.95127300	0.60907900
C	5.55772700	1.76408500	-1.07224900
H	3.65968000	2.34838400	-1.87306500
C	6.42757200	3.11598000	0.69404300
H	5.25961500	4.81888800	1.26910500
C	6.55411100	1.98465600	-0.12121300
H	5.63354000	0.91524400	-1.75271100
H	7.20323800	3.34185400	1.42934000
C	2.87882600	-3.90178900	-0.16050600
C	2.88328100	-2.56574800	-0.58666200
C	4.06768400	-4.43171900	0.35432700
C	4.02046600	-1.78018600	-0.47437300
H	1.96425800	-2.12061800	-0.97146500
C	5.22009700	-3.65110500	0.44579200
H	4.10019100	-5.47390900	0.67910500
C	5.19902400	-2.31223700	0.05026800
H	3.99860400	-0.73204200	-0.77567800
H	6.14384000	-4.08432400	0.83365600
C	6.34599300	-1.48356000	2.61959200
C	6.71399800	-0.89963400	1.39939400
C	6.80875600	-0.95679200	3.82042900
N	6.33882500	-1.45508200	0.16211900
C	7.50743800	0.26294800	1.40792700
C	7.65636200	0.14813000	3.82964700
H	6.51384800	-1.43158400	4.75884100
C	7.10891800	-1.17056300	-0.97926700
C	7.71850400	1.00620800	0.10205400
C	7.99369700	0.74904900	2.62062600
H	8.04009200	0.54744000	4.77023100
C	7.14285100	-2.04991300	-2.07095500
C	7.87553200	0.00758000	-1.01802000
H	8.63510200	1.63398700	2.61471900
C	7.94816900	-1.77307900	-3.16957100
H	6.54372100	-2.96020200	-2.05045900
C	8.69405800	0.25326100	-2.11927000

C	8.74005100	-0.62645200	-3.19646800
H	7.96499900	-2.47176800	-4.00897800
H	9.28977900	1.16971800	-2.13654700
H	9.38058300	-0.41457800	-4.05453200
H	5.70539600	-2.36386200	2.63119100
H	8.63829900	1.60433000	0.18407100

Ad[10]CPP-OH (optimized in dichloromethane)

C	-8.45638900	0.06867500	1.34895400
C	-8.23198800	1.43889000	1.33690300
C	-8.37351400	2.18573200	0.15954000
C	-8.97187900	1.54100500	-0.93437400
C	-9.19742700	0.16912000	-0.92206900
C	-8.83227600	-0.61423800	0.18418000
H	-8.18706000	-0.49271300	2.24488400
H	-7.79640800	1.90076600	2.22423800
H	-9.18336700	2.10167900	-1.84737800
H	-9.57897800	-0.30989100	-1.82630200
C	-7.60257400	3.45110000	0.02206000
C	-6.92770700	3.69973900	-1.18120900
C	-7.25565500	4.24767900	1.12412700
C	-5.83038700	4.54988900	-1.22857300
H	-7.17324100	3.11780000	-2.07091100
C	-6.15538700	5.09612200	1.07759900
H	-7.80385000	4.14608500	2.06310400
C	-5.35853800	5.18758400	-0.07388300
H	-5.25581000	4.60906400	-2.15450000
H	-5.86970300	5.64059300	1.98010100
C	-8.48945100	-2.05777200	0.06631600
C	-7.92884000	-2.51431400	-1.13443300
C	-8.38081400	-2.90424800	1.18079000
C	-7.11333800	-3.63750400	-1.16523800
H	-8.00548600	-1.90450300	-2.03583500
C	-7.56339300	-4.02864200	1.15005000
H	-8.87063900	-2.63484200	2.11893200
C	-6.82246700	-4.35433600	0.00326900
H	-6.58329100	-3.86770200	-2.09064200
H	-7.43587900	-4.61214900	2.06425500
C	-5.57130400	-5.15819700	0.05656100
C	-4.77451900	-5.09776900	1.20836900

C	-4.99675500	-5.73162800	-1.08813900
C	-3.42261800	-5.41534400	1.16669700
H	-5.18092600	-4.67243200	2.12731200
C	-3.64279400	-6.04295100	-1.13221300
H	-5.59892200	-5.86886700	-1.98857400
C	-2.80811100	-5.80339100	-0.03054700
H	-2.81386500	-5.23439900	2.05449500
H	-3.21542700	-6.41751000	-2.06483000
C	-3.95192300	5.66848900	-0.03463400
C	-3.27816100	6.13767600	-1.17175300
C	-3.17958100	5.41282800	1.10661300
C	-1.88958000	6.20310700	-1.20925200
H	-3.84424900	6.40698300	-2.06590900
C	-1.79310800	5.47717100	1.06855100
H	-3.66099000	5.04743400	2.01513000
C	-1.11233100	5.80198500	-0.11226200
H	-1.40174500	6.52811200	-2.13040600
H	-1.23233800	5.16441200	1.95066800
C	-1.33136800	-5.68804900	-0.16211600
C	-0.45101300	-5.93339800	0.90034100
C	-0.80277400	-5.09243400	-1.31621500
C	0.86839900	-5.49057300	0.85721800
H	-0.81510900	-6.42914300	1.80269200
C	0.50973900	-4.64320100	-1.35630100
H	-1.45411100	-4.89585600	-2.16952100
C	1.35357100	-4.77857900	-0.24716600
H	1.50852600	-5.63478500	1.73054800
H	0.87006300	-4.12881300	-2.24947500
C	0.34379700	5.52209500	-0.22401000
C	1.19466200	5.54595600	0.88964500
C	0.87597300	5.03496900	-1.42628000
C	2.47247100	5.00128500	0.83282000
H	0.83847000	5.95447800	1.83718800
C	2.15526900	4.49683200	-1.48469100
H	0.25434500	5.00843000	-2.32286300
C	2.96169800	4.41308100	-0.34189000
H	3.07459000	4.97559500	1.74301300
H	2.51463200	4.09373300	-2.43321100
C	4.21464600	3.61275800	-0.33436200
C	5.29179700	3.90397200	0.51626700
C	4.31214000	2.45939900	-1.12033300

C	6.37897700	3.04467000	0.62119600
H	5.27737100	4.81586600	1.11675600
C	5.40445400	1.60213500	-1.01927100
H	3.49821000	2.19288800	-1.79656400
C	6.43771900	1.85822900	-0.11945000
H	7.19472900	3.29171200	1.30111400
H	5.43219200	0.70976700	-1.64443400
C	2.64405100	-4.04181700	-0.19620300
C	2.65929400	-2.70300100	-0.61299500
C	3.82828200	-4.58501700	0.31577000
C	3.80171100	-1.92647000	-0.49189200
H	1.74426300	-2.24795700	-0.99553500
C	4.98580400	-3.81315300	0.41674700
H	3.85205600	-5.62947000	0.63386900
C	4.97415700	-2.47126300	0.03230400
H	3.78613200	-0.87551300	-0.78312500
H	5.90490600	-4.25394400	0.80726400
C	6.00871900	-1.60460600	2.61469600
C	6.44559700	-1.04473700	1.40450400
C	6.40805800	-1.06170600	3.82927200
N	6.11793500	-1.62060100	0.16632600
C	7.25071500	0.10846400	1.43889100
C	7.25795700	0.04167800	3.86617000
H	6.05952100	-1.51964700	4.75758000
C	6.91134000	-1.33526300	-0.95473500
C	7.57034100	0.84932000	0.14167700
C	7.66512800	0.61937500	2.66937100
H	7.58767300	0.45853900	4.81952700
C	6.94792700	-2.20620500	-2.05438700
C	7.69478600	-0.16828500	-0.97953200
H	8.30753700	1.50096800	2.67469300
C	7.76617400	-1.93122800	-3.14254200
H	6.33934100	-3.11019100	-2.04566200
C	8.52163600	0.08138800	-2.07428900
C	8.56830600	-0.79101700	-3.15571700
H	7.78346000	-2.62511500	-3.98589000
H	9.12102400	0.99289600	-2.06789000
H	9.21594100	-0.57798900	-4.00813300
H	5.36396900	-2.48173500	2.60473600
O	8.75141700	1.61551400	0.27279000
H	9.47156400	1.01509600	0.51179600

Ad[10]CPP$^+$ (optimized in dichloromethane)

C	-7.41565900	-0.26486900	1.18749500
C	-7.31129600	1.11981600	1.18066800
C	-7.51091900	1.85817900	0.00637900
C	-7.99728400	1.16151000	-1.11030300
C	-8.10254800	-0.22511100	-1.10320600
C	-7.72497400	-0.97632800	0.02034300
H	-7.12850800	-0.80369200	2.09174100
H	-6.94620900	1.61878900	2.07968100
H	-8.23689000	1.70380300	-2.02732100
H	-8.42202400	-0.73403200	-2.01497000
C	-6.94983900	3.23309200	-0.08543600
C	-6.29970700	3.63016700	-1.26181000
C	-6.80061700	4.06094400	1.03786400
C	-5.40229300	4.69085100	-1.26554900
H	-6.39750200	3.02666000	-2.16565700
C	-5.90070600	5.12040300	1.03523800
H	-7.34629200	3.83459900	1.95617700
C	-5.11395900	5.40026200	-0.09216000
H	-4.82558500	4.88531700	-2.17152200
H	-5.76102700	5.69823200	1.95127700
C	-7.37302300	-2.41979000	-0.06030600
C	-6.80084100	-2.91889800	-1.23822500
C	-7.33048500	-3.24924900	1.07106100
C	-6.06132600	-4.09477600	-1.23694600
H	-6.82261300	-2.31843600	-2.14905200
C	-6.58818000	-4.42473300	1.07335300
H	-7.82826100	-2.93851400	1.99190800
C	-5.85910600	-4.82346700	-0.05715800
H	-5.52794000	-4.37684700	-2.14626300
H	-6.52028500	-5.00500400	1.99600200
C	-4.70235300	-5.75528200	0.02347100
C	-3.89465800	-5.73107700	1.16830500
C	-4.21150700	-6.45479100	-1.08938500
C	-2.59108300	-6.20852600	1.13906600
H	-4.24044200	-5.21364800	2.06461100
C	-2.90723100	-6.93617400	-1.11716900
H	-4.83433000	-6.56527100	-1.97958000
C	-2.03626600	-6.73590600	-0.03498800

H	-1.96058900	-6.04752800	2.01463300
H	-2.54278400	-7.41412600	-2.02869000
C	-3.84804100	6.17614800	-0.00814400
C	-3.26975100	6.81587600	-1.11453500
C	-3.05787300	6.05327000	1.14240600
C	-1.92104300	7.15553100	-1.12501100
H	-3.86708300	6.99626100	-2.01081800
C	-1.71052100	6.38626800	1.12902600
H	-3.46817300	5.57368600	2.03258400
C	-1.08858500	6.86424200	-0.03294400
H	-1.50002900	7.59781900	-2.02985800
H	-1.11401500	6.15417000	2.01184200
C	-0.55504800	-6.77277400	-0.18018200
C	0.30530400	-6.86228300	0.92378300
C	0.02050600	-6.42986500	-1.41297700
C	1.62553500	-6.42928400	0.84304100
H	-0.08131500	-7.18952300	1.89067400
C	1.33798700	-6.00223600	-1.49613100
H	-0.59978700	-6.38463000	-2.30928600
C	2.13619500	-5.90021900	-0.34937700
H	2.23110400	-6.40365800	1.75177400
H	1.72278100	-5.65779000	-2.45817000
C	0.39324100	6.78115300	-0.15114100
C	1.22564100	6.72062300	0.97614900
C	0.98371000	6.49354400	-1.39139000
C	2.51005600	6.19522500	0.89502900
H	0.83993000	7.00467400	1.95643700
C	2.27121000	5.98190300	-1.47548600
H	0.39473900	6.56816800	-2.30651200
C	3.02467600	5.72514800	-0.32139100
H	3.07666600	6.05597100	1.81763700
H	2.65954400	5.70069800	-2.45590600
C	4.14767800	4.75144000	-0.33182000
C	5.14038800	4.70036000	0.66123200
C	4.10303200	3.69264900	-1.24844700
C	5.92052300	3.56327400	0.84382700
H	5.27033300	5.54103900	1.34503300
C	4.89092100	2.56526500	-1.08433900
H	3.36518900	3.69524700	-2.05051900
C	5.74990700	2.44188200	0.01892800
H	6.64615300	3.54189000	1.65856800

H	4.75488900	1.72318500	-1.76429100
C	3.32113800	-5.00137400	-0.33222200
C	3.18948800	-3.77383800	-0.99958300
C	4.47017900	-5.20705700	0.44248100
C	4.10269800	-2.75375200	-0.80840500
H	2.30696800	-3.58107600	-1.60977700
C	5.40995100	-4.18903600	0.62674000
H	4.62440300	-6.16493800	0.94284500
C	5.18867600	-2.94637200	0.04320400
H	3.94092900	-1.77986800	-1.27398600
H	6.28154900	-4.35496800	1.26204100
C	4.99760600	-1.68968500	2.58865300
C	5.57669900	-1.03174900	1.47901100
C	4.58938700	-0.97320400	3.68497100
N	5.94447300	-1.74426500	0.35767700
C	5.69169900	0.39294600	1.48857300
C	4.69377200	0.43407600	3.71221900
H	4.16909900	-1.50313500	4.54163100
C	6.67028200	-1.15006300	-0.64207500
C	6.16008900	1.04828400	0.31795500
C	5.20833000	1.09541100	2.63224200
H	4.34972000	0.99125200	4.58429600
C	7.22542000	-1.92680000	-1.68364500
C	6.80232800	0.26949100	-0.67199800
H	5.25755900	2.18005100	2.64574100
C	7.92970100	-1.31457400	-2.69022500
H	7.10882900	-3.00932500	-1.66502200
C	7.52797300	0.86331000	-1.74663500
C	8.09109100	0.09106700	-2.72449900
H	8.38001000	-1.92665000	-3.47389500
H	7.64576400	1.94685500	-1.75696000
H	8.66590400	0.55190200	-3.52859600
H	4.89052000	-2.77056100	2.58096900

Ad[10]CPP· (optimized in dichloromethane)

0 2

C	9.07534200	-0.94472400	-0.96413600
C	9.13864200	0.44403900	-0.96008300
C	8.69069000	1.18504000	0.14489400
C	8.40945700	0.46983400	1.31688700

C	8.34651000	-0.91739400	1.31295100
C	8.56198900	-1.64826900	0.13677500
H	9.34282800	-1.48195700	-1.87654800
H	9.45405500	0.95968300	-1.86961900
H	8.08347200	1.00106100	2.21241600
H	7.97382900	-1.42234100	2.20549400
C	8.17501200	2.57552500	0.02075300
C	7.98158500	3.41441100	1.12922500
C	7.54930900	2.94997500	-1.17636700
C	7.04247800	4.43928800	1.09739700
H	8.50959600	3.21243800	2.06360500
C	6.61203000	3.97371300	-1.20809200
H	7.68428400	2.34229500	-2.07235600
C	6.25878800	4.66918800	-0.04409700
H	6.86157500	5.01645200	2.00659000
H	6.04916600	4.13339600	-2.12897800
C	7.92516500	-2.98679800	0.00581000
C	7.66746800	-3.81510900	1.10894200
C	7.26278800	-3.29603400	-1.19023700
C	6.64743400	-4.75931800	1.07364000
H	8.21507600	-3.66520500	2.04186800
C	6.24447700	-4.23919800	-1.22531300
H	7.44290400	-2.69306300	-2.08153400
C	5.84325900	-4.91609100	-0.06597800
H	6.42493800	-5.32840700	1.97866700
H	5.66582200	-4.34467800	-2.14424500
C	4.47808400	-5.50415100	-0.00834200
C	3.80858100	-5.96654600	-1.15124500
C	3.71835300	-5.35355600	1.16008100
C	2.42587800	-6.10961500	-1.16611200
H	4.36878300	-6.16154600	-2.06794900
C	2.33729200	-5.50156800	1.14673500
H	4.19603100	-5.00074500	2.07542300
C	1.65193400	-5.80339800	-0.03706100
H	1.93497000	-6.41581800	-2.09224400
H	1.77570400	-5.26666300	2.05267100
C	4.93857600	5.35243200	0.01391600
C	4.16520700	5.24383200	1.17810400
C	4.30547100	5.86387400	-1.12904700
C	2.79519600	5.47246900	1.15768900
H	4.61544300	4.85784700	2.09395900

C	2.93357100	6.08613100	-1.15170900
H	4.88187300	6.03086700	-2.04120000
C	2.13624700	5.81572600	-0.02974100
H	2.21516500	5.26521700	2.05879400
H	2.46675700	6.42389600	-2.07936000
C	0.18434100	-5.58318800	-0.11464400
C	-0.36509100	-4.97822400	-1.25334500
C	-0.66555700	-5.77563900	0.98320200
C	-1.66695500	-4.49417600	-1.25730200
H	0.26406700	-4.80122800	-2.12755800
C	-1.97070400	-5.29634100	0.97639000
H	-0.29296700	-6.28551400	1.87418900
C	-2.48578700	-4.59494700	-0.12425100
H	-2.02202800	-3.95415700	-2.13664100
H	-2.59324700	-5.44937900	1.86009100
C	0.66159900	5.65991200	-0.12203400
C	0.10272800	5.07151900	-1.26493200
C	-0.19291400	5.88893100	0.96563600
C	-1.21394900	4.63203200	-1.28055600
H	0.73413900	4.86923700	-2.13181700
C	-1.51238700	5.45253700	0.94837700
H	0.18791800	6.38986300	1.85811300
C	-2.03983700	4.76165500	-0.15445700
H	-1.57458500	4.10161500	-2.16278500
H	-2.13588300	5.63094700	1.82622700
C	-3.35137100	4.07287300	-0.09962800
C	-4.06452300	3.72909200	-1.26146000
C	-3.86891700	3.60063500	1.11925000
C	-5.18687500	2.92139800	-1.21806300
H	-3.71984800	4.08321100	-2.23437600
C	-4.99411900	2.79807900	1.17311400
H	-3.33960800	3.81112100	2.04985300
C	-5.69570200	2.40405800	0.00290900
H	-5.65583700	2.64987500	-2.16085400
H	-5.29871200	2.40993600	2.14278600
C	-3.78215400	-3.87334600	-0.06267600
C	-4.24089600	-3.32010400	1.13905800
C	-4.54222800	-3.59542400	-1.20531100
C	-5.36143000	-2.50241200	1.19570100
H	-3.67736600	-3.48137800	2.05995200
C	-5.66620100	-2.77990700	-1.16438700

H	-4.24919900	-4.01775600	-2.16859700
C	-6.09238600	-2.18930400	0.03745900
H	-5.63631200	-2.06637700	2.15367100
H	-6.19619200	-2.58419800	-2.09335200
C	-8.26003900	-1.22904100	-2.12698100
C	-7.59780800	-0.58057800	-1.08630900
C	-8.68703600	-0.51235800	-3.24118900
N	-7.17298800	-1.27836100	0.07956200
C	-7.37550000	0.81552200	-1.13398500
C	-8.48462400	0.86527100	-3.29905900
H	-9.20407100	-1.02835500	-4.05249700
C	-7.47276700	-0.57277000	1.27728800
C	-6.75691700	1.42980700	0.05039500
C	-7.84868300	1.52184200	-2.25178500
H	-8.84627100	1.43743600	-4.15567300
C	-8.00533400	-1.22184300	2.38990400
C	-7.24592400	0.82007200	1.29499700
H	-7.75006500	2.60708200	-2.28295800
C	-8.30836900	-0.50061500	3.54002900
H	-8.18321700	-2.29745400	2.34407700
C	-7.58945200	1.53245800	2.45635600
C	-8.10560900	0.87947500	3.56873200
H	-8.72582400	-1.01387200	4.40845600
H	-7.47637900	2.61708500	2.47301200
H	-8.36936300	1.45351400	4.45916200
H	-8.45513200	-2.29939800	-2.05097600

11. References

[1] R. Liu, H. Gao, L. Zhou, Y. Ji, G. Zhang, Effects of N-Substitution on the Property of Acridone. *ChemistrySelect.* **2019**, *4*, 7797-7804.

[2] Q. Huang, G. Zhuang, H. Jia, M. Qian, S. Cui, S. Yang, P. Du, Photoconductive Curved-Nanographene/Fullerene Supramolecular Heterojunctions. *Angew. Chem. Int. Ed.* **2019**, *58*, 6244-6249.

[3] X. Zhang, K. Lan, C. Cheng, Figure-Eight Bismacrocycles Derived from a Tetraphenylmethane Core and Oligoparaphenylene Loops. *Org. Lett.* **2024**, *26*, 7853-7857.

[4] K. Kurihara, K. Yazaki, M. Akita, M. Yoshizawa, A Switchable Open/closed Polyaromatic Macrocycle that Shows Reversible Binding of Long Hydrophilic Molecules. *Angew. Chem. Int. Ed.* **2017**, *56*, 11360-11364.

[5] E. R. Darzi, E. S. Hirst, C. D. Weber, L. N. Zakharov, M. C. Lonergan, R. Jasti, Synthesis, Properties, and Design Principles of Donor-Acceptor Nanohoops. *ACS. Cent. Sci.* **2015**, *1*, 335-342.

[6] O. V. Dolomanov, L. J. Bourhis, R. J. Gildea, J. A. K. Howard, H. Puschmann, *J. Appl. Cryst.* **2009**, *42*, 339-341.

[7] G. M. Sheldrick, *Acta Cryst* **2015**, *A71*, 3-8.

[8] G. M. Sheldrick, *Acta Cryst* **2015**, *C71*, 3-8.

[9] Y. Segawa, H. Omachi, K. Itami, Theoretical Studies on the Structures and Strain Energies of Cycloparaphenylenes. *Org. Lett.* **2010**, *12*, 2262-2265.

[10] G. W. T. M. J. Frisch, H. B. Schlegel, G. E. Scuseria, M. A. Robb, J. R. Cheeseman, G. Scalmani, V. Barone, G. A. Petersson, H. Nakatsuji, X. Li, M. Caricato, A. V. Marenich, J. Bloino, B. G. Janesko, R. Gomperts, B. Mennucci, H. P. Hratchian, J. V. Ortiz, A. F. Izmaylov, J. L. Sonnenberg, D. Williams-Young, F. Ding, F. Lipparini, F. Egidi, J. Goings, B. Peng, A. Petrone, T. Henderson, D. Ranasinghe, V. G. Zakrzewski, J. Gao, N. Rega, G. Zheng, W. Liang, M. Hada, M. Ehara, K. Toyota, R. Fukuda, J. Hasegawa, M. Ishida, T. Nakajima, Y. Honda, O. Kitao, H. Nakai, T. Vreven, K. Throssell, J. A. Montgomery, Jr., J. E. Peralta, F. Ogliaro, M. J. Bearpark, J. J. Heyd, E. N. Brothers, K. N. Kudin, V. N. Staroverov, T. A. Keith, R. Kobayashi, J. Normand, K. Raghavachari, A. P. Rendell, J. C. Burant, S. S. Iyengar, J. Tomasi, M. Cossi, J. M. Millam, M. Klene, C. Adamo, R. Cammi, J. W. Ochterski, R. L. Martin, K. Morokuma, O. Farkas, J. B. Foresman, and D. J. Fox, Gaussian 16, Gaussian, Inc., Wallingford CT. *Revision A.03* **2016**.

[11] S. Grimme, J. Antony, S. Ehrlich, H. Krieg, A consistent and accurateab initioparametrization of density functional dispersion correction (DFT-D) for the 94 elements H-Pu. *J. Chem. Phys.* **2010**, *132*, 154104.

[12] S. Grimme, S. Ehrlich, L. Goerigk, Effect of the damping function in dispersion corrected density functional theory. *J. Comput. Chem.* **2011**, *32*, 1456-1465.

[13] F. Weigend, R. Ahlrichs, Balanced basis sets of split valence, triple zeta valence and quadruple zeta valence quality for H to Rn: Design and assessment of accuracy. *Phys. Chem. Chem. Phys.* **2005**, *7*, 3297-3305.

[14] F. Weigend, Accurate Coulomb-fitting basis sets for H to Rn. *Phys. Chem. Chem. Phys.* **2006**, *8*, 1057-1065.

[15] J. Tomasi, B. Mennucci, R. Cammi, Quantum mechanical continuum solvation models. *Chem. Rev.* **2005**, *105*, 2999-3094.

[16] W. Humphrey, A. Dalke, K. Schulten, VMD: visual molecular dynamics. *J. Mol. Graph.* **1996**, *14*, 33-38.

[17] T. Lu, F. Chen, Multiwfn: A multifunctional wavefunction analyzer. *J. Comput. Chem.* **2012**, *33*, 580-592.

[18] T. Yanai, D. P. Tew, N. C. Handy, A new hybrid exchange–correlation functional using the Coulomb-attenuating method (CAM-B3LYP). *Chem. Phys. Lett.* **2004**, *393*, 51-57.

[19] A. Charaf-Eddin, A. Planchat, B. Mennucci, C. Adamo, D. Jacquemin, Choosing a functional for computing absorption and fluorescence band shapes with TD-DFT. *J. Chem. Theory Comput.* **2013**, *9*, 2749-2760.

[20] P. Pracht, F. Bohle, S. Grimme, Automated exploration of the low-energy chemical space with fast quantum chemical methods. *Phys. Chem. Chem. Phys.* **2020**, *22*, 7169-7192.

[21] C. Bannwarth, S. Ehlert, S. Grimme, GFN2-xTB—An accurate and broadly parametrized self-consistent tight-binding quantum chemical method with multipole electrostatics and density-dependent dispersion contributions. *J. Chem. Theory Comput.* **2019**, *15*, 1652-1671.
